# Contrasting Impacts of Photochemical and Microbial Processing on the Photoreactivity of Dissolved Organic Matter in an Adirondack Lake Watershed

**DOI:** 10.1021/acs.est.1c06047

**Published:** 2022-01-18

**Authors:** Joseph Wasswa, Charles T. Driscoll, Teng Zeng

**Affiliations:** Department of Civil and Environmental Engineering, Syracuse University, 151 Link Hall, Syracuse, New York 13244, United States

**Keywords:** DOM, photochemistry, reactive intermediates, browning, inland waters

## Abstract

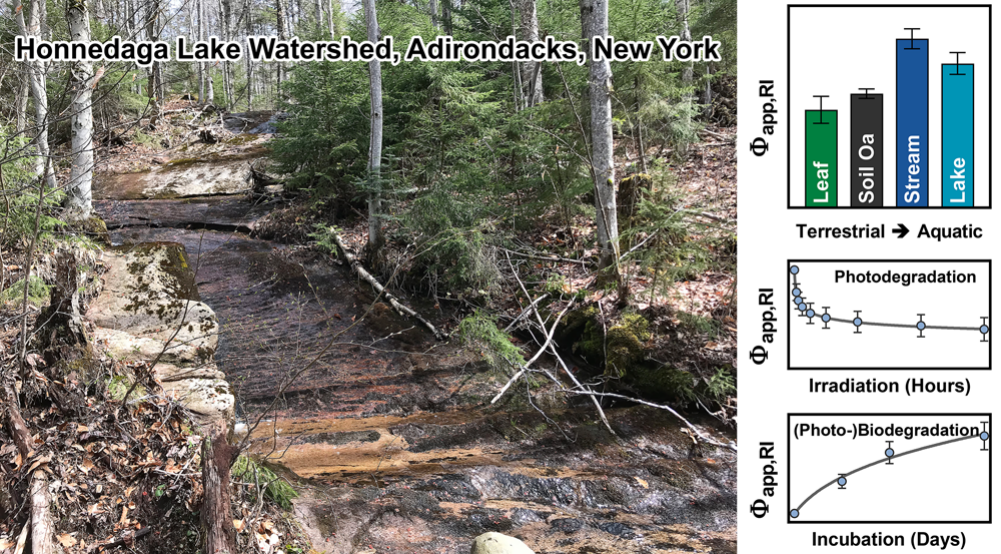

Photochemical and microbial processing are the prevailing mechanisms that shape the composition and reactivity of dissolved organic matter (DOM); however, prior research has not comparatively evaluated the impacts of these processes on the photoproduction of reactive intermediates (RIs) from freshly sourced terrestrial DOM. We performed controlled irradiation and incubation experiments with leaf and soil samples collected from an acid-impacted lake watershed in the Adirondack Mountain region of New York to examine the effects of DOM processing on the apparent quantum yields of RIs (Φ_app,RI_), including excited triplet states of DOM (^3^DOM*), singlet oxygen (^1^O_2_), and hydroxyl radicals (^•^OH). Photodegradation led to net reductions in Φ_app,^1^O_2__, Φ_app,^3^DOM*_, and Φ_app,^•^OH_, whereas (photo-)biodegradation resulted in increases in Φ_app,^1^O_2__ and Φ_app,^3^DOM*_. Photodegradation and (photo-)biodegradation also shifted the energy distribution of ^3^DOM* in different directions. Multivariate statistical analyses revealed the potential relevance of photo-biodegradation in driving changes in Φ_app,^1^O_2__ and Φ_app,^3^DOM*_ and prioritized five bulk DOM optical and redox properties that best explained the variations in Φ_app,^1^O_2__ and Φ_app,^3^DOM*_ along the watershed terrestrial-aquatic continuum. Our findings highlight the contrasting impacts of photochemical and microbial processes on the photoreactivity of freshly sourced terrestrial DOM and invite further studies to develop a more holistic understanding of their implications for aquatic photochemistry.

## Abstract 2

Photochemical and microbial processing exert divergent effects on the photoproduction of reactive intermediates from dissolved organic matter sourced from an acid-impacted Adirondack lake watershed.

## Introduction

Dissolved organic matter (DOM) is the most mobile and reactive fraction of organic matter that plays central roles in a myriad of biogeochemical processes in the terrestrial and aquatic environments.^1,2^ Multiple abiotic and biotic mechanisms such as photodegradation,^3−6^ biodegradation,^7−13^ and their couplings^14−23^ shape the optical properties,^24^ isotopic signatures,^25,26^ and molecular composition^27^ of DOM along the terrestrial-aquatic continuum. Furthermore, photochemical and microbial processing regulate the apparent quantum yields (Φ_app_) of primary and secondary photoproducts from DOM (e.g., via photomineralization or partial photooxidation of DOM^28−36^) in the photic zone of aquatic systems.^37^ Similarly, photochemical and microbial processing may dictate the Φ_app_ of transient reactive intermediates (RIs), such as excited triplet states of dissolved organic matter (^3^DOM*), singlet oxygen (^1^O_2_), and hydroxyl radicals (^•^OH). Most of these RIs are formed via the photosensitization of DOM and its interactions with inorganic constituents^38−40^ and participate in indirect photochemical processes that control the persistence and fate of carbon and contaminants in streams and lakes.^41−46^ Photooxidation alters the photoproduction of RIs from DOM isolates with concurrent changes in optical and redox properties;^47,48^ however, no prior research has systematically compared the effects of photochemical and microbial processing on the Φ_app_ of RIs (Φ_app,RI_) for freshly sourced terrestrial DOM.

Current global changes in hydroclimatic forcings and landscape characteristics have significant implications for process-driven formulations of DOM biogeochemistry in lake watersheds, particularly those undergoing recovery from anthropogenic acid deposition.^49−51^ For example, the Adirondack Mountain region of New York in the U.S. is characterized by watersheds and lakes historically impacted by high acid deposition. Substantial efforts have been made to investigate the variations in DOM flux and transport in Adirondack watersheds recovering from acidification^52−54^ as well as decadal-scale changes in DOM levels in downstream lakes (e.g., browning^55^) paralleled by declining atmospheric sulfur and nitrogen deposition.^55^ Several studies have further linked the lability of terrestrial DOM to its mass budget in these watersheds,^56−58^ but focused work is required to disentangle the specific impacts of abiotic and biotic modifications and their interplay on spatiotemporal shifts in DOM composition and reactivity. Subsequent incorporation of a fundamental understanding of these controls into process-based modeling will be integral to achieving a more robust characterization of DOM dynamics and function in Adirondack and other acid-impacted lake watersheds, which will ultimately support adaptive watershed management practices in response to projected natural and anthropogenic perturbations.

Leveraging the long-term biogeochemical research infrastructure in the Adirondacks, the objectives of this study were (i) to compare photochemically and microbially driven changes in Φ_app,RI_ for terrestrial DOM sourced from a pair of acid-impacted and limed lake watersheds in the Adirondacks; (ii) to examine the effects of photochemical and microbial processing of terrestrial DOM on the energy distribution of ^3^DOM*; and (iii) to constrain the significance of DOM processing in explaining the variations in Φ_app,RI_ along the watershed terrestrial-aquatic continuum. Recognizing that changes in Φ_app,RI_ depend on intrinsic DOM properties and their interactions with a multitude of environmental variables,^37^ this study did not aim to derive realistic estimates of controls over Φ_app,RI_ during *in situ* DOM processing in a temporally and spatially resolved manner; rather, we focused on tractable hypothetical scenarios assuming no replenishment of fresh DOM over a quantifiable time period to delineate the evolution of Φ_app,RI_.

## Materials and Methods

Chemical sources and reagent preparation are described in the Supporting Information.

### Field Sampling

Leaf litter, soil, stream water, and lake water samples were collected along the terrestrial-aquatic continuum of the Honnedaga Lake watershed in the Adirondacks (Figure S1). Similar to the majority of forested watersheds in the southwestern Adirondacks, the Honnedaga Lake watershed features thin-till catchments with poorly buffered soils^59^ and has received some of the highest atmospheric acid deposition in the recent past.^60^ Since the implementation of the Clean Air Act Amendments,^61^ Honnedaga Lake has undergone steady increases in surface water pH and acid neutralizing capacity with a slow recovery of the brook trout population.^62^ Experimental liming programs were implemented in several lake tributary watersheds between 2010 and 2016 to accelerate chemical and biological restoration,^63^ two of which were sampled in May 2018 as part of this study. Tributary watershed W16L (designated as “limed”) received a single dose of pelletized high-calcium limestone distributed by helicopter application in 2013.^62−64^ Tributary watershed W24R (designated as “reference”) features similar orientation and drainage area as W16L but did not receive any lime application. Leaf litter and soil samples, including the surface organic-rich horizon (designated as “Soil Oa”) and the uppermost mineral horizon (designated as “Soil Bs”), were collected from low, medium, and high elevation sites along three transects within W16L and W24R following established sampling protocols.^65^ Lastly, whole water samples were collected from W16L and W24R headwater streams and the epilimnion of Honnedaga Lake. Samples were transported in ice-chilled coolers to Syracuse University within 12 h. Leaf litter and soil samples from the surface organic-rich horizon were composited by site and extracted for water-extractable organic matter^66,67^ using a solution prepared to simulate the contemporary precipitation chemistry in the Adirondacks.^68^ Leachates and whole water samples were centrifuged, vacuum-filtered through 0.2 μm polyethersulfone membranes, and stored at 4 °C in the dark until use.

### Sample Treatment and Analysis

Filtered leaf and soil Oa leachate samples were adjusted to 15.9 ± 3.2 mg C/L of dissolved organic carbon (DOC) for parallel sunlight irradiation and dark incubation tests under oxic conditions. Standardized closed-system experiments were designed to enable optical and photochemical characterization of samples at reasonable time scales without logistical limitations rather than to provide quantitative estimates of DOM production and mineralization or to predict the effects of sunlight exposure and microbial metabolism on changes in the magnitude and rates of DOM processing at the watershed scale.

Sunlight irradiation tests were conducted in an Atlas Suntest XLS+(II) solar simulator equipped with a 1700 W xenon arc lamp and a daylight glass 300 nm UV filter. The lamp irradiance was controlled at 320 W/m^2^ between 300 and 800 nm to simulate the mid-May daily averaged solar irradiance in the Honnedaga Lake watershed at 43°N latitude, and the solar simulator chamber temperature was maintained at 25 ± 1 °C by an Atlas SunCool chiller. Leaf and soil Oa leachate samples were irradiated intermittently in cylindrical quartz vessels (20 cm × 28.4 mm i.d.) placed horizontally inside the solar simulator for 96 h with recurring light/dark cycles (designated as “photo” samples). Solutions (15.4 ± 0.9 mg C/L) of Suwannee River fulvic acid (SRFA; 3S101F) and Elliott Soil humic acid (ESHA; 5S102H) purchased from the International Humic Substance Society (IHSS) were also irradiated to allow comparison with previous work.^47^

Dark incubation tests were conducted in an Eppendorf Innova S44i biological shaker following the protocol adapted from previous studies.^12^ Leaf and soil Oa leachate samples were inoculated with 1% (v/v) of unfiltered headwater stream water and amended with nitrate and phosphate at an approximate C/N/P stoichiometry of 42:6:1^69^ to relieve possible nutrient limitation.^12,24,70^ Samples were then incubated in foil-wrapped baffled shake flasks with 0.2 μm vented polypropylene caps (to ensure constant, sterile air exchange) for 32 days at 20 ± 1 °C (designated as “bio” samples). Solutions of glucose (15.0 ± 0.9 mg C/L; a labile carbon source commonly used for soil and freshwater priming experiments^9,71−73^ and has been shown to fuel the microbial production of refractory DOM moieties^74−77^) and ESHA receiving the same inoculum and nutrient amendments were also incubated for comparison with leaf and soil Oa leachate samples. To assess the effects of photopriming,^78^ additional leaf and soil Oa leachate samples were irradiated in the solar simulator for 2 h prior to incubation in the biological shaker under the same conditions described above (designated as “photo-bio” samples).

Over the course of each experiment, subsamples were withdrawn from the quartz vessels or shake flasks at predetermined time intervals, re-filtered, and standardized to 4 mg C/L of DOC and pH 6.5 ± 0.1. For each subsample, the UV–visible absorbance spectra and fluorescence excitation-emission matrices were acquired on a Thermo Scientific Evolution 201 UV–visible spectrophotometer and a HORIBA Scientific Aqualog spectrofluorometer, respectively. Optical indices, such as the specific UV absorbance at 254 nm (SUVA_254_),^79^*E2*:*E3* (the ratio of Napierian absorption coefficients at 250 and 365 nm),^80^ fluorescence index (FI),^81,82^ freshness index (β:α),^83−85^ and the spectral slope coefficient *S*_290–400_,^86^ were extracted from the absorbance and fluorescence data using *MATLAB R2019a*. The antioxidant capacity (AOC) and the total phenolic content ([Phenolic]) were determined by the 2,2′-azinobis-(3-ethylbenzothiazoline-6-sulfonic acid) assay^87,88^ and the Folin-Ciocalteu assay,^89^ respectively. The concentrations of DOC, inorganic anions, base cations, and trace metals were also measured for all or selected samples. Major physicochemical characteristics and DOM properties of leachates and whole water samples are summarized in Tables S1–S4.

### Photochemistry Experiments

Steady-state photolysis experiments were performed in duplicate or triplicate using standardized leaf and soil leachates, photodegraded, biodegraded, or photo-biodegraded leaf and soil Oa leachates, as well as headwater stream and lake water samples. Prior to irradiation, each standardized sample ([DOC] = 4 mg C/L; pH 6.5 ± 0.1) was spiked with a specific probe compound to measure the formation of RIs, including furfuryl alcohol (FFA) for ^1^O_2_,^90,91^ 2,4,6-trimethylphenol (TMP) as an electron transfer probe for ^3^DOM* (^3^DOM_TMP_^*^),^92^*trans*,*trans*-2,4-hexadien-1-ol (*t*,*t*-HDO; sorbic alcohol) as an energy transfer probe for ^3^DOM* (^3^DOM_HDO_^*^),^93^ and terephthalic acid for ^•^OH^94,95^ (including lower-energy hydroxylating species^96−98^). To quantify the contribution of ^3^DOM* capable of sensitizing the isomerization of *t*,*t*-HDO (operationally designated as “high-energy ^3^DOM*”) to the formation of ^3^DOM* capable of oxidizing TMP and/or generating ^1^O_2_, *t*,*t*-HDO (2 mM) was spiked into standardized leachates and water samples (containing FFA or TMP) to quench ^3^DOM* with *E*_T_ of ≥250 kJ mol^–1^.^99,100^ Samples were then irradiated in quartz test tubes (100 mm × 11 mm i.d.; held at ∼30° from the horizontal) inside the solar simulator along with controls (i.e., to quantify direct photolysis and any nonphotochemical loss of probe compounds). Bimolecular *p*-nitroanisole/pyridine actinometer solutions were irradiated with each set of samples to monitor the incident light intensity.^101,102^ Solutions of eight IHSS DOM isolates ([DOC] = 4 mg C/L; pH 6.5 ± 0.1) were also irradiated for comparison with Honnedaga samples. Φ_app,^1^O_2__ (attributable to high-energy and low-energy ^3^DOM*), Φ_app,^3^DOM_TMP_^*^_ (attributable to high-energy and low-energy ^3^DOM*), Φ_app,^3^DOM_HDO_^*^_, and Φ_app,^•^OH_ for Honnedaga and IHSS samples were calculated over the wavelength range of 290–550 nm^103^ as detailed in the Supporting Information and summarized in Tables S7, S8, S11, and S14, respectively.

### Data Analysis

Gaussian error propagation was applied to estimate the uncertainties associated with calculations when applicable. Hierarchical cluster analysis (based on Euclidean distance with Ward’s method) was performed with the *z*-score standardized Φ_app,^1^O_2__ and Φ_app,^3^DOM*_ for Honnedaga samples using the *factoextra*(104) package in *R 4.0.3*, and the clustering pattern of samples was visualized on the first two principal component coordinates with confidence ellipses. Redundancy analysis was performed using the *vegan*(105) package in *R* with the Hellinger-transformed^106^ Φ_app,^1^O_2__ and Φ_app,^3^DOM*_ for Honnedaga samples as a matrix of response variables and selected DOM optical and redox properties as a matrix of explanatory variables. Prior to redundancy analysis, detrended correspondence analysis was first performed to confirm that Φ_app,^1^O_2__ and Φ_app,^3^DOM*_ exhibited a linear response to DOM properties (i.e., length values for the longest gradient were less than 2). Forward selection was implemented to obtain a most parsimonious model until the variance inflation factors for all explanatory variables were less than 2.5. The significance of variables and axes was tested using the permutational (999 iterations) analysis of variance. Multiple linear regression, nonlinear least squares regression, and other statistical analyses were performed using *GraphPad Prism 8.4*.

## Results and Discussion

### Magnitude and Patterns of Photoreactivity

**Figure 1:**
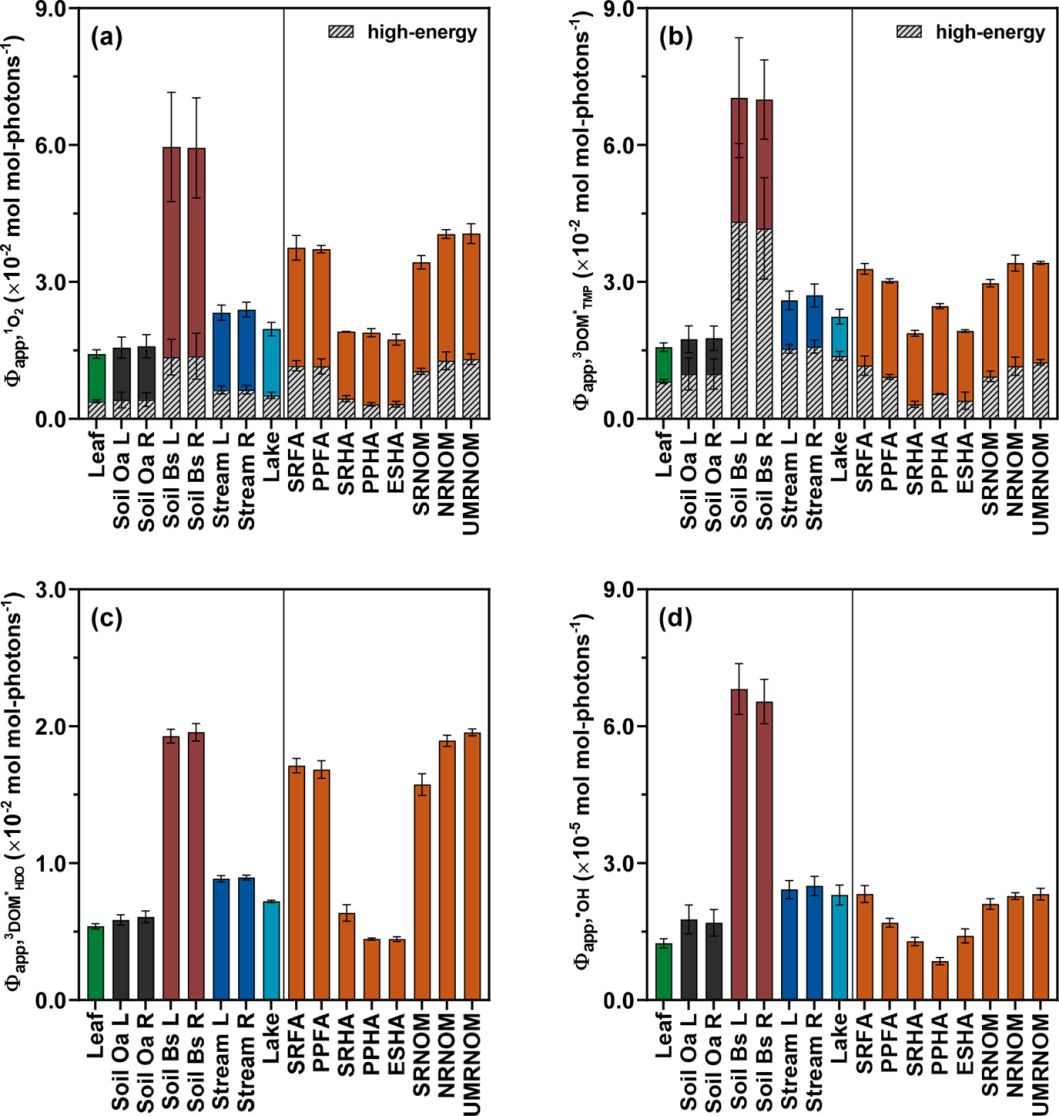
Comparison of Φ_app,RI_ for leachates and whole water samples from the Honnedaga Lake watershed and DOM isolates supplied by the International Humic Substances Society (IHSS): (a) Φ_app,^1^O_2__ (attributable to Φ_app,^1^O_2_,high-energy_ and Φ_app,^1^O_2_,low-energy_) for the Honnedaga samples and IHSS DOM isolates. (b) Φ_app,^3^DOM_TMP_^*^_ (attributable to Φ_app,^3^DOM_TMP_^*^,high-energy_ and Φ_app,^3^DOM_TMP_^*^,low-energy_) for the Honnedaga samples and IHSS DOM isolates. (c) Φ_app,^3^DOM_HDO_^*^_ for the Honnedaga samples and IHSS DOM isolates. (d) Φ_app,^•^OH_ for the Honnedaga samples and IHSS DOM isolates. For soil Oa and Bs leachates, indices “L” and “R” refer to W16L (limed) and W24R (reference) tributary watersheds, respectively. Φ_app,RI_ for soil Oa and BS leachates from three different elevations within the same tributary watershed were pooled for the clarity of presentation as there was no significant difference among these three sample groups. Φ_app,RI_ for Suwannee River fulvic acid (SRFA; 3S101F), Pahokee Peat fulvic acid (PPFA; 2S103F), Suwannee River humic acid (SRHA; 3S101H), Pahokee Peat humic acid (PPHA; 1S103H), Elliott Soil humic acid (ESHA; 5S102H), Suwannee River natural organic matter (SRNOM; 2R101N), Nordic Reservoir natural organic matter (NRNOM; 1R108N), and Upper Mississippi River natural organic matter (UMRNOM; 1R110N) were measured under the same standardized irradiation and solution conditions ([DOC] = 4 mg C/L; pH 6.5 ± 0.1). Error bars represent the standard deviations from duplicate or triplicate measurements.

Φ_app,RI_ (i.e., Φ_app,^1^O_2__, Φ_app,^3^DOM_TMP_^*^_, Φ_app,^3^DOM_HDO_^*^_, and Φ_app,^•^OH_) for native leachates and water samples from the Honnedaga Lake watershed were on the same order of magnitude as those reported for DOM extracts or fractions isolated from other terrestrial and aquatic environments as well as those measured for lake water samples from the Adirondack Region.^103^ For example, Φ_app,^1^O_2__ for Honnedaga samples varied from 1.4 × 10^–2^ to 6.1 × 10^–2^ with a median of 2.1 × 10^–2^, which overlapped with the range of values for eight IHSS DOM isolates measured under the same irradiation conditions (Figure 1a). Φ_app,^1^O_2__ attributable to high-energy ^3^DOM* (Φ_app,^1^O_2_,high-energy_) varied from 0.4 × 10^–2^ to 1.4 × 10^–2^ (Table S8), whereas Φ_app,^1^O_2__ attributable to low-energy ^3^DOM* (Φ_app,^1^O_2_,low-energy_) were approximately 3-fold higher, ranging from 1.0 × 10^–2^ to 4.7 × 10^–2^. On average, the percent contribution of high-energy ^3^DOM* to Φ_app,^1^O_2__ was 25 ± 2% (Table S9), which fell on the lower end of values measured for the IHSS DOM isolates (i.e., 20–48%). Φ_app,^3^DOM_TMP_^*^_ varied from 1.6 × 10^–2^ to 7.2 × 10^–2^ with a median of 2.4 × 10^–2^, which was not statistically different from the median value measured for the IHSS DOM isolates (Mann–Whitney *U* test *p* = 0.4164) (Figure 1b). Φ_app,^3^DOM_TMP_^*^_ attributable to high-energy ^3^DOM* (Φ_app,^3^DOM_TMP_^*^,high-energy_) ranged from 0.8 × 10^–2^ to 4.5 × 10^–2^ (Table S11), which were approximately 1.4-fold higher than Φ_app,^3^DOM_TMP_^*^_ attributable to low-energy ^3^DOM* (Φ_app,^3^DOM_TMP_^*^,low-energy_). On average, the percent contribution of high-energy ^3^DOM* to Φ_app,^3^DOM_TMP_^*^_ was 58 ± 3% (Table S12), consistent with prior work showing that a major fraction of high-energy ^3^DOM* also participated in one-electron transfer TMP oxidation.^100^ Compared to Φ_app,^3^DOM_TMP_^*^_, Φ_app,^3^DOM_HDO_^*^_ for corresponding leachates and water samples were 69 ± 3% lower, ranging from 0.5 × 10^–2^ to 2.0 × 10^–2^ with a median of 0.8 × 10^–2^ (Figure 1c). Φ_app,^1^O_2__, Φ_app,^3^DOM_TMP_^*^_, and Φ_app,^3^DOM_HDO_^*^_ showed strong correlations with each other (Spearman correlation coefficient ρ = 0.940–0.969; *p* < 0.001) as FFA, TMP, and *t*,*t*-HDO probes different but overlapping pools of ^3^DOM*.^39,99,100,107,108^ The Φ_app,^1^O_2__ to Φ_app,^3^DOM_TMP_^*^_ ratio varied from 0.84 to 0.91, which fell within the theoretical range predicted from the O_2_-dependent quenching of ^3^DOM*.^39^ The Φ_app,^3^DOM_HDO_^*^_ to Φ_app,^1^O_2_,high-energy_ ratio and the Φ_app,^3^DOM_HDO_^*^_ to Φ_app,^3^DOM_TMP_^*^,high-energy_ ratio averaged 1.42 ± 0.02 and 0.54 ± 0.08, respectively, confirming that high-energy ^3^DOM* that sensitized *t*,*t*-HDO isomerization encompassed those capable of ^1^O_2_ generation but did not necessarily participate in TMP oxidation. Furthermore, the ^1^O_2_ yield from the O_2_-dependent quenching of ^3^DOM_HDO_^*^ averaged 0.98 ± 0.02 for leachate and water samples (Table S15), which closely matched the yields measured for the IHSS DOM isolates (i.e., 0.95 ± 0.03). Φ_app,^•^OH_ spanned a range of 1.2 × 10^–5^ to 6.9 × 10^–5^ with a median of 2.4 × 10^–5^, which was comparable to the median value measured for the IHSS DOM isolates (Mann–Whitney *U* test *p* = 0.1833; Figure 1d) and showed positive correlations with Φ_app,^1^O_2__, Φ_app,^3^DOM_TMP_^*^_, and Φ_app,^3^DOM_HDO_^*^_ (ρ = 0.783–0.838; *p* < 0.001). However, the role of ^3^DOM* (e.g., those capable of generating ^1^O_2_, oxidizing TMP, and/or sensitizing *t*,*t*-HDO isomerization) in ^•^OH production remains debated because conflicting data existed for the lack of correlation between Φ_app,^•^OH_ and Φ_app,^1^O_2__ or Φ_app,^3^DOM*_^103,109,110^ and pathways involving non-^3^DOM* species (e.g., charge-separated DOM species formed by the donor–acceptor electron transfer^111^ or excited state oxidants of unknown identity^47,98^) might contribute to ^•^OH production.^38^

On average, Φ_app,RI_ were lower for leaf and soil Oa leachates, intermediate for stream and lake water samples, and higher for soil Bs leachates. Φ_app,RI_ for soil leachates and stream water samples from the limed and reference tributary watersheds were not statistically different (Tukey’s multiple comparisons test *p* > 0.9999). Φ_app,RI_ for leachates from low, medium, and high elevations within the same tributary watershed were not statistically different either (Tukey’s multiple comparisons test *p* > 0.9999), suggesting that liming did not exert long-term impacts on the photoreactivity of DOM at inter- and intra-watershed scales over the post-application period. Φ_app,^1^O_2__, Φ_app,^3^DOM_TMP_^*^_, Φ_app,^3^DOM_HDO_^*^_, and Φ_app,^•^OH_ for soil Bs leachates were 232 ± 24% to 302 ± 96% higher than those for soil Oa leachates from the same sites, which might in part be rationalized by the greater degree of microbial processing of DOM^112^ in the uppermost mineral soil horizon as evidenced by the higher FI and β:α values measured for soil Bs leachates (Mann–Whitney *U* test *p* < 0.0001). Furthermore, Φ_app,^1^O_2__, Φ_app,^3^DOM_TMP_^*^_, Φ_app,^3^DOM_HDO_^*^_, and Φ_app,^•^OH_ for leachates and water samples all showed positive correlations with SUVA_254_ (Spearman correlation coefficient ρ = 0.488–0.634; *p* = 0.0020–0.0249), FI (ρ = 0.612–0.804; *p* = <0.0001–0.0032), and β:α (ρ = 0.664–0.857; *p* = <0.0001–0.0010) but negative correlations with AOC (ρ = −0.907 to −0.692; *p* = <0.0001–0.0005) and [Phenolic] (ρ = −0.833 to −0.739; *p* ≤ 0.0001), which were likely dictated by the photochemical and microbial processing of DOM along the terrestrial-aquatic continuum in the Honnedaga Lake watershed.

### Effects of Photodegradation on Photoreactivity

**Figure 2:**
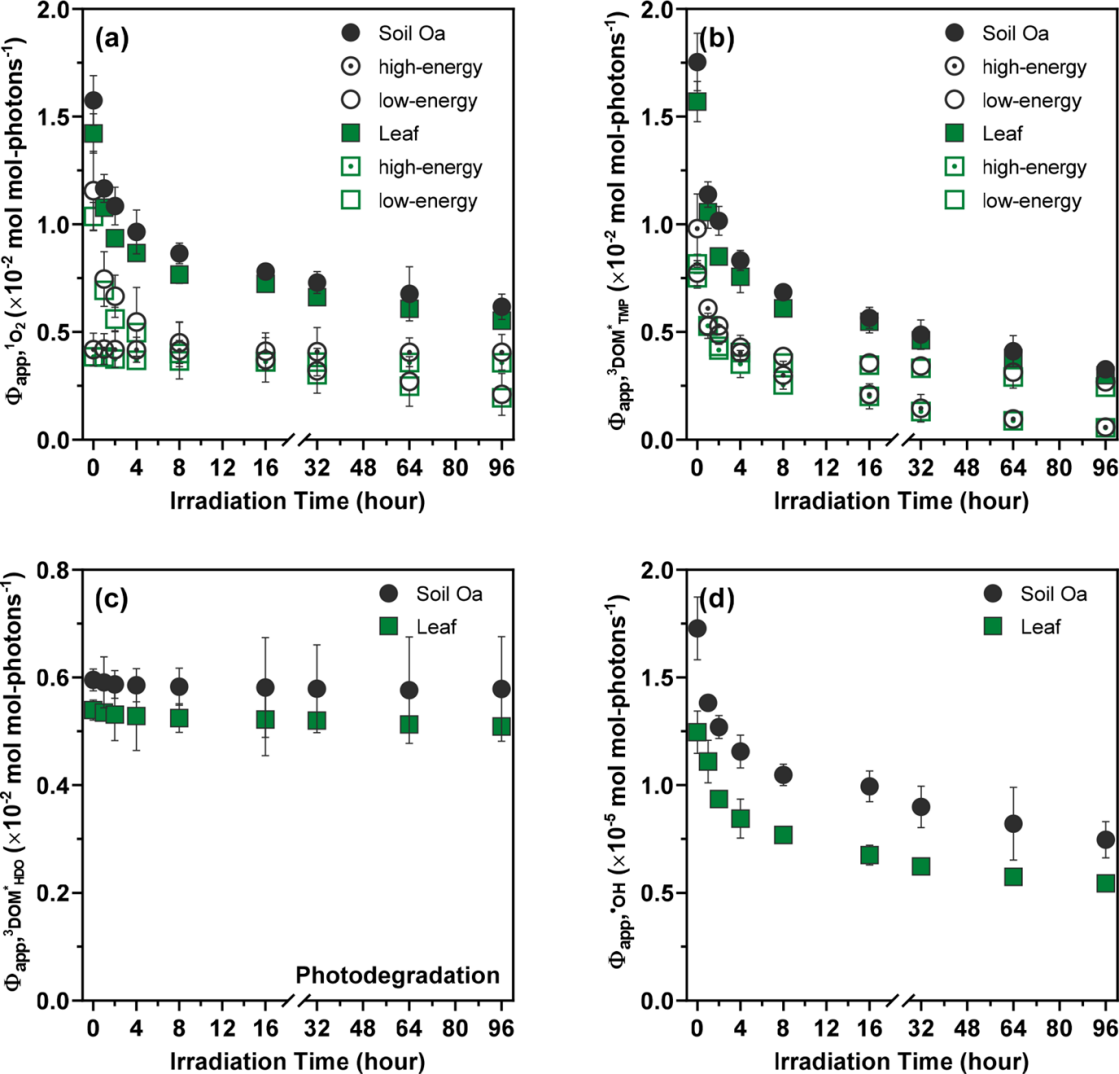
Changes in Φ_app,RI_ during photodegradation of leaf and soil Oa leachate samples: (a) Changes in Φ_app,^1^O_2__ (attributable to Φ_app,^1^O_2_,high-energy_ and Φ_app,^1^O_2_,low-energy_) for leaf and soil Oa leachates over 96 h of simulated sunlight irradiation. (b) Changes in Φ_app,^3^DOM_TMP_^*^_ (attributable to Φ_app,^3^DOM_TMP_^*^,high-energy_ and Φ_app,^3^DOM_TMP_^*^,low-energy_) for leaf and soil Oa leachates over 96 h of simulated sunlight irradiation. (c) Changes in Φ_app,^3^DOM_HDO_^*^_ for leaf and soil Oa leachates over 96 h of simulated sunlight irradiation. (d) Changes in Φ_app,^•^OH_ for leaf and soil Oa leachates over 96 h of simulated sunlight irradiation. Φ_app,RI_ for native and photodegraded samples were measured under standardized irradiation and solution conditions ([DOC] = 4 mg C/L; pH 6.5 ± 0.1). Φ_app,RI_ for soil Oa leachates from W16L (limed) and W24R (reference) tributary watersheds were pooled for the clarity of presentation as there was no significant difference between these two sample groups. Error bars represent the standard deviations from duplicate or triplicate measurements; where absent, bars fall within symbols.

Over the course of photodegradation, DOC, SUVA_254_, [Phenolic], and AOC of leaf and soil Oa leachates decreased by 36 ± 11%, 38 ± 4%, 57 ± 23%, and 21 ± 4%, respectively, while *E2:E3* increased by 66 ± 4%, reflecting the decomposition of higher molecular weight aromatic moieties with a progressive loss of antioxidant properties within leaf and soil Oa DOM during irradiation. Φ_app,^1^O_2__ and Φ_app,^3^DOM_TMP_^*^_ for leaf and soil Oa leachates decreased rapidly with comparable initial apparent first-order decay coefficients (Table S16) and continued to decrease by 61 ± 9% and 81 ± 11%, respectively, after 96 h of irradiation (Figure 2a,b). The second-order reaction rate constant of TMP with ^3^DOM* (*k*_TMP,^3^DOM_TMP_^*^_) measured for extensively photodegraded samples (i.e., 7.9 ± 1.3 × 10^8^ M^–1^ s^–1^; Table S10) did not differ significantly from those measured for native samples (i.e., 8.8 ± 1.5 × 10^8^ M^–1^ s^–1^), suggesting that changes in Φ_app,^3^DOM_TMP_^*^_ during irradiation were not driven by the inhibition of ^3^DOM*-induced TMP oxidation.^109^ Such decreases in Φ_app,^1^O_2__ and Φ_app,^3^DOM_TMP_^*^_ during irradiation of leaf and soil Oa DOM likely stemmed from two contrasting effects, with the former outcompeting the latter: (1) the photochemical destruction of aromatic DOM moieties (e.g., aromatic ketones and quinones^108,113−116^) that served as precursors to ^3^DOM* capable of oxidizing TMP and/or generating ^1^O_2_ and (2) the diminished probability of intramolecular charge–transfer complex formation and/or intramolecular ^3^DOM* reduction due to the decomposition of DOM moieties with antioxidant properties (e.g., phenols^117,118^).^47,109^ Φ_app,^3^DOM_HDO_^*^_ for leaf and soil Oa leachates, although exhibiting positive correlations with Φ_app,^1^O_2__ and Φ_app,^3^DOM_TMP_^*^_ (ρ = 0.513–0.560; *p* = 0.0005–0.0016), only decreased by 4 ± 2% after 96 h of irradiation (Figure 2c), indicating that moieties producing high-energy ^3^DOM* capable of sensitizing *t*,*t*-HDO isomerization were relatively resistant to photodegradation and not readily produced via photochemical alteration of native DOM. Like *k*_TMP,^3^DOM_TMP_^*^_, the second-order reaction rate constant of *t*,*t*-HDO with ^3^DOM* (*k*_*t*,*t*-HDO,^3^DOM_HDO_^*^_) measured for extensively photodegraded samples (i.e., 8.1 ± 2.2 × 10^8^ M^–1^ s^–1^; Table S13) were not statistically different from those measured for native samples (i.e., 8.9 ± 3.4 × 10^8^ M^–1^ s^–1^), pointing toward the convergence of ^3^DOM* reactivity with TMP and *t*,*t*-HDO regardless of prior sample irradiation history. Lastly, Φ_app,^•^OH_ decreased by 57 ± 9% after 96 h of irradiation (Figure 2d), which corroborated prior work reporting decreases in Φ_app,^•^OH_ for three IHSS DOM isolates upon photobleaching.^47^ Φ_app,^•^OH_ also showed positive correlations with Φ_app,^3^DOM_TMP_^*^_ and Φ_app,^3^DOM_HDO_^*^_ (ρ = 0.645–0.908; *p* < 0.0001) despite its lower initial apparent first-order decay coefficient during irradiation (Table S16); however, the role of ^3^DOM* in ^•^OH production and the contribution of H_2_O_2_-dependent (e.g., involving one-electron reduction of O_2_ by ^3^DOM* to form O_2_^•–^ that subsequently undergoes dismutation to produce H_2_O_2_)^111,119^ and H_2_O_2_-independent pathways (e.g., involving H-atom abstraction from water by ^3^DOM*)^96,120^ to ^•^OH production warrant further investigation.

Comparative irradiation experiments performed using standardized SRFA and ESHA solutions showed that Φ_app,^1^O_2__, Φ_app,^3^DOM_TMP_^*^_, and Φ_app,^•^OH_ all decreased monotonically as a function of irradiation time (Figure S6). However, previous work examining changes in the photoreactivity of SRFA and ESHA observed decreases in *f*_TMP_ (i.e., the quantum yield coefficient of ^3^DOM* with TMP) and Φ_app,^•^OH_ but increases in Φ_app,^1^O_2__ with prolonged photobleaching.^47^ Such contradictory observations with respect to Φ_app,^1^O_2__ probably arose from the use of different batches of IHSS DOM isolates or methodological differences in quantifying ^1^O_2_ formation efficiency (e.g., the use of monochromatic light at 365 nm^47^ vs simulated sunlight in this work) considering the wavelength dependence of Φ_app,^1^O_2__.^121^ Consistent with the patterns found for leaf and soil Oa DOM, an earlier study also reported the decreased formation of ^3^DOM*, ^1^O_2_, and ^•^OH from aquatic DOM and wastewater effluent organic matter upon photobleaching under simulated sunlight.^48^ Somewhat in contrast to the above findings, a more recent study showed increases in Φ_app,^3^DOM_TMP_^*^_ following photobleaching of stormflow samples collected from vegetated and developed upper Mississippi River watersheds under simulated sunlight,^122^ again highlighting the challenge to reconcile and generalize photoreactivity trends across studies given the varying DOM source and composition.

Photodegradation also altered the relative distribution of Φ_app,^1^O_2__ and Φ_app,^3^DOM_TMP_^*^_ attributable to high-energy and low-energy ^3^DOM*. On average, the percent contribution of high-energy ^3^DOM* to Φ_app,^1^O_2__ increased from 27 ± 10% to 65 ± 14% for leaf and soil Oa DOM (Figure S9), whereas the percent contribution of high-energy ^3^DOM* to Φ_app,^3^DOM*_ decreased from 54 ± 7% to 18 ± 9% after 96 h of irradiation (Figure S10). Hypothetically, Φ_app,^1^O_2__ and Φ_app,^3^DOM_TMP_^*^_ for photodegraded samples could be attributed to ^3^DOM* formed by photo-resistant (relative to the irradiation conditions adopted in this work) and/or photo-altered DOM moieties.^123,124^ Φ_app,^1^O_2_,high-energy_ did not vary significantly throughout irradiation and sustained an increasing share in Φ_app,^1^O_2__ with increasing irradiation, whereas Φ_app,^1^O_2_,low-energy_ decreased by 82 ± 22% after irradiation (Figure 2a), suggesting that moieties producing high-energy ^3^DOM* capable of ^1^O_2_ generation were more photo-resistant than those producing low-energy ^3^DOM* capable of ^1^O_2_ generation and/or photo-altered moieties were less efficient in producing low-energy ^3^DOM* capable of ^1^O_2_ generation than photo-resistant moieties. Furthermore, Φ_app,^3^DOM_TMP_^*^,high-energy_ decreased by a greater extent than Φ_app,^3^DOM_TMP_^*^,low-energy_ after irradiation (i.e., 94 ± 17% vs 66 ± 8%; Figure 2b), indicating that moieties producing high-energy ^3^DOM* that participated in TMP oxidation were preferentially destructed with increasing irradiation relative to those producing low-energy ^3^DOM* that participated in TMP oxidation and/or photo-altered moieties exhibited an overall lower formation efficiency of high-energy ^3^DOM* participating in TMP oxidation compared to photo-resistant moieties.

### Effects of Biodegradation and Photo-Biodegradation on Photoreactivity

**Figure 3:**
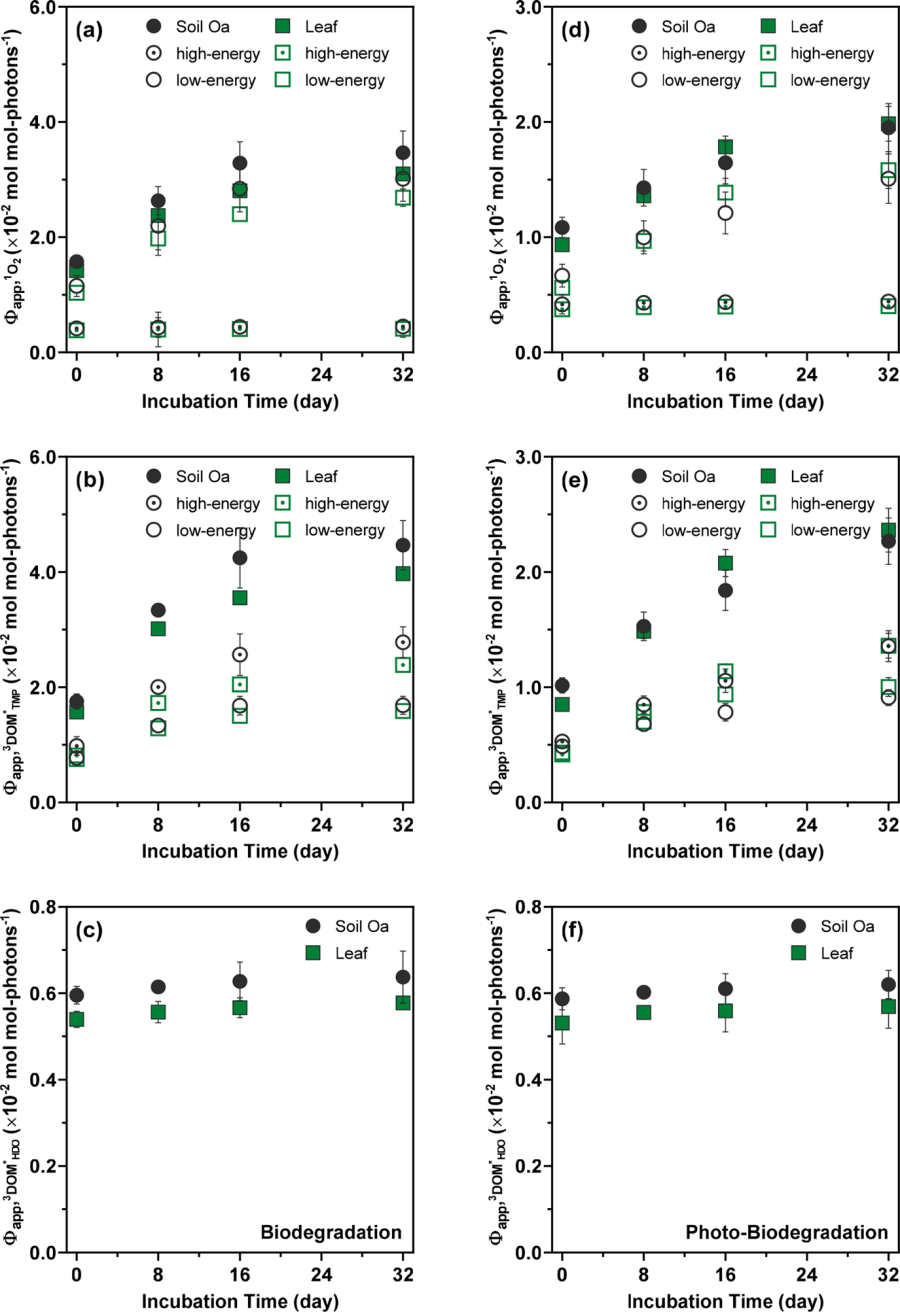
Changes in Φ_app,RI_ during biodegradation and photo-biodegradation of leaf and soil Oa leachate samples: (a) Changes in Φ_app,^1^O_2__ (attributable to Φ_app,^1^O_2_,high-energy_ and Φ_app,^1^O_2_,low-energy_) for leaf and soil Oa leachates over 32 d of dark incubation. (b) Changes in Φ_app,^3^DOM_TMP_^*^_ (attributable to Φ_app,^3^DOM_TMP_^*^,high-energy_ and Φ_app,^3^DOM_TMP_^*^,low-energy_) for leaf and soil Oa leachates over 32 d of incubation. (c) Changes in Φ_app,^3^DOM_HDO_^*^_ for leaf and soil Oa leachates over 32 d of incubation. (d) Changes in Φ_app,^1^O_2__ (attributable to Φ_app,^1^O_2_,high-energy_ and Φ_app,^1^O_2_,low-energy_) for leaf and soil Oa leachates over 32 d of dark incubation with 2 h of prior irradiation. (e) Changes in Φ_app,^3^DOM_TMP_^*^_ (attributable to Φ_app,^3^DOM,high-energy_ and Φ_app,^3^DOM_TMP_^*^,low-energy_) for leaf and soil Oa leachates over 32 d of incubation with 2 h of prior irradiation. (f) Changes in Φ_app,^3^DOM_HDO_^*^_ for leaf and soil Oa leachates over 32 d of incubation with 2 h of prior irradiation. Φ_app,RI_ for native and biodegraded/photo-biodegraded samples were measured under standardized irradiation and solution conditions ([DOC] = 4 mg C/L; pH 6.5 ± 0.1). Φ_app,RI_ for soil Oa leachates from W16L (limed) and W24R (reference) tributary watersheds were pooled for the clarity of presentation as there was no significant difference between these two sample groups. Error bars represent the standard deviations from duplicate measurements; where absent, bars fall within symbols.

Over the course of biodegradation, SUVA_254_, [Phenolic], and AOC of leaf and soil Oa leachates increased by 21 ± 3%, 22 ± 5%, and 185 ± 40%, respectively, while DOC and *E2:E3* only decreased by <10% (i.e., 6 ± 1% and 8 ± 1%, respectively), suggesting the enrichment of aromatic DOM moieties with a concomitant increase in the antioxidant content within leaf and soil Oa DOM during incubation. Exposing native leaf and soil Oa leachates to simulated sunlight prior to incubation promoted more pronounced changes in DOM properties indicative of aromaticity and antioxidant potential. For example, SUVA_254_, [phenolic], and AOC increased by 31 ± 7%, 53 ± 14%, and 240 ± 50%, respectively, over the course of photo-biodegradation. On average, Φ_app,^1^O_2__ and Φ_app,^3^DOM_TMP_^*^_ for leaf and soil Oa leachates increased by 108 ± 19% and 152 ± 22%, respectively, after 32 d of incubation (Figure 3a,b) or post-irradiation incubation (Figure 3d,e). Such increases in Φ_app,^1^O_2__ and Φ_app,^3^DOM_TMP_^*^_ during incubation of leaf and soil Oa DOM presumably originated from two competing effects, with the former outweighing the latter: (1) the microbial production of aromatic DOM moieties that served as precursors to ^3^DOM* capable of generating ^1^O_2_ and/or oxidizing TMP and (2) the enhanced probability of intramolecular charge–transfer complex formation and/or intramolecular ^3^DOM* reduction due to the enrichment of antioxidant moieties. Φ_app,^3^DOM_HDO_^*^_ showed positive correlations with Φ_app,^1^O_2__ and Φ_app,^3^DOM_TMP_^*^_ (ρ = 0.570–0.573; *p* = 0.0006–0.0007) but only underwent 6 ± 1% increases after 32 d of incubation or post-irradiation incubation (Figure 3c,f), indicating that DOM moieties producing high-energy ^3^DOM* capable of sensitizing *t*,*t*-HDO isomerization were recalcitrant to biodegradation and not produced via microbial processing of DOM. Furthermore, *k*_TMP,^3^DOM_TMP_^*^_ (i.e., 9.4 ± 1.7 × 10^8^ M^–1^ s^–1^; Table S9) and *k*_*t*,*t*-HDO,^3^DOM_HDO_^*^_ (i.e., 9.3 ± 2.8 × 10^8^ M^–1^ s^–1^; Table S11) measured for extensively (photo-)biodegraded samples did not differ significantly from those measured for native or extensively photodegraded samples, providing further evidence for the limited variability in ^3^DOM* reactivity with TMP or *t*,*t*-HDO introduced by incubation or irradiation. Lastly, Φ_app,^•^OH_ also increased after 32 d of incubation or post-irradiation incubation (Figure S8), but photolysis of residual NO_3_^–^ from the initial nutrient amendment (e.g., 0.77 ± 0.06 mg NO_3_^–^/mg C) might contribute to ^•^OH production^125−127^ and confound trend interpretations.

Comparative incubation experiments using glucose and ESHA as endmember model DOM on the bio-lability spectrum provided additional support for the microbially mediated increases in Φ_app,^1^O_2__ and Φ_app,^3^DOM_TMP_^*^_. Compared to leaf and soil Oa DOM, Φ_app,^1^O_2__ and Φ_app,^3^DOM_TMP_^*^_ barely changed during incubation with the more bio-refractory ESHA but increased by over an order of magnitude after 32 d of incubation with glucose as the sole carbon source (Figure S7), supporting the hypothesis that glucose fueled the microbial production of photoreactive moieties, some of which might serve as precursors to ^3^DOM*. Furthermore, Φ_app,^3^DOM_HDO_^*^_ was not statistically different from zero in fresh glucose solutions but increased substantially to approximately 3-fold higher than those measured for leaf and soil Oa leachates after 32 d of incubation. Previous work characterizing the photoreactivity of water samples from temperate wetlands in the Midwestern U.S. also attributed increasing trends in Φ_app,^3^DOM_TMP_^*^_ to microbial processing of vascular plant-derived DOM and/or production of autochthonous DOM within the wetland watersheds, but the argument for microbial action was formulated primarily based on the correlations between Φ_app,^3^DOM_TMP_^*^_ and watershed characteristics.^128^ Together with the patterns observed with leaf and soil Oa DOM, results from the glucose incubation experiment consolidated the findings in the literature with respect to the enhanced photoreactivity conferred by microbially derived DOM such as wastewater effluent organic matter^129−132^ and extracellular polymeric substances released by heterotrophic bacteria.^133^

Like photodegradation, biodegradation and photo-biodegradation also shifted the relative distribution of Φ_app,^1^O_2__ and Φ_app,^3^DOM_TMP_^*^_ attributable to high-energy and low-energy ^3^DOM*. On average, the percent contribution of high-energy ^3^DOM* to Φ_app,^1^O_2__ decreased from 33 ± 9% to 17 ± 6% for leaf and soil Oa DOM (Figure S9), whereas the percent contribution of high-energy ^3^DOM* to Φ_app,^3^DOM_TMP_^*^_ increased from 52 ± 2% to 60 ± 2% after 32 d of incubation or post-irradiation incubation (Figure S10). Φ_app,^1^O_2__ and Φ_app,^3^DOM_TMP_^*^_ for (photo-)biodegraded samples could be attributed to ^3^DOM* formed by bio-refractory (i.e., relative to the incubation conditions adopted in this work) and/or bio-transformed DOM moieties.^134,135^ Φ_app,^1^O_2_,high-energy_ remained largely unchanged after incubation, supporting the notion that moieties producing high-energy ^3^DOM* capable of ^1^O_2_ generation were bio-refractory. In contrast, Φ_app,^1^O_2_,low-energy_ increased by 150 ± 60% after incubation, suggesting that bio-transformed moieties were more efficient in producing low-energy ^3^DOM* capable of ^1^O_2_ generation, thereby leading to an elevated contribution of Φ_app,^1^O_2_,low-energy_ to Φ_app,^1^O_2__. Moreover, Φ_app,^3^DOM_TMP_^*^,high-energy_ increased more substantially than Φ_app,^3^DOM_TMP_^*^,low-energy_ (i.e., 188 ± 60% vs 111 ± 27%) after incubation, indicating that moieties producing high-energy ^3^DOM* that participated in TMP oxidation were more actively produced by continual microbial processing relative to moieties producing low-energy ^3^DOM* that participated in TMP oxidation and/or bio-transformed moieties exhibited an overall lower formation efficiency of low-energy ^3^DOM* participating in TMP oxidation than bio-refractory moieties. Taken together, these data demonstrated that (photo-)biodegradation and photodegradation exerted contrasting effects on changes in the magnitude of Φ_app,^1^O_2__ and Φ_app,^3^DOM_TMP_^*^_ as well as the relative contribution of high-energy and low-energy ^3^DOM* to Φ_app,^1^O_2__ and Φ_app,^3^DOM_TMP_^*^_. Still, an in-depth analysis of DOM by ultrahigh-resolution mass spectrometry and high-field nuclear magnetic resonance spectroscopy would be required to elucidate the compositional and structural characteristics of photo-resistant/altered and bio-refractory/transformed moieties in relation to their photoreactivity.

### Photoreactivity Variation Driven by DOM Processing

**Figure 4:**
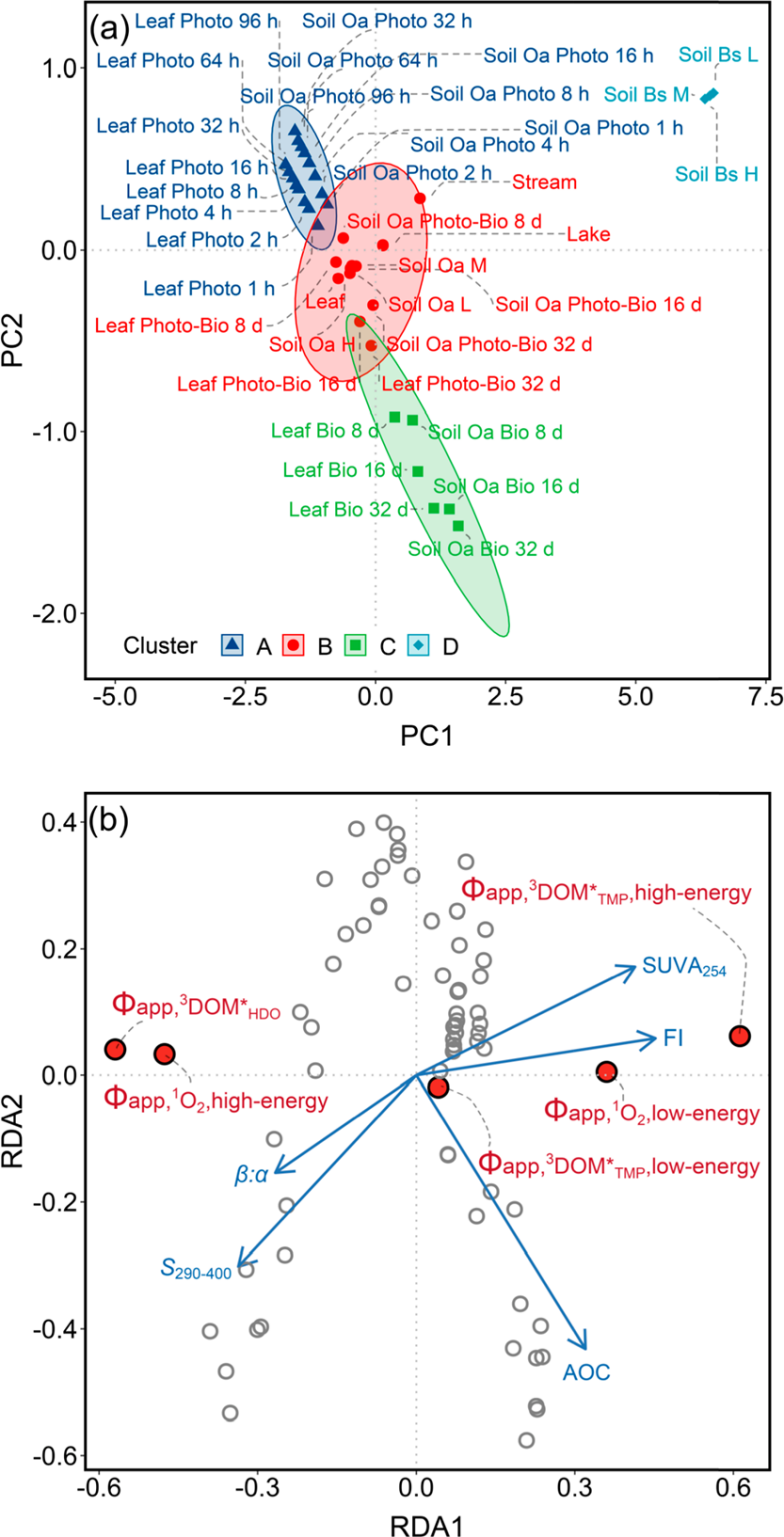
Multivariate statistical analyses of Φ_app,^1^O_2__ and Φ_app,^3^DOM*_ for leaf and soil leachates and whole water samples from the Honnedaga Lake watershed: (a) Cluster plot of the *z*-score standardized Φ_app,^1^O_2_,high-energy_, Φ_app,^1^O_2_,low-energy_, Φ_app,^3^DOM_TMP_^*^,high-energy_, Φ_app,^3^DOM_TMP_^*^,low-energy_, and Φ_app,^3^DOM_HDO_^*^_ on the first two principal component coordinates. Φ_app,RI_ for soil Oa and Bs leachates and stream water samples from W16L (limed) and W24R (reference) tributary watersheds were pooled for the analysis as there was no significant difference between these two sample groups. For soil Oa and Bs leachates, indices “L”, “M”, and “H” refer to “low elevation” and “medium elevation”, and “high elevation”, respectively. Samples are grouped into four clusters (i.e., cluster A, B, C, and D, respectively) based on 95% confidence ellipses. Note that the *x* and *y* axes do not have equal scales. (b) Redundancy analysis ordination plot of the Hellinger-transformed Φ_app,^1^O_2_,high-energy_, Φ_app,^1^O_2_,low-energy_, Φ_app,^3^DOM_TMP_^*^,high-energy_, Φ_app,^3^DOM_TMP_^*^,low-energy_, and Φ_app,^3^DOM_HDO_^*^_ constrained by five DOM properties, including fluorescence index (FI), the specific UV absorbance at 254 nm (SUVA_254_), the spectral slope coefficient *S*_290–400_, antioxidant capacity (AOC), and freshness index (β:α). Vectors represent explanatory variables. Filled red circles represent response variables. Grey circles represent individual samples. Note that the *x* and *y* axes do not have equal scales.

To assess the relevance of photochemical and microbial processing for constraining changes in DOM photoreactivity in the Honnedaga Lake watershed, hierarchical cluster analysis was performed using the *z*-score standardized Φ_app,^1^O_2_,high-energy_, Φ_app,^1^O_2_,low-energy_, Φ_app,^3^DOM_TMP_^*^,high-energy_, Φ_app,^3^DOM_TMP_^*^,low-energy_, and Φ_app,^3^DOM_HDO_^*^_ for native, photodegraded, and (photo-)biodegraded leachates and stream and lake water samples. Hierarchical clustering grouped leachates and water samples into four clusters (Figure 4a). Cluster A includes all photodegraded leaf and soil Oa leachates that clustered toward the upper left of the ordination space. Cluster B consists of native and photo-biodegraded leaf and soil Oa leachates plus stream and lake water samples. Cluster C contains all biodegraded leaf and soil Oa leachates that clustered in the opposite direction to Cluster A samples. Cluster D, on the other hand, includes only soil Bs leachates and was well separated from the other three clusters in ordination space. The fact that stream and lake water samples clustered most closely with native and photo-biodegraded leaf and soil Oa leachates underscored that leaf litter and the surface organic-rich soil horizon represented the major sources of DOM in the Honnedaga Lake watershed and that photo-biodegradation likely exerted a stronger influence than photodegradation or biodegradation alone in shaping changes in Φ_app,^1^O_2__ and Φ_app,^3^DOM*_ during DOM transit from terrestrial sources through downstream aquatic compartments.

To identify predictors for the variations in Φ_app,^1^O_2__ and Φ_app,^3^DOM*_, redundancy analysis was performed using Φ_app,^1^O_2_,high-energy_, Φ_app,^1^O_2_,low-energy_, Φ_app,^3^DOM_TMP_^*^,high-energy_, Φ_app,^3^DOM_TMP_^*^,low-energy_, and Φ_app,^3^DOM_HDO_^*^_ as a matrix of response variables and a subset of DOM optical and redox properties (i.e., those with a variance inflation factor of <2.5) as a matrix of explanatory variables. Five variables, including FI, SUVA_254_, *S*_290–400_, AOC, and β:α, collectively explained 76.4% of the overall variation in Φ_app,^1^O_2__ and Φ_app,^3^DOM*_ (Figure 4b), among which FI explained the most variation. Of these five variables, SUVA_254_, *S*_290–400_, AOC, and β:α have all been proposed as effective predictors for Φ_app,^1^O_2__ and Φ_app,^3^DOM*_ in previous DOM photochemistry studies.^103,109,122,128^ Stepwise multiple linear regression analysis performed using these five variables further prioritized FI, SUVA_254_, and *S*_290–400_ as the most consistent combination of predictors for Φ_app,^1^O_2__, Φ_app,^3^DOM_TMP_^*^_, and Φ_app,^3^DOM_HDO_^*^_, with the inclusion of AOC and β:α as two additional predictors for improved model fit (adjusted *R*^2^ = 0.848–0.868; Table S17). Overall, hierarchical cluster analysis provided qualitative evidence for the role of photo-biodegradation in regulating DOM photoreactivity in the Honnedaga Lake watershed, while redundancy analysis and multiple linear regression analysis illustrated the extent to which photodegradation- and (photo-)biodegradation-induced changes in DOM character could explain the observed variation in DOM photoreactivity along the watershed terrestrial-aquatic continuum.

### Environmental Implications

This work evaluates the effects of photochemical and microbial processing on Φ_app,RI_ for terrestrial DOM (i.e., extracted from leaf litter and the surface organic-rich horizon) sourced from the Honnedaga Lake watershed in the Adirondack Mountain region of New York that has been undergoing recovery from historical atmospheric acid deposition over recent decades. Our comparative irradiation and incubation experiments highlighted the contrasting impacts of photodegradation and (photo-)biodegradation on Φ_app,RI_ and the relative contribution of high-energy and low-energy ^3^DOM* to Φ_app,^1^O_2__ and Φ_app,^3^DOM*_. Multivariate statistical analyses further revealed the potential relevance of photo-biodegradation and shifts in DOM character (as reflected by the changes in bulk optical and redox properties) for explaining the variations in the magnitude and patterns of Φ_app,^1^O_2__ and Φ_app,^3^DOM*_ along the terrestrial-aquatic continuum of the Honnedaga Lake watershed. Collectively, these results support the paradigm that sunlight, microbes, and their interactions serve as key controls for DOM photoreactivity.^37^

Our study only focused on DOM collected from one lake watershed of regional importance and did not incorporate a multiscale approach to couple laboratory experiments with field-scale measurements or modeling, so results from this work should be interpreted with insights gained from investigations covering a greater spatiotemporal gradient and integrating a more explicit consideration of ecosystem properties. Future work should also explore the links between the community structure and/or physiological processes of microbial assemblages and the molecular composition and structural characteristics of photoreactive DOM moieties^134,136,137^ as well as the drivers for convergence of photoreactivity as terrestrial DOM exported to aquatic environments.^138^ Furthermore, establishing baselines upon which to quantify the cooperative and competitive effects of biogeochemical controls^23^ on DOM photoreactivity would require methodologically consistent studies to resolve uncertainties associated with DOM renewal and turnover at multiple scales.^37^ Closing these knowledge gaps will be essential for defining the role of photochemically and microbially modified terrestrial DOM in aquatic photochemistry processes given the dynamic linkages between increasing terrestrial carbon inputs and browning of inland waters^139−141^ and will eventually contribute to a more holistic understanding of DOM photoreactivity continuum within and across ecosystem boundaries.^142^

## Supporting Information Available

Map of the Honnedaga Lake watershed sampling sites; physicochemical characteristics and DOM properties of samples; photochemistry experimental details and summary of photochemical parameters; changes in Φ_app,^1^O_2__, Φ_app,^3^DOM_TMP_^*^_, and Φ_app,^•^OH_ during photodegradation of SRFA and ESHA; changes in Φ_app,^1^O_2__, Φ_app,^3^DOM_TMP_^*^_, and Φ_app,^3^DOM_HDO_^*^_ during biodegradation of glucose and ESHA; changes in the energy distribution of ^3^DOM* contributing to Φ_app,^1^O_2__ and Φ_app,^3^DOM_TMP_^*^_ during photodegradation and (photo-)biodegradation; and performance statistics of the multiple linear regression models (PDF)
