# Supplementary material for: Contrasting Impacts of Photochemical and Microbial Processing on the Photoreactivity of Dissolved Organic Matter in an Adirondack Lake Watershed
Source: Environ Sci Technol. 2022 Jan 18;56(3):1688–701. doi: 10.1021/acs.est.1c06047 (PMC8812123; doi:10.1021/acs.est.1c06047)
Supplement: Supplementary file 1 — es1c06047_si_001.pdf [file es1c06047_si_001.pdf]

Supporting Information for

Contrasting Impacts of Photochemical and Microbial Processing on the Photoreactivity of Dissolved Organic Matter in an Adirondack Lake Watershed

Joseph Wasswa[1], Charles T. Driscoll[1], Teng Zeng[,1]*

[1]Department of Civil and Environmental Engineering, Syracuse University, 151 Link Hall, Syracuse, New York 13244, United States

*Corresponding Author: Teng Zeng: Email: tezeng@syr.edu; Phone: +1-315-443-1099

(Total 82 pages, 9 texts, 18 tables, 11 figures)

Table of Contents

1. Chemicals, reagents, and glassware S3

2. Map of the Honnedaga Lake watershed sampling sites S5

3. Physicochemical characteristics and DOM properties of Honnedaga samples S6

4. Analytical methods for photochemistry experiments S17

5. *p*-Nitroanisole/pyridine actinometry S18

6. Terephthalic acid (TPA) as a probe for $^{\bullet}OH$ S23

7. Furfuryl alcohol (FFA) as a probe for $^1O_2$ S27

8. 2,4,6-Trimethylphenol (TMP) as an electron transfer probe for $^3DOM^*$ S35

9. *trans,trans*-2,4-Hexadien-1-ol (*t,t*-HDO) as an energy transfer probe for $^3DOM^*$ S45

10. $\Phi_{app,RI}$ profile fittings during irradiation or incubation of leaf and soil Oa DOM S54

11. Changes in $\Phi_{app,\,^1O_2}$, $\Phi_{app,\,^3DOM^*_{TMP}}$, and $\Phi_{app,\,^{\cdot}OH}$ during photodegradation of SRFA and ESHA S57

12. Changes in $\Phi_{app,\,^1O_2}$, $\Phi_{app,\,^3DOM^*_{TMP}}$, and $\Phi_{app,\,^3DOM^*_{HDO}}$ during biodegradation of glucose and ESHAS58

13. Changes in $\Phi_{app,\,^{\cdot}OH}$ during biodegradation and photo-biodegradation of leaf and soil Oa DOM S59

14. Changes in the energy distribution of $^3DOM^*$ contributing to $\Phi_{app,\,^1O_2}$ S60

15. Changes in the energy distribution of $^3DOM^*$ contributing to $\Phi_{app,\,^3DOM^*_{TMP}}$ S61

16. Performance statistics of the multiple linear regression models S62

17. Summary of literature data on the apparent quantum yields of RIs S63

References S75

1. Chemicals, reagents, and glassware

Chemicals and reagents were used as received without further purification unless otherwise noted. Methanol (MeOH; HPLC grade), acetonitrile (ACN; HPLC grade), water (HPLC grade), sodium hydroxide solution (NaOH; 0.1 N certified), sulfuric acid solution (H_2SO_4; 0.1 N certified), hydrochloric acid solution (HCl; 0.1 N certified), *o*-phosphoric acid (H_3PO_4; certified ACS grade), acetic acid (HPLC grade), trifluoroacetic acid (TFA; ≥98.5%), ammonium acetate (≥97%), potassium persulfate (99+%), sodium sulfate (Na_2SO_4; ≥99.0%), and sodium bicarbonate ($NaHCO_3$; 99.7 to 100.3%) were purchased from Fisher Scientific. Sodium chloride (NaCl; 99.5%), sodium carbonate (Na_2CO_3; 99.95%), sodium nitrite ($NaNO_2$; ≥99.0%), sodium nitrate ($NaNO_3$; 99+%), sodium dihydrogen phosphate monohydrate ($NaH_2PO_4 \cdot H_2O$; 99+%), sodium phosphate dibasic heptahydrate ($Na_2HPO_4 \cdot 7H_2O$; 99+%), ammonium hydroxide (NH_4OH; 25% free ammonia in water), terephthalic acid (TPA; 99+%), furfuryl alcohol (FFA; 98%), 2,4,6-trimethylphenol (TMP; 99%), *p*-nitroanisole (PNA; 99+%; recrystallized[1]), and pyridine (pyr; 99+%) were purchased from ACROS Organics. Folin & Ciocalteu's phenol reagent (2N), gallic acid (≥98.0%), 2-hydroxyterephthalic acid (hTPA; 97%), and *trans*,*trans*-2,4-hexadien-1-ol (*t,t*-HDO; sorbic alcohol; 97%), (±)-6-hydroxy-2,5,7,8-tetramethylchromane-2-carboxylic acid (Trolox; 97%), and 2,2'-azino-bis(3-ethylbenzothiazoline-6-sulfonic acid) diammonium salt (≥98%) were purchased from Sigma-Aldrich. Potassium hydrogen phthalate solution (certified carbon standard, 1000 ppm) was purchased from LabChem. pH buffer solutions (pH 4.01, 7.00, and 10.01) were purchased from Mettler Toledo. Suwannee River fulvic acid (SRFA; 3S101F), Suwannee River humic acid (SRHA; 3S101H), Suwannee River natural organic matter (SRNOM; 2R101N), Pahokee Peat fulvic acid (PPFA; 2S103F), Pahokee Peat humic acid (PPHA; 1S103H), Nordic Reservoir natural organic matter (NRNOM; 1R108N), Upper Mississippi River natural organic matter (UMRNOM; 1R110N), and Elliott Soil humic acid (ESHA; 5S102H) were purchased from the International Humic Substance Society (IHSS).

Stock solutions were typically prepared by dissolving or diluting a gravimetrically weighted amount of solid or liquid standards into HPLC grade water. Bimolecular PNA/pyr actinometer solutions (10 μM PNA/5 mM pyr) were prepared freshly on the day of experimentation by mixing 10 mM of *p*-nitroanisole and 12.36 M of pyridine

stock solutions at a predetermined volumetric ratio.[2, 3] Working solutions and calibration standards were prepared by diluting predetermined volumes of stock solutions into ultrapure water (resistivity 18.2 MΩ•cm) generated by a Thermo Scientific Barnstead MicroPure UV/UF water purification system. Stock and working solutions were stored at 4 °C. Mobile phases for HPLC and HPIC analysis were prepared using HPLC grade water and solvents.

Non-volumetric glassware was rinsed 5 times with HPLC grade methanol, followed by 5 times with ultrapure water, and combusted at 450 °C in a Thermo Scientific Lindberg/Blue M Moldatherm box furnace for a minimum of 5 h. Volumetric glassware, quartz vessels, and microsyringes were rinsed with HPLC grade methanol and ultrapure water and dried at 70 °C in a Fisherbrand Isotemp general purpose heating and drying oven.

2. Map of the Honnedaga Lake watershed sampling sites

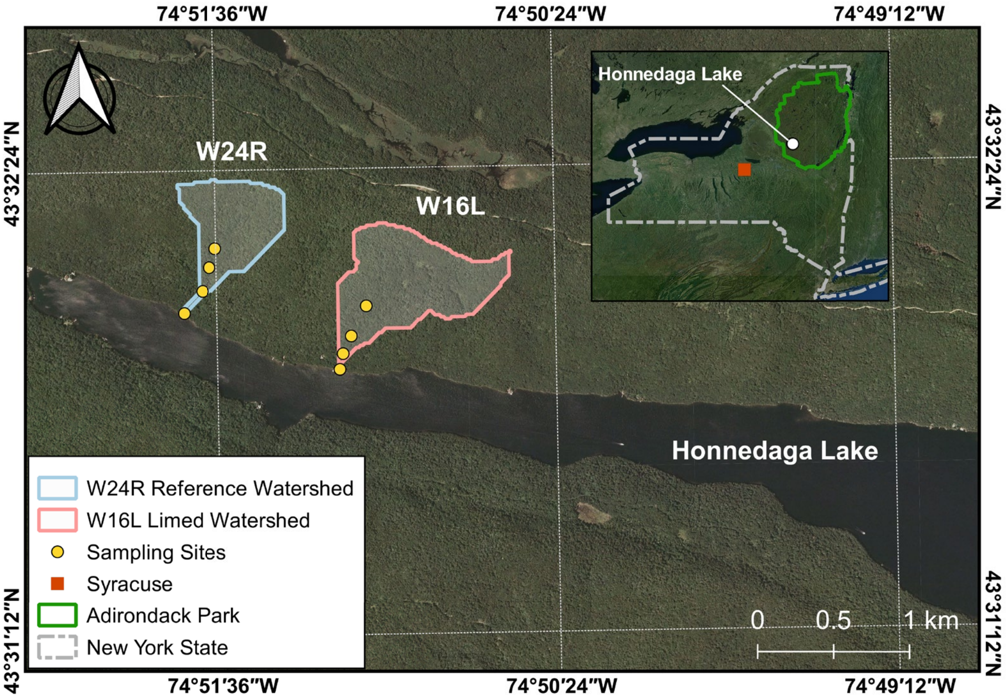

Figure S1. Map of the Honnedaga Lake watershed sampling sites in the Adirondack Mountain region of New York. Within the lake watershed, soils mainly consist of spodosols, and the vegetative species are predominantly American beech (*Fagus grandifolia Ehrh.*), red maple (*Acer rubrum L.*), and yellow birch (*Betula alleghaniensis Britt.*). Satellite Image Source: Esri, Maxar, GeoEye, Earthstar Geographics, CNES/Airbus DS, USDA, USGS, AeroGRID, IGN, and the GIS User Community.

3. Physicochemical characteristics and DOM properties of Honnedaga samples

For each leachate, whole water sample, or model DOM solution, dissolved organic carbon (DOC) was measured by high-temperature catalytic combustion using a Teledyne-Tekmar Torch total organic carbon analyzer (calibrated by potassium hydrogen phthalate solutions). Four anions (chloride, nitrite, nitrate, and sulfate) were measured by a Thermo Scientific Integrion high-pressure ion chromatograph (HPIC). For selected samples, five base cations (sodium, potassium, magnesium, calcium, strontium) and ten trace metals (aluminum, iron, manganese, cobalt, nickel, copper, zinc, chromium, cadmium, lead) were analyzed by a PerkinElmer NexION 2000 inductively coupled plasma mass spectrometer (ICP-MS).

For each standardized leachate and water sample ([DOC] = 4 mg C/L; pH 6.5±0.1), UV-visible absorbance spectra were recorded in a Starna Cells 1-I-10 quartz cuvette (1-cm pathlength) from 200 to 700 nm in 1-nm increments using a Thermo Scientific Evolution 201 UV-visible spectrophotometer and corrected for blank and long-wavelength baseline.[4] Fluorescence excitation-emission matrices (EEMs) were measured in a Starna Cells 3-Q-10 quartz cuvette (1-cm pathlength) using a Horiba Scientific Aqualog spectrofluorometer.[4] Lamp, cuvette, and Raman water scans were checked prior to each analysis following the manufacturer's recommended protocol. EEMs were recorded across an excitation wavelength range of 240 to 550 nm in 2-nm increments and an emission wavelength range of 247.68 to 830.02 nm in 2.33-nm increments with an integration time of 1 s and a medium CCD gain. Prior to data analysis, EEMs were corrected for instrument-specific correction factors[5] and inner filter effects,[6, 7] blank subtracted, and normalized against the Raman peak area of a Starna Cells RM-H_2O Raman water fluorescence reference standard.[8, 9] Optical indices,[10-13] such as $SUVA_{254}$ (the specific UV absorbance at 254 nm),[14] $E2$:$E3$ (the ratio of Napierian absorption coefficients at 250 and 365 nm),[15] spectral slope coefficients (e.g., $S_{275\text{-}295}$, $S_{350\text{-}400}$, $S_{290\text{-}400}$),[16, 17] spectral slope ratio (S_R; the ratio of $S_{275\text{-}295}$ to $S_{350\text{-}400}$),[18] fluorescence index (FI),[19, 20] humification index (HIX),[6, 21, 22] freshness index (β:α),[23-25] and redox index (RIX),[26] were extracted from the absorbance and EEM fluorescence data using *MATLAB*.[4] Parallel factor analysis (PARAFAC) was also performed to deconvolute EEMs (n = 151) using the *drEEM* toolbox (v. 0.6.3)[9] in *MATLAB*. Upon examination of the core consistencies, sum of squared errors, and $S_4C_6T_3$ split-half validations,[9] a 4-component model was identified as

the most appropriate model that explained 99.2% of the measured spectral variation across reverse-normalized EEMs (**Figure S2**). Following the model validation, the true scores were converted to the maximum fluorescence intensity in water Raman unit (R.U.),[9] and the excitation and emission wavelengths of each component were queried through the *OpenFluor* database.[27] Four PARAFAC components were defined as component 1 (C1), component 2 (C2), component 3 (C3), and component 4 (C4), respectively (**Figure S3**). C1 is a terrestrial humic-like component (high aromaticity, high molecular weight),[12, 28] C2 is a microbial humic-like component (aliphatic, low molecular weight),[12, 28] C3 resembles a humic-like component (low aromaticity, low molecular weight),[12, 28-30] and C4 is a tryptophan-like component (aliphatic, low molecular weight).[12, 28, 31]

The antioxidant capacity (AOC) was determined by the 2,2'-azinobis-(3-ethylbenzothiazoline-6-sulfonic acid) (ABTS) assay.[32, 33] Briefly, the $ABTS^{•+}$ solution was generated by reacting 7 mM of 2,2'-azino-bis(3-ethylbenzothiazoline-6-sulfonic acid) diammonium salt with 2.45 mM of potassium persulfate and diluted with HPLC grade water and methanol (50:50 v/v) to an absorbance of 0.70±0.01 at 734 nm. Each sample (0.5 mL) was mixed with $ABTS^{•+}$ solution (2.4 mL), diluted by HPLC grade water (2 mL), thoroughly vortexed, and left to stand in the dark for 6 min. AOC (using Trolox as a redox standard and expressed in mmol e^-/g C)[34] was then determined spectrophotometrically at 734 nm using the Evolution 201 UV-visible spectrophotometer. The total phenolic content ([Phenolic]) was measured by the Folin-Ciocalteu assay.[35, 36] Briefly, each sample (0.5 mL) was mixed with the Folin-Ciocalteu reagent (0.2 mL), diluted by HPLC grade water (2 mL), thoroughly vortexed, and left to stand in the dark for 5 min. The mixture was then amended with Na_2CO_3 (0.6 mL of 20% w/v solution) and incubated at 45 °C in a Fisherbrand Isotemp water bath for 30 min. [Phenolic] (expressed in gallic acid equivalents) was then determined spectrophotometrically at 765 nm using the Evolution 201 UV-visible spectrophotometer. Note that the Folin-Ciocalteu assay does not necessarily capture the entire pool of phenolic moieties in DOM and should be complemented by other methods (e.g., alkaline CuO oxidation followed by gas chromatography-mass spectrometry[37] or flow-injection analysis with electrochemical detection[34]) when possible.

Major physicochemical characteristics and DOM properties of Honnedaga leachates and whole water samples are summarized in **Tables S1-S4**.

Table S1. Concentrations of dissolved organic carbon in leachates, whole water samples, and model DOM solutions

Sample Name	[DOC] (mg C/L)	Sample Name	[DOC] (mg C/L)	Sample Name	[DOC] (mg C/L)
Soil Oa L Photo 0 h	12.01±0.10	Soil Oa R Photo 0 h	12.03±0.06	Leaf Photo 0 h	12.04±0.11
Soil Oa L Photo 1 h	12.33±0.15	Soil Oa R Photo 1 h	11.94±0.02	Leaf Photo 1 h	12.88±0.44
Soil Oa L Photo 2 h	12.35±0.31	Soil Oa R Photo 2 h	11.98±0.10	Leaf Photo 2 h	13.11±0.12
Soil Oa L Photo 4 h	10.37±0.13	Soil Oa R Photo 4 h	11.02±0.13	Leaf Photo 4 h	11.84±0.33
Soil Oa L Photo 8 h	10.34±0.03	Soil Oa R Photo 8 h	10.53±0.31	Leaf Photo 8 h	11.63±0.18
Soil Oa L Photo 16 h	9.90±0.33	Soil Oa R Photo 16 h	10.31±0.26	Leaf Photo 16 h	11.48±0.61
Soil Oa L Photo 32 h	9.49±0.05	Soil Oa R Photo 32 h	8.54±0.09	Leaf Photo 32 h	10.87±0.09
Soil Oa L Photo 64 h	8.26±0.02	Soil Oa R Photo 64 h	7.24±0.05	Leaf Photo 64 h	9.81±0.16
Soil Oa L Photo 96 h	7.38±0.30	Soil Oa R Photo 96 h	4.74±0.11	Leaf Photo 96 h	9.22±0.10
Soil Oa L Bio 0 d	16.61±0.09	Soil Oa R Bio 0 d	17.27±0.09	Leaf Bio 0 d	19.49±0.16
Soil Oa L Bio 8 d	16.27±0.51	Soil Oa R Bio 8 d	16.92±0.04	Leaf Bio 8 d	18.40±0.13
Soil Oa L Bio 16 d	16.26±0.01	Soil Oa R Bio 16 d	16.92±0.30	Leaf Bio 16 d	17.57±0.04
Soil Oa L Bio 32 d	16.75±0.30	Soil Oa R Bio 32 d	17.57±0.55	Leaf Bio 32 d	17.22±0.37
Soil Oa L Photo-Bio 0 d	16.47±0.05	Soil Oa R Photo-Bio 0 d	16.72±0.05	Leaf Photo-Bio 0 d	20.22±0.66
Soil Oa L Photo-Bio 8 d	15.81±0.65	Soil Oa R Photo-Bio 8 d	15.59±0.26	Leaf Photo-Bio 8 d	17.48±0.14
Soil Oa L Photo-Bio 16 d	15.74±0.20	Soil Oa R Photo-Bio 16 d	15.69±0.05	Leaf Photo-Bio 16 d	16.75±0.09
Soil Oa L Photo-Bio 32 d	16.10±0.94	Soil Oa R Photo-Bio 32 d	15.78±0.32	Leaf Photo-Bio 32 d	15.76±0.09
SRFA Photo 0 h	14.82±0.58	Glucose Bio 0 d	15.03±0.85	Stream L	6.13±0.05
SRFA Photo 4 h	14.61±0.52	Glucose Bio 8 d	2.24±0.20	Stream R	5.05±0.06
SRFA Photo 16 h	14.15±0.28	Glucose Bio 16 d	2.01±0.25	Honnedaga Lake	1.75±0.09
SRFA Photo 64 h	13.53±0.31	Glucose Bio 32 d	1.97±0.24		
ESHA Photo 0 h	16.05±0.11	ESHA Bio 0 d	15.17±0.25		
ESHA Photo 4 h	15.79±0.36	ESHA Bio 8 d	14.84±0.01		
ESHA Photo 16 h	15.74±0.07	ESHA Bio 16 d	14.71±0.09		
ESHA Photo 64 h	15.40±0.10	ESHA Bio 32 d	14.70±0.25		

"Photo" = photodegradation; "Bio" = biodegradation; "Photo-Bio" = photo-biodegradation; "L" = the limed tributary watershed W16L; "R" = the reference tributary watershed W24R. The limit of detection for [DOC] was 0.02 mg C/L.

**Table S2.** Concentrations of anions in leachates and whole water samples				
Sample Name	[Cl^-] (μM)	[NO_2^-] (μM)	[NO_3^-] (μM)	[SO_4^{2-}] (μM)
Soil Oa L Photo 0 h	15.0±0.2	<0.5	36.0±1.4	5.3±0.1
Soil Oa L Photo 1 h	20.4±7.2	0.5±0.1	39.9±2.1	6.0±0.2
Soil Oa L Photo 2 h	38.2±1.5	0.5±0.2	44.1±0.1	6.9±0.1
Soil Oa L Photo 4 h	41.0±1.4	0.7±0.1	48.6±5.3	8.4±0.5
Soil Oa L Photo 8 h	46.5±0.1	0.8±0.1	49.3±4.8	8.6±0.3
Soil Oa L Photo 16 h	51.8±3.7	0.9±0.1	52.1±4.5	9.0±0.1
Soil Oa L Photo 32 h	70.1±8.7	1.1±0.1	55.9±2.6	9.6±0.3
Soil Oa L Photo 64 h	79.2±3.8	1.2±0.2	61.0±0.4	11.0±0.1
Soil Oa L Photo 96 h	103.7±10.5	2.2±0.1	68.0±0.3	17.0±1.6
Soil Oa L Bio 0 d	19.9±1.7	1.7±0.3	37.7±0.7	9.6±1.2
Soil Oa L Bio 8 d	18.3±0.8	1.1±0.2	220.8±0.8	6.8±0.1
Soil Oa L Bio 16 d	17.8±1.0	0.7±0.3	211.0±7.0	6.5±0.2
Soil Oa L Bio 32 d	15.8±0.5	0.5±0.3	201.2±4.6	6.5±0.2
Soil Oa L Photo-Bio 0 d	42.1±1.5	1.0±0.4	34.5±8.2	9.9±1.3
Soil Oa L Photo-Bio 8 d	20.7±3.7	0.6±0.1	220.4±5.4	7.7±1.1
Soil Oa L Photo-Bio 16 d	18.6±1.4	<0.5	206.8±6.8	6.9±0.1
Soil Oa L Photo-Bio 32 d	14.9±2.8	<0.5	199.6±12.8	6.3±0.3
Sample Name	[Cl^-] (μM)	[NO_2^-] (μM)	[NO_3^-] (μM)	[SO_4^{2-}] (μM)
Soil Oa R Photo 0 h	16.8±5.3	<0.5	23.3±0.1	3.9±0.1
Soil Oa R Photo 1 h	40.2±3.5	0.6±0.1	23.8±0.4	4.1±0.1
Soil Oa R Photo 2 h	41.3±3.6	0.7±0.1	26.5±0.8	4.6±0.2
Soil Oa R Photo 4 h	46.8±5.4	0.8±0.2	27.1±1.2	5.9±0.8
Soil Oa R Photo 8 h	53.3±1.0	0.8±0.1	31.6±1.9	6.4±0.7
Soil Oa R Photo 16 h	56.4±0.8	1.1±0.1	33.4±0.1	6.7±0.6
Soil Oa R Photo 32 h	78.5±0.1	1.3±0.1	34.6±0.1	7.1±0.3
Soil Oa R Photo 64 h	82.3±1.0	1.4±0.2	40.1±0.1	7.3±0.1
Soil Oa R Photo 96 h	88.0±0.2	2.6±0.2	62.8±2.5	11.4±0.5
Soil Oa R Bio 0 d	42.1±1.5	1.0±0.4	34.5±8.2	9.9±1.3
Soil Oa R Bio 8 d	20.7±3.7	0.6±0.1	208.8±0.1	7.7±1.1
Soil Oa R Bio 16 d	18.6±1.4	<0.5	202.4±1.5	6.9±0.1
Soil Oa R Bio 32 d	14.9±2.8	<0.5	190.8±1.8	6.3±0.3
Soil Oa R Photo-Bio 0 d	44.3±0.6	1.4±0.1	23.0±0.2	6.6±1.1
Soil Oa R Photo-Bio 8 d	30.9±0.3	1.1±0.1	215.4±9.3	5.0±0.8
Soil Oa R Photo-Bio 16 d	14.1±0.2	0.6±0.1	203.4±2.7	4.1±0.1
Soil Oa R Photo-Bio 32 d	13.0±0.8	<0.5	197.2±5.1	4.1±0.1

"Photo" = photodegradation; "Bio" = biodegradation; "Photo-Bio" = photo-biodegradation; "L" = the limed tributary watershed W16L; "R" = the reference tributary watershed W24R. The HPIC limit of detection for [Cl^-], [NO_2^-], [NO_3^-], and [SO_4^{2-}] was 1.1 μM, 0.5 μM, 0.6 μM, and 0.4 μM, respectively. Elevated [NO_3^-] in biodegraded and photo-biodegraded soil Oa leachate samples were due to the initial nutrient amendment to relieve possible nutrient limitation.

**Table S2.** Concentrations of anions in leachates and whole water samples (continued)				
Sample Name	[Cl^-] (μM)	[NO_2^-] (μM)	[NO_3^-] (μM)	[SO_4^{2-}] (μM)
Leaf Photo 0 h	14.2±3.6	0.5±0.1	<0.6	1.8±0.3
Leaf Photo 1 h	33.0±6.1	0.6±0.2	0.6±0.1	2.0±0.1
Leaf Photo 2 h	35.9±6.9	0.7±0.2	0.6±0.1	2.2±0.2
Leaf Photo 4 h	46.3±1.4	0.9±0.1	0.7±0.1	2.3±0.2
Leaf Photo 8 h	52.0±3.8	1.0±0.2	0.8±0.2	2.4±0.1
Leaf Photo 16 h	60.1±11.3	1.1±0.1	0.9±0.3	2.7±0.1
Leaf Photo 32 h	80.2±2.6	1.2±0.1	1.2±0.4	2.9±0.1
Leaf Photo 64 h	91.5±1.1	1.4±0.1	2.3±0.3	3.4±0.1
Leaf Photo 96 h	102.2±6.6	2.5±0.5	3.2±0.8	9.6±2.8
Leaf Bio 0 d	10.5±1.3	0.9±0.2	1.1±0.1	1.7±0.3
Leaf Bio 8 d	9.0±1.1	0.8±0.2	230.1±4.6	1.5±0.1
Leaf Bio 16 d	8.0±0.2	0.5±0.1	201.7±3.8	1.3±0.1
Leaf Bio 32 d	7.9±0.1	<0.5	184.8±4.9	1.3±0.1
Leaf Photo-Bio 0 d	39.8±0.3	1.7±0.8	0.9±0.3	2.1±0.3
Leaf Photo-Bio 8 d	10.4±2.1	1.0±0.1	217.9±0.3	1.8±0.3
Leaf Photo-Bio 16 d	9.7±1.4	0.7±0.1	194.3±3.6	1.5±0.1
Leaf Photo-Bio 32 d	8.3±0.4	<0.5	181.5±3.9	1.4±0.1
Sample Name	[Cl^-] (μM)	[NO_2^-] (μM)	[NO_3^-] (μM)	[SO_4^{2-}] (μM)
Soil Oa L-L	16.0±0.8	0.6±0.1	29.6±0.1	3.0±0.2
Soil Bs L-L	7.6±0.1	0.8±0.4	31.6±0.1	4.2±0.1
Soil Oa L-M	17.0±0.1	0.8±0.1	36.1±5.3	3.5±0.1
Soil Bs L-M	8.6±0.2	1.1±0.4	29.8±3.7	5.8±0.1
Soil Oa L-H	16.0±0.1	0.8±0.1	32.3±0.1	2.6±0.1
Soil Bs L-H	5.5±0.1	0.8±0.3	31.6±0.1	3.5±0.1
Soil Oa R-L	14.8±0.1	1.5±0.2	16.3±0.1	2.2±0.1
Soil Bs R-L	3.7±0.1	1.5±0.3	15.1±0.1	2.3±0.1
Soil Oa R-M	16.8±0.1	0.6±0.1	17.5±0.1	3.9±0.1
Soil Bs R-M	5.4±0.1	1.2±0.1	19.0±0.3	2.7±0.1
Soil Oa R-H	15.6±0.7	0.9±0.1	21.0±0.8	2.8±0.6
Soil Bs R-H	4.0±0.6	1.0±0.2	20.5±8.5	4.8±0.3
Stream L	17.6±0.6	0.8±0.3	20.8±0.2	17.9±0.2
Stream R	11.5±1.3	0.6±0.1	11.3±0.7	30.4±0.7
Honnedaga Lake	16.5±0.1	1.2±0.1	23.9±0.7	31.3±3.5

"Photo" = photodegradation; "Bio" = biodegradation; "Photo-Bio" = photo-biodegradation; "L" = the limed tributary watershed W16L; "R" = the reference tributary watershed W24R; "L-L" = the low elevation site in the limed tributary watershed; "L-M" = the medium elevation site in the limed tributary watershed; "L-H" = the high elevation site in the limed tributary watershed; "R-L" = the low elevation site in the reference tributary watershed; "R-M" = the medium elevation site in the reference tributary watershed; "R-H" = the high elevation site in the reference tributary watershed. The HPIC limit of detection for [Cl^-], [NO_2^-], [NO_3^-], and [SO_4^{2-}] was 1.1 μM, 0.5 μM, 0.6 μM, and 0.4 μM, respectively. Elevated [NO_3^-] in biodegraded and photo-biodegraded leaf leachate samples were due to the initial nutrient amendment to relieve possible nutrient limitation.

Table S3. Concentrations of base cations and trace metals in selected leachates and whole water samples

Sample Name	[Na^+] (μM)	[Mg^{2+}] (μM)	[Al] (μM)	[K^+] (μM)	[Ca^{2+}] (μM)	[Mn] (μM)	[Fe] (μM)	[Co] (μM)	[Ni] (μM)	[Cu] (μM)	[Zn] (μM)	[Mo] (μM)	[Cd] (μM)	[Pb] (μM)
Soil Oa L Photo 0 h	89.192	3.469	9.453	69.008	7.773	0.021	0.267	0.001	0.004	0.054	0.437	0.068	<0.001	0.003
Soil Oa L Photo 16 h	96.420	3.350	6.382	31.957	9.651	0.021	0.221	0.001	0.005	0.069	0.502	0.006	<0.001	0.003
Soil Oa R Photo 0 h	65.470	1.809	8.525	74.319	4.326	0.091	0.640	0.001	0.003	0.066	0.359	0.022	0.001	0.004
Soil Oa R Photo16 h	68.418	1.912	7.812	61.467	4.854	0.106	0.641	0.001	0.004	0.068	0.407	0.006	0.001	0.003
Leaf Photo 0 h	42.446	1.188	3.461	81.907	4.311	0.207	<0.001	0.001	0.002	0.041	0.141	0.008	<0.001	0.001
Leaf Photo 16 h	48.024	1.579	3.554	87.398	4.895	0.234	0.024	0.001	0.002	0.076	0.137	0.005	<0.001	0.001
Soil Oa L-L	26.810	1.611	16.808	6.807	4.858	0.045	0.057	0.001	0.010	0.035	0.108	0.010	<0.001	0.001
Soil Bs L-L	36.626	2.472	15.693	5.716	4.964	0.080	0.339	0.003	0.010	0.049	0.180	0.008	<0.001	0.001
Soil Oa L-M	31.100	1.470	20.997	8.727	4.526	0.036	0.061	0.001	0.007	0.048	0.066	0.008	<0.001	0.001
Soil Bs L-M	25.301	2.667	11.932	4.361	4.483	0.066	0.230	0.001	0.005	0.042	0.158	0.010	<0.001	0.001
Soil Oa L-H	24.228	1.743	7.568	6.906	7.517	0.031	0.068	0.001	0.005	0.019	0.063	0.011	<0.001	0.001
Soil Bs L-H	21.475	2.463	8.907	2.955	5.536	0.089	0.312	0.001	0.005	0.017	0.098	0.008	<0.001	0.001
Soil Oa R-L	20.986	1.103	6.011	3.723	4.197	0.014	0.073	<0.001	0.005	0.035	0.057	0.007	<0.001	<0.001
Soil Bs R-L	22.394	1.442	11.698	2.795	5.043	0.019	0.477	0.001	0.004	0.023	0.126	0.007	<0.001	<0.001
Soil Oa R-M	21.964	2.091	10.173	9.514	3.712	0.029	0.087	0.001	0.005	0.017	0.077	0.007	<0.001	<0.001
Soil Bs R-M	24.834	3.004	11.557	3.610	3.871	0.026	0.532	0.001	0.008	0.072	0.118	0.007	<0.001	0.001
Soil Oa R-H	34.358	2.573	19.887	19.962	4.541	0.072	0.074	0.001	0.053	0.039	0.343	0.007	<0.001	0.005
Soil Bs R-H	26.646	3.745	13.849	6.422	3.857	0.092	0.488	0.002	0.021	0.041	0.341	0.007	<0.001	0.001
Stream L	34.445	4.850	26.213	4.641	20.696	0.092	0.390	0.002	0.006	0.101	0.133	0.007	<0.001	0.002
Stream R	34.025	7.819	20.607	5.303	20.053	0.192	0.386	0.004	0.008	0.054	4.498	0.006	0.001	0.001
Honnedaga Lake	56.341	9.730	6.906	3.737	31.198	0.265	0.177	<0.001	0.007	0.027	0.064	<0.001	<0.001	<0.001

"Photo" = photodegradation; "L" = the limed tributary watershed W16L; "R" = the reference tributary watershed W24R; "L-L" = the low elevation site in the limed tributary watershed; "L-M" = the medium elevation site in the limed tributary watershed; "L-H" = the high elevation site in the limed tributary watershed; "R-L" = the low elevation site in the reference tributary watershed; "R-M" = the medium elevation site in the reference tributary watershed; "R-H" = the high elevation site in the reference tributary watershed. The ICP-MS limit of detection for base cations and trace metals was 0.001 μM. Only selected samples were analyzed for base cations and trace metals due to limited sample volumes.

**Table S4.** Optical and redox properties of standardized leachates and whole water samples												
Sample Name	*E2:E3*	$S_{275–295}$ (μm^{-1})	$S_{350–400}$ (μm^{-1})	$S_{290–400}$ (μm^{-1})	S_R	$SUVA_{254}$ (L mg C^{-1}•m^{-1})	FI	HIX	$\beta:\alpha$	RIX	[Phenolic] (g gallic acid/g C)	AOC (mmol e^-/g C)
Soil Oa L Photo 0 h	4.16±0.03	12.1±0.1	15.0±1.2	13.9±0.8	0.81±0.06	4.68±0.08	1.38±0.02	0.97±0.01	0.33±0.01	0.72±0.01	0.32±0.01	2.38±0.25
Soil Oa L Photo 1 h	4.21±0.09	12.8±0.3	15.9±0.8	14.6±0.1	0.80±0.02	4.54±0.10	1.36±0.01	0.96±0.01	0.35±0.01	0.69±0.01	0.31±0.09	2.27±0.25
Soil Oa L Photo 2 h	4.29±0.01	13.0±0.1	16.4±0.2	14.8±0.1	0.80±0.01	4.37±0.22	1.34±0.02	0.96±0.01	0.36±0.01	0.69±0.01	0.29±0.03	2.21±0.16
Soil Oa L Photo 4 h	4.38±0.04	13.5±0.1	16.6±0.3	14.8±0.1	0.82±0.02	4.23±0.35	1.34±0.02	0.95±0.01	0.37±0.01	0.68±0.01	0.28±0.05	2.15±0.25
Soil Oa L Photo 8 h	4.44±0.02	13.9±0.1	16.7±0.2	14.9±0.2	0.83±0.01	3.89±0.02	1.32±0.01	0.95±0.01	0.37±0.01	0.66±0.01	0.24±0.03	2.09±0.49
Soil Oa L Photo 16 h	4.75±0.08	15.0±0.2	16.9±0.4	15.4±0.3	0.89±0.01	3.83±0.02	1.31±0.01	0.95±0.01	0.39±0.02	0.63±0.02	0.23±0.02	2.03±0.08
Soil Oa L Photo 32 h	5.04±0.19	16.3±0.1	17.0±0.5	15.6±0.6	0.96±0.02	3.74±0.04	1.31±0.02	0.93±0.01	0.40±0.01	0.60±0.01	0.22±0.02	1.97±0.33
Soil Oa L Photo 64 h	5.49±0.14	18.0±0.3	17.2±0.3	15.6±0.6	1.05±0.01	3.63±0.04	1.29±0.02	0.93±0.01	0.42±0.01	0.55±0.01	0.20±0.04	1.92±0.08
Soil Oa L Photo 96 h	5.64±0.17	18.9±0.6	17.7±0.2	16.1±0.1	1.06±0.02	3.24±0.13	1.26±0.01	0.90±0.01	0.47±0.01	0.54±0.01	0.16±0.04	1.80±0.08
Soil Oa L Bio 0 d	4.14±0.02	12.1±0.1	16.3±0.1	15.4±0.1	0.74±0.01	4.78±0.04	1.38±0.02	0.96±0.01	0.33±0.01	0.71±0.01	0.34±0.03	2.15±0.08
Soil Oa L Bio 8 d	4.11±0.01	8.0±0.1	16.3±0.1	15.4±0.1	0.49±0.01	4.84±0.01	1.40±0.03	0.98±0.01	0.34±0.01	0.71±0.01	0.36±0.06	4.71±0.58
Soil Oa L Bio 16 d	4.06±0.05	7.9±0.1	16.2±0.1	15.2±0.2	0.49±0.01	4.96±0.01	1.41±0.03	0.98±0.01	0.35±0.02	0.72±0.01	0.42±0.02	6.74±0.16
Soil Oa L Bio 32 d	3.71±0.02	7.9±0.1	15.8±0.1	14.3±0.1	0.50±0.01	5.11±0.02	1.41±0.03	0.99±0.01	0.36±0.02	0.73±0.01	0.44±0.05	7.90±0.99
Soil Oa L Photo-Bio 0 d	4.36±0.07	11.1±2.5	16.6±1.4	15.7±0.6	0.68±0.21	4.60±0.06	1.33±0.01	0.95±0.01	0.35±0.03	0.68±0.01	0.31±0.03	2.27±0.25
Soil Oa L Photo-Bio 8 d	4.27±0.09	8.9±0.4	15.7±0.1	15.3±0.1	0.57±0.02	4.79±0.10	1.35±0.01	0.97±0.01	0.36±0.02	0.69±0.01	0.40±0.04	6.80±0.25
Soil Oa L Photo-Bio 16 d	4.16±0.01	8.5±0.1	15.6±0.1	15.2±0.1	0.55±0.01	4.88±0.02	1.36±0.01	0.98±0.01	0.37±0.02	0.69±0.01	0.42±0.04	7.61±0.08
Soil Oa L Photo-Bio 32 d	3.93±0.22	8.5±0.1	15.3±0.1	14.5±0.6	0.55±0.01	5.25±0.28	1.38±0.02	0.99±0.01	0.38±0.01	0.70±0.01	0.44±0.08	9.12±1.23

"Photo" = photodegradation; "Bio" = biodegradation; "Photo-Bio" = photo-biodegradation; "L" = the limed tributary watershed W16L; "R" = the reference tributary watershed W24R; "L-L" = the low elevation site in the limed tributary watershed; "L-M" = the medium elevation site in the limed tributary watershed; "L-H" = the high elevation site in the limed tributary watershed; "R-L" = the low elevation site in the reference tributary watershed; "R-M" = the medium elevation site in the reference tributary watershed; "R-H" = the high elevation site in the reference tributary watershed; *E2:E3* = the ratio of absorption coefficients at 250 and 365 nm;[15] $S_{275–295}$ = the spectral slope coefficient from 275 to 295 nm;[17] $S_{350–400}$ = the spectral slope coefficient from 350 to 400 nm;[17] $S_{290–400}$ = the spectral slope coefficient from 290 to 400 nm;[16] S_R = the ratio of $S_{275-295}$ to $S_{350-400}$;[18] $SUVA_{254}$ = specific UV absorbance at 254 nm;[14] FI = fluorescence index;[19, 20] HIX = humification index;[6, 21, 22] $\beta:\alpha$ = freshness index;[23-25] RIX = redox index (note that the original abbreviation for redox index is "RI", but "RIX" is used herein to distinguish between reactive intermediates and redox index);[26] [Phenolic] = the total phenolic content in gallic acid equivalents; AOC = the antioxidant capacity in mmol e^- g^{-1}C.

**Table S4.** Optical and redox properties of standardized leachates and whole water samples (continued)												
Sample Name	*E2*:*E3*	$S_{275–295}$ (μm^{-1})	$S_{350–400}$ (μm^{-1})	$S_{290–400}$ (μm^{-1})	S_R	SUVA$_{254}$ (L mg C^{-1}•m^{-1})	FI	HIX	β:α	RIX	[Phenolic] (g gallic acid/g C)	AOC (mmol e$^-$/g C)
Soil Oa R Photo 0 h	4.41±0.02	12.7±0.2	15.2±1.0	14.3±1.1	0.84±0.07	4.47±0.06	1.41±0.01	0.98±0.01	0.32±0.01	0.72±0.01	0.34±0.02	2.73±0.25
Soil Oa R Photo 1 h	4.48±0.03	13.4±0.1	16.9±0.2	15.0±0.1	0.79±0.02	4.33±0.01	1.40±0.01	0.98±0.01	0.33±0.01	0.69±0.01	0.32±0.03	2.67±0.33
Soil Oa R Photo 2 h	4.51±0.05	13.8±0.1	17.1±0.2	15.3±0.1	0.81±0.02	4.18±0.07	1.40±0.01	0.97±0.01	0.35±0.01	0.67±0.01	0.31±0.01	2.61±0.41
Soil Oa R Photo 4 h	4.58±0.05	14.3±0.2	17.4±0.1	15.6±0.2	0.82±0.01	4.07±0.09	1.38±0.01	0.97±0.01	0.36±0.01	0.66±0.01	0.27±0.03	2.56±0.33
Soil Oa R Photo 8 h	4.60±0.04	14.4±0.4	17.6±0.3	15.8±0.1	0.82±0.01	4.04±0.06	1.37±0.01	0.96±0.01	0.37±0.02	0.62±0.03	0.24±0.04	2.50±0.41
Soil Oa R Photo 16 h	5.23±0.02	16.2±0.5	17.9±0.6	16.0±0.1	0.91±0.06	3.82±0.09	1.35±0.02	0.96±0.01	0.40±0.01	0.58±0.01	0.22±0.03	2.44±0.66
Soil Oa R Photo 32 h	5.41±0.09	17.6±0.2	18.4±0.1	16.1±0.1	0.96±0.01	3.66±0.26	1.33±0.02	0.94±0.01	0.48±0.02	0.58±0.01	0.19±0.02	2.38±0.41
Soil Oa R Photo 64 h	6.10±0.01	19.5±0.1	18.5±0.1	16.2±0.2	1.05±0.01	3.29±0.12	1.31±0.02	0.93±0.01	0.52±0.01	0.53±0.01	0.16±0.01	2.32±0.16
Soil Oa R Photo 96 h	6.18±0.07	20.2±0.1	18.9±0.3	16.6±0.4	1.07±0.02	3.01±0.03	1.30±0.01	0.88±0.01	0.55±0.01	0.51±0.03	0.15±0.01	2.15±0.08
Soil Oa R Bio 0 d	4.49±0.01	12.7±0.1	18.1±0.1	16.5±0.1	0.70±0.01	4.71±0.03	1.41±0.01	0.97±0.01	0.32±0.01	0.71±0.01	0.33±0.08	2.61±0.25
Soil Oa R Bio 8 d	4.48±0.01	8.7±0.1	18.0±0.2	16.4±0.1	0.48±0.01	4.75±0.01	1.41±0.02	0.98±0.01	0.32±0.02	0.72±0.01	0.36±0.04	4.47±0.41
Soil Oa R Bio 16 d	4.41±0.02	8.5±0.1	17.9±0.2	16.2±0.1	0.48±0.01	4.81±0.02	1.43±0.01	0.99±0.01	0.33±0.01	0.72±0.01	0.39±0.07	6.10±0.58
Soil Oa R Bio 32 d	4.00±0.01	8.5±0.1	17.5±0.2	15.4±0.1	0.49±0.01	5.14±0.05	1.45±0.01	0.99±0.01	0.35±0.01	0.74±0.01	0.41±0.03	6.85±0.16
Soil Oa R Photo-Bio 0 d	4.56±0.04	13.5±0.1	17.1±0.1	16.1±0.1	0.79±0.01	4.80±0.01	1.40±0.01	0.96±0.01	0.34±0.01	0.67±0.01	0.32±0.02	2.67±0.16
Soil Oa R Photo-Bio 8 d	4.51±0.06	9.6±0.2	17.0±0.1	16.1±0.1	0.57±0.01	4.83±0.02	1.40±0.01	0.97±0.01	0.34±0.02	0.67±0.01	0.40±0.02	6.33±0.25
Soil Oa R Photo-Bio 16 d	4.48±0.04	9.2±0.1	16.8±0.1	16.0±0.1	0.55±0.01	4.97±0.10	1.41±0.01	0.98±0.01	0.36±0.01	0.67±0.01	0.41±0.05	7.84±0.74
Soil Oa R Photo-Bio 32 d	4.08±0.07	9.2±0.1	16.5±0.1	14.8±0.1	0.56±0.01	5.28±0.04	1.43±0.01	0.98±0.01	0.36±0.01	0.68±0.01	0.43±0.04	9.24±0.08

"Photo" = photodegradation; "Bio" = biodegradation; "Photo-Bio" = photo-biodegradation; "L" = the limed tributary watershed W16L; "R" = the reference tributary watershed W24R; "L-L" = the low elevation site in the limed tributary watershed; "L-M" = the medium elevation site in the limed tributary watershed; "L-H" = the high elevation site in the limed tributary watershed; "R-L" = the low elevation site in the reference tributary watershed; "R-M" = the medium elevation site in the reference tributary watershed; "R-H" = the high elevation site in the reference tributary watershed; *E2:E3* = the ratio of absorption coefficients at 250 and 365 nm;[15] $S_{275–295}$ = the spectral slope coefficient from 275 to 295 nm;[17] $S_{350–400}$ = the spectral slope coefficient from 350 to 400 nm;[17] $S_{290–400}$ = the spectral slope coefficient from 290 to 400 nm;[16] S_R = the ratio of $S_{275-295}$ to $S_{350-400}$;[18] SUVA$_{254}$ = specific UV absorbance at 254 nm;[14] FI = fluorescence index;[19, 20] HIX = humification index;[6, 21, 22] β:α = freshness index;[23-25] RIX = redox index (note that the original abbreviation for redox index is "RI", but "RIX" is used herein to distinguish between reactive intermediates and redox index);[26] [Phenolic] = the total phenolic content in gallic acid equivalents; AOC = the antioxidant capacity in mmol e$^-$ g^{-1}C.

**Table S4.** Optical and redox properties of standardized leachates and whole water samples (continued)												
Sample Name	*E2*:*E3*	$S_{275–295}$ (μm^{-1})	$S_{350–400}$ (μm^{-1})	$S_{290–400}$ (μm^{-1})	S_R	$SUVA_{254}$ (L mg C^{-1}•m^{-1})	FI	HIX	*β:α*	RIX	[Phenolic] (g gallic acid/g C)	AOC (mmol e^-/g C)
Leaf Photo 0 h	4.25±0.03	12.7±0.4	18.0±1.5	15.5±0.7	0.71±0.08	3.43±0.26	1.53±0.01	0.96±0.01	0.31±0.01	0.69±0.01	0.37±0.01	3.31±0.08
Leaf Photo 1 h	4.44±0.01	13.6±0.6	18.4±1.1	15.7±0.7	0.74±0.07	3.15±0.03	1.50±0.02	0.95±0.01	0.33±0.01	0.66±0.01	0.34±0.03	3.25±0.16
Leaf Photo 2 h	4.54±0.01	14.3±0.1	19.5±1.1	16.6±0.7	0.73±0.04	3.07±0.04	1.47±0.01	0.94±0.01	0.34±0.01	0.65±0.02	0.31±0.05	3.19±0.25
Leaf Photo 4 h	4.63±0.01	14.6±0.4	19.9±1.3	16.8±0.7	0.73±0.07	3.02±0.10	1.43±0.02	0.94±0.01	0.34±0.01	0.64±0.03	0.29±0.07	3.14±0.49
Leaf Photo 8 h	4.80±0.21	15.2±0.1	20.4±0.8	17.1±0.7	0.74±0.03	2.98±0.14	1.38±0.01	0.92±0.01	0.35±0.01	0.62±0.01	0.28±0.03	3.08±0.08
Leaf Photo 16 h	5.06±0.46	16.4±0.6	20.7±0.8	17.4±0.7	0.79±0.01	2.76±0.02	1.31±0.03	0.89±0.01	0.35±0.01	0.59±0.01	0.26±0.06	3.02±0.33
Leaf Photo 32 h	6.09±0.10	19.0±0.3	22.5±1.7	18.9±0.9	0.84±0.08	2.66±0.10	1.28±0.02	0.87±0.01	0.36±0.01	0.56±0.01	0.24±0.05	2.96±0.25
Leaf Photo 64 h	7.39±0.19	22.3±0.1	25.4±1.5	21.1±0.8	0.88±0.05	2.40±0.03	1.25±0.05	0.86±0.01	0.37±0.01	0.52±0.01	0.22±0.04	2.85±0.08
Leaf Photo 96 h	8.24±0.38	23.9±0.1	28.0±1.0	22.6±0.8	0.85±0.04	1.97±0.15	1.19±0.02	0.84±0.01	0.37±0.01	0.51±0.01	0.17±0.04	2.61±0.08
Leaf Bio 0 d	4.23±0.03	13.8±0.4	17.3±0.1	15.8±0.1	0.80±0.02	3.57±0.22	1.53±0.01	0.96±0.01	0.32±0.01	0.68±0.01	0.43±0.06	3.25±0.16
Leaf Bio 8 d	4.19±0.01	7.8±0.1	16.6±0.1	15.6±0.1	0.47±0.01	3.75±0.01	1.61±0.02	0.98±0.01	0.35±0.03	0.68±0.01	0.47±0.03	5.63±0.25
Leaf Bio 16 d	4.15±0.05	7.8±0.1	16.3±0.1	15.5±0.1	0.48±0.01	4.02±0.04	1.65±0.02	0.98±0.01	0.35±0.03	0.70±0.01	0.48±0.01	7.61±0.74
Leaf Bio 32 d	3.99±0.02	7.7±0.1	15.6±0.7	15.0±0.3	0.50±0.02	4.58±0.10	1.67±0.01	0.98±0.01	0.36±0.02	0.72±0.01	0.49±0.01	9.00±0.90
Leaf Photo-Bio 0 d	4.57±0.02	14.5±0.1	16.8±0.1	15.7±0.1	0.86±0.01	3.39±0.11	1.46±0.01	0.94±0.01	0.34±0.01	0.64±0.01	0.34±0.02	3.08±0.25
Leaf Photo-Bio 8 d	4.49±0.02	9.0±0.1	16.2±0.1	15.6±0.1	0.56±0.01	3.74±0.14	1.57±0.01	0.96±0.01	0.36±0.03	0.65±0.01	0.49±0.07	7.38±0.25
Leaf Photo-Bio 16 d	4.44±0.03	8.8±0.1	16.0±0.2	15.5±0.2	0.55±0.01	4.02±0.06	1.58±0.01	0.97±0.01	0.37±0.03	0.65±0.01	0.51±0.03	8.71±0.33
Leaf Photo-Bio 32 d	4.41±0.01	8.9±0.3	15.9±0.3	15.3±0.1	0.56±0.01	4.39±0.01	1.61±0.02	0.97±0.01	0.38±0.02	0.66±0.01	0.53±0.04	9.41±0.16

"Photo" = photodegradation; "Bio" = biodegradation; "Photo-Bio" = photo-biodegradation; "L" = the limed tributary watershed W16L; "R" = the reference tributary watershed W24R; "L-L" = the low elevation site in the limed tributary watershed; "L-M" = the medium elevation site in the limed tributary watershed; "L-H" = the high elevation site in the limed tributary watershed; "R-L" = the low elevation site in the reference tributary watershed; "R-M" = the medium elevation site in the reference tributary watershed; "R-H" = the high elevation site in the reference tributary watershed; *E2:E3* = the ratio of absorption coefficients at 250 and 365 nm;[15] $S_{275–295}$ = the spectral slope coefficient from 275 to 295 nm;[17] $S_{350–400}$ = the spectral slope coefficient from 350 to 400 nm;[17] $S_{290–400}$ = the spectral slope coefficient from 290 to 400 nm;[16] S_R = the ratio of $S_{275-295}$ to $S_{350-400}$;[18] $SUVA_{254}$ = specific UV absorbance at 254 nm;[14] FI = fluorescence index;[19, 20] HIX = humification index;[6, 21, 22] *β:α* = freshness index;[23-25] RIX = redox index (note that the original abbreviation for redox index is "RI", but "RIX" is used herein to distinguish between reactive intermediates and redox index);[26] [Phenolic] = the total phenolic content in gallic acid equivalents; AOC = the antioxidant capacity in mmol e^- $g^{-1}C$.

**Table S4.** Optical and redox properties of standardized leachates and whole water samples (continued)												
Sample Name	*E2*:*E3*	$S_{275–295}$ (μm^{-1})	$S_{350–400}$ (μm^{-1})	$S_{290–400}$ (μm^{-1})	S_R	$SUVA_{254}$ (L mg C^{-1}•m^{-1})	FI	HIX	*β*:*α*	RIX	[Phenolic] (g gallic acid/g C)	AOC (mmol e^-/g C)
Soil Oa L-L	4.15±0.01	12.5±0.3	15.2±1.0	14.7±0.1	0.82±0.04	4.74±0.03	1.37±0.02	0.97±0.01	0.33±0.01	0.71±0.01	0.36±0.04	2.48±0.40
Soil Bs L-L	2.85±0.08	8.8±0.1	14.6±0.7	14.4±0.1	0.61±0.03	6.39±0.44	1.64±0.02	0.98±0.01	0.39±0.01	0.74±0.01	0.30±0.08	1.35±0.08
Soil Oa L-M	4.13±0.01	12.8±0.3	14.9±0.4	14.9±0.1	0.86±0.01	4.69±0.18	1.37±0.02	0.96±0.01	0.33±0.01	0.71±0.01	0.37±0.04	2.20±0.16
Soil Bs L-M	2.83±0.15	8.9±0.5	14.2±0.6	14.5±0.1	0.63±0.01	6.35±0.08	1.65±0.01	0.98±0.01	0.39±0.01	0.74±0.01	0.31±0.05	1.24±0.24
Soil Oa L-H	4.19±0.01	12.9±0.2	15.3±0.9	15.0±0.5	0.85±0.04	4.82±0.05	1.38±0.01	0.97±0.01	0.33±0.01	0.71±0.01	0.35±0.05	2.25±0.56
Soil Bs L-H	2.86±0.11	9.1±0.1	14.5±1.1	14.7±0.8	0.63±0.04	6.37±0.14	1.67±0.01	0.98±0.01	0.39±0.01	0.73±0.01	0.29±0.08	1.30±0.16
Soil Oa R-L	4.46±0.05	13.0±0.7	16.5±0.2	14.5±1.3	0.79±0.03	4.59±0.04	1.41±0.01	0.98±0.01	0.32±0.01	0.71±0.01	0.37±0.04	2.65±0.16
Soil Bs R-L	2.88±0.03	8.8±0.1	14.9±0.3	13.9±1.0	0.59±0.01	6.45±0.06	1.64±0.02	0.99±0.01	0.38±0.01	0.73±0.01	0.30±0.06	1.75±0.32
Soil Oa R-M	4.45±0.05	13.0±0.3	16.6±0.1	14.8±1.3	0.78±0.02	4.52±0.02	1.42±0.01	0.98±0.01	0.32±0.01	0.71±0.01	0.37±0.04	3.04±0.40
Soil Bs R-M	2.82±0.01	8.9±0.1	15.1±0.7	14.0±1.1	0.59±0.03	6.15±0.08	1.68±0.01	0.98±0.01	0.38±0.01	0.73±0.01	0.28±0.07	1.97±0.32
Soil Oa R-H	4.46±0.01	12.6±0.2	16.1±0.1	14.8±0.7	0.78±0.01	4.43±0.24	1.42±0.01	0.98±0.01	0.32±0.01	0.71±0.01	0.37±0.05	2.76±0.16
Soil Bs R-H	2.75±0.05	8.7±0.2	15.2±0.1	14.1±1.0	0.57±0.02	6.11±0.06	1.70±0.01	0.98±0.01	0.38±0.01	0.73±0.02	0.29±0.07	1.69±0.24
Stream L	5.28±0.01	15.4±0.6	19.8±0.4	17.2±0.3	0.78±0.05	3.41±0.01	1.65±0.03	0.94±0.01	0.47±0.01	0.66±0.01	0.31±0.03	1.35±0.24
Stream R	6.00±0.14	17.3±0.2	16.8±0.5	16.3±0.2	1.03±0.04	3.90±0.04	1.63±0.06	0.86±0.01	0.53±0.01	0.62±0.01	0.29±0.05	1.52±0.32
Honnedaga Lake	6.55±0.02	19.7±0.1	21.6±0.7	18.9±0.4	0.92±0.03	1.22±0.03	1.59±0.03	0.90±0.01	0.51±0.01	0.57±0.01	0.33±0.03	1.24±0.24

"Photo" = photodegradation; "Bio" = biodegradation; "Photo-Bio" = photo-biodegradation; "L" = the limed tributary watershed W16L; "R" = the reference tributary watershed W24R; "L-L" = the low elevation site in the limed tributary watershed; "L-M" = the medium elevation site in the limed tributary watershed; "L-H" = the high elevation site in the limed tributary watershed; "R-L" = the low elevation site in the reference tributary watershed; "R-M" = the medium elevation site in the reference tributary watershed; "R-H" = the high elevation site in the reference tributary watershed; *E2:E3* = the ratio of absorption coefficients at 250 and 365 nm;[15] $S_{275–295}$ = the spectral slope coefficient from 275 to 295 nm;[17] $S_{350–400}$ = the spectral slope coefficient from 350 to 400 nm;[17] $S_{290–400}$ = the spectral slope coefficient from 290 to 400 nm;[16] S_R = the ratio of $S_{275-295}$ to $S_{350-400}$;[18] $SUVA_{254}$ = specific UV absorbance at 254 nm;[14] FI = fluorescence index;[19, 20] HIX = humification index;[6, 21, 22] *β*:*α* = freshness index;[23-25] RIX = redox index (note that the original abbreviation for redox index is "RI", but "RIX" is used herein to distinguish between reactive intermediates and redox index);[26] [Phenolic] = the total phenolic content in gallic acid equivalents; AOC = the antioxidant capacity in mmol e^- g^{-1}C.

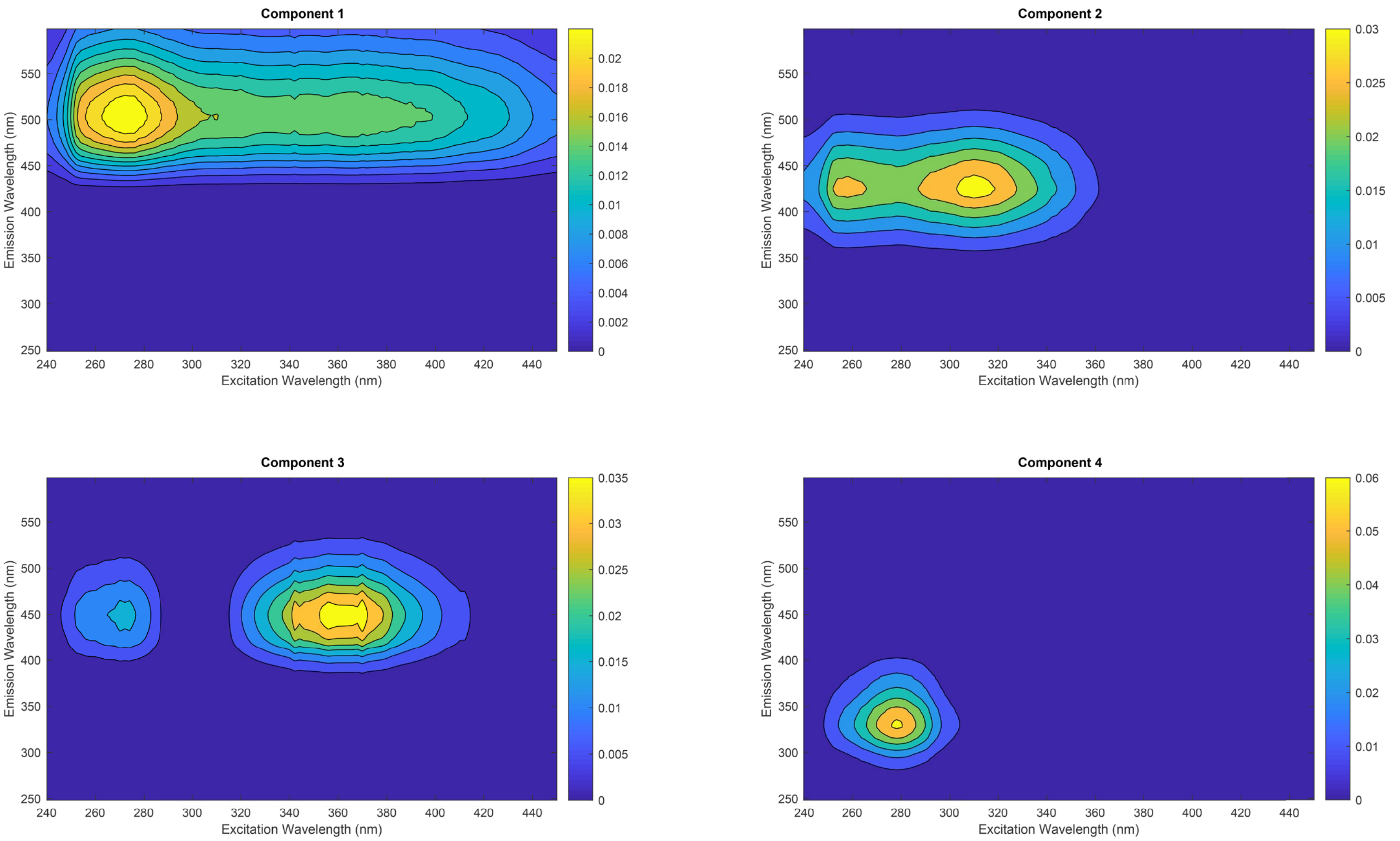

Figure S2. Contour plots of 4 fluorescent components validated for the 4-component PARAFAC model.

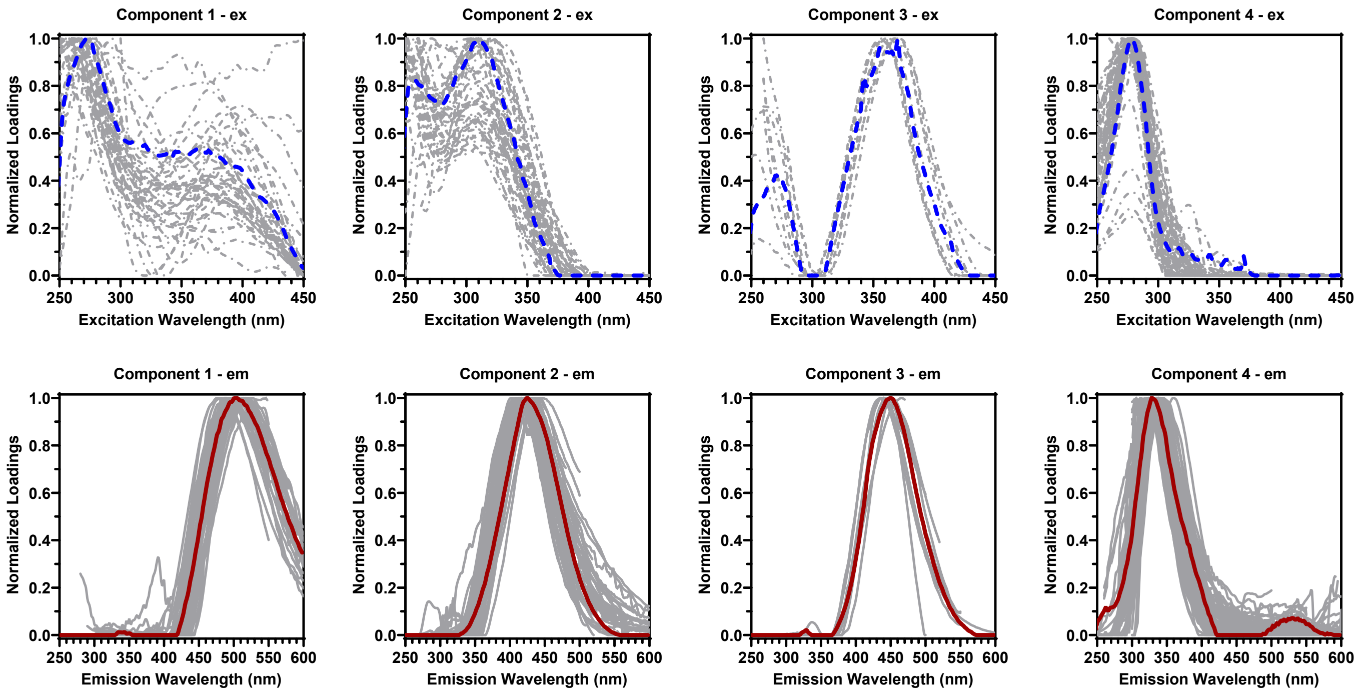

Figure S3. Spectral comparisons of the 4-component PARAFAC model with those published in the *OpenFluor* database.

4. Analytical methods for photochemistry experiments

Over the course of irradiation, 500 μL of sample aliquots were withdrawn from quartz test tubes at predetermined time intervals and analyzed for the concentrations of 2-hydroxyterephthalic acid (hTPA), furfuryl alcohol (FFA), 2,4,6-trimethylphenol (TMP), four sorbic alcohol isomers (i.e., *cis*,*cis*-2,4-hexadien-1-ol, *cis*,*trans*-2,4-hexadien-1-ol, *trans*,*cis*-2,4-hexadien-1-ol, *trans*,*trans*-2,4-hexadien-1-ol), and *p*-nitroanisole (PNA) by an Agilent 1260 Infinity II high-performance liquid chromatograph with a variable wavelength detector and a fluorescence detector with instrument configurations and settings detailed in **Table S5**. The formation of hTPA from terephthalic acid (TPA),[89, 90] the loss of FFA,[38, 39] the loss of TMP,[40, 41] and the isomerization of *t,t*-HDO[42] were monitored as a function of time following the methods established in our previous work.[4]

Table S5. High-performance liquid chromatography methods

Analyte	Mobile Phase	Analytical Column	Detector	Retention Time
PNA	25% Water<br>75% MeOH<br>Isocratic flow rate: 0.5 mL/min	Agilent Poroshell 120 EC-C18<br>4.6 × 100 mm, 2.7 μm<br>Column temperature: 30 °C	VWD absorbance<br>λ_{UV} = 316 nm	3.2 min
hTPA	75% 10 mM Phosphate buffer<br>25% MeOH<br>Isocratic flow rate: 0.5 mL/min	Agilent Poroshell 120 EC-C18<br>4.6 × 100 mm, 2.7 μm<br>Column temperature: 30 °C	FLD fluorescence<br>$\lambda_{excitation}$ = 250 nm<br>$\lambda_{emission}$ = 410 nm	3.6 min
FFA	80% 10 mM Phosphate buffer<br>20% MeOH<br>Isocratic flow rate: 0.5 mL/min	Agilent Poroshell 120 EC-C18<br>4.6 × 100 mm, 2.7 μm<br>Column temperature: 30 °C	VWD absorbance<br>λ_{UV} = 217 nm	4.2 min
TMP	30% 10 mM Phosphate buffer<br>70% MeOH<br>Isocratic flow rate: 0.5 mL/min	Agilent Poroshell 120 EC-C18<br>4.6 × 100 mm, 2.7 μm<br>Column temperature: 30 °C	FLD fluorescence<br>$\lambda_{excitation}$ = 230 nm<br>$\lambda_{emission}$ = 325 nm	4. 6 min
t,t-HDO	70% Water with 0.05% TFA<br>30% ACN<br>Isocratic flow rate: 0.7 mL/min	Phenomenex Luna C18<br>4.6 × 250 mm, 5 μm<br>Column temperature: 10 °C	VWD absorbance<br>λ_{UV} = 230 nm	*c,c*-HDO 13.6 min<br>*c,t*-HDO 14.1 min<br>*t,c*-HDO 14.6 min<br>*t,t*-HDO 15.3 min

VWD = variable wavelength detection; FLD = fluorescence detection; *c,c*-HDO = *cis,cis*-2,4-hexadien-1-ol; *c,t*-HDO = *cis,trans*-2,4-hexadien-1-ol; *t,c*-HDO = *trans,cis*-2,4-hexadien-1-ol; *t,t*-HDO = *trans,trans*-2,4-hexadien-1-ol. The limit of detection for PNA, hTPA, FFA, TMP, and *t,t*-HDO was 0.04 μM, 0.5 nM, 0.06 μM, 0.01 μM, and 0.02 μM, respectively.

5. *p*-Nitroanisole/pyridine actinometry

For each standardized sample ([DOC] = 4 mg C/L; pH 6.5±0.1), the rate of light absorption R_a (mol-photons L^{-1} s^{-1} or Einstein L^{-1} s^{-1}) per unit sample volume was calculated as described in our previous work.[4] For each set of photochemistry experiments, the loss of PNA in 10 μM PNA/5 mM pyr actinometer solutions was monitored in the solar simulator to determine the pseudo-first order rate constant for the loss of PNA, $k_{obs,PNA}$ (s^{-1}):[2, 43, 44]

$$R_{loss,\,PNA} = -\frac{d[PNA]}{dt} = k_{obs,\,PNA}[PNA] = \Phi_{PNA}\sum_{\lambda\,=\,290\,nm}^{550\,nm}\frac{W_\lambda \varepsilon_\lambda[1-10^{-\alpha_{D(\lambda)}z}]}{z\alpha_\lambda}[PNA]$$
$$\approx \Phi_{PNA}\sum_{\lambda\,=\,290\,nm}^{550\,nm} I_\lambda \frac{\varepsilon_\lambda[1-10^{-\alpha_\lambda z}]}{\alpha_\lambda}[PNA] \qquad (S1)$$

where $R_{loss,\,PNA}$ (M s^{-1}) is the loss rate of PNA, [PNA] is the concentration of PNA, Φ_{PNA} (1.74×10^{-3} mol mol-photons^{-1} or mol Einstein^{-1}; calculated from Φ_{PNA} = 0.29 [pyr] + 0.00029) is the quantum yield for the loss of PNA at a given pyridine concentration (i.e., [pyr] = 5×10^{-3} M),[3] W_λ (10^{-3} mol-photons cm^{-2} s^{-1} nm^{-1} or milliEinstein cm^{-2} s^{-1} nm^{-1}) is the incident light intensity at a given wavelength λ, ε_λ (M^{-1} cm^{-1}) is the decadic molar absorption coefficient of PNA at a given wavelength λ,[3] $\alpha_{D(\lambda)}$ (cm^{-1}) is the apparent (or diffuse) attenuation coefficient ($\alpha_{D(\lambda)} \approx \alpha_\lambda$ where the distribution function D(λ) is ~1.0 for the quartz test tube [44]), z (1.12 cm) is the optical pathlength for the quartz test tube,[43] α_λ (cm^{-1}) is the decadic absorption (or attenuation) coefficient (i.e., the absorbance divided by the optical pathlength for the quartz cuvette), and I_λ (10^{-3} mol-photons cm^{-3} s^{-1} nm^{-1} or milliEinstein cm^{-3} s^{-1} nm^{-1} or mol-photons L^{-1} s^{-1} nm^{-1}) is the incident light intensity at a given wavelength λ per unit volume. On average, R_a for Honnedaga samples integrated over the wavelength range of 290-550 nm (chosen based on the wavelength dependence of $\Phi_{app,RI}$ [45, 46]) accounted for 96.7±3.3% of R_a integrated over the wavelength range of 290-700 nm (**Figure S4(A)**).

Equation S1 was re-written given that I_λ can be approximated by multiplying the fractional spectral intensity of the xenon arc lamp, ρ_λ (nm^{-1}), with the total incident light intensity from 290 to 550 nm per unit volume, I_0 (mol-photons L^{-1} s^{-1}; the wavelength range was selected to account for PNA absorbance past 400 nm[47]):[3, 48, 49]

$$k_{\text{obs, PNA}} = 2.303z\Phi_{\text{PNA}} \sum_{\lambda\,=\,290\text{ nm}}^{550\text{ nm}} \rho_\lambda I_0 \varepsilon_\lambda = 2.303z\Phi_{\text{PNA}} I_0 \sum_{\lambda\,=\,290\text{ nm}}^{550\text{ nm}} \rho_\lambda \varepsilon_\lambda \tag{S2}$$

Equation S2 was further re-arranged to calculate I_0, assuming that I_0 measured by the PNA/pyr actinometer represented I_0 through a given sample:[3, 48, 49]

$$I_0 = \frac{k_{\text{obs, PNA}}}{2.303z\Phi_{\text{PNA}} \sum_{\lambda\,=\,290\text{ nm}}^{550\text{ nm}} \rho_\lambda \varepsilon_\lambda} \tag{S3}$$

Equation S3 was used to calculated R_a using the sample-specific α_λ (m^{-1}):[48, 49]

$$R_a = \sum_{\lambda\,=\,290\text{ nm}}^{550\text{ nm}} \frac{W_\lambda(1 - 10^{-\alpha_{D,\lambda} z})}{z} \approx \sum_{\lambda\,=\,290\text{ nm}}^{550\text{ nm}} \rho_\lambda I_0 \,(1 - 10^{-\alpha_\lambda z}) \tag{S4}$$

For each standardized sample, R_a (corrected for the contribution of residual NO_3^-) summarized in **Table S6** was used to determine the apparent quantum yields of RIs. Note that the contribution of residual NO_3^- to R_a for standardized samples was minimal (<2%; calculated based on the sample-specific [NO_3^-] and the decadic molar absorption coefficient of NO_3^- measured at a given wavelength λ).

Table S6. Summary of R_a for standardized leachate, surface water, and IHSS samples			
Sample Name	R_a ($\times 10^{-6}$ mol-photons L^{-1} s^{-1})	Sample Name	R_a ($\times 10^{-6}$ mol-photons L^{-1} s^{-1})
Soil Oa L Photo 0 h	5.15±0.11	Soil Oa L Bio 0 d	5.50±0.05
Soil Oa L Photo 1 h	4.96±0.13	Soil Oa L Bio 8 d	5.61±0.08
Soil Oa L Photo 2 h	4.86±0.13	Soil Oa L Bio 16 d	5.85±0.09
Soil Oa L Photo 4 h	4.50±0.02	Soil Oa L Bio 32 d	6.24±0.02
Soil Oa L Photo 8 h	4.25±0.36	Soil Oa L Photo-Bio 0 d	5.26±0.29
Soil Oa L Photo 16 h	3.85±0.50	Soil Oa L Photo-Bio 8 d	5.51±0.26
Soil Oa L Photo 32 h	3.44±0.49	Soil Oa L Photo-Bio 16 d	5.85±0.18
Soil Oa L Photo 64 h	2.66±0.33	Soil Oa L Photo-Bio 32 d	5.94±0.27
Soil Oa L Photo 96 h	2.67±0.39		
Sample Name	R_a ($\times 10^{-6}$ mol-photons L^{-1} s^{-1})	Sample Name	R_a ($\times 10^{-6}$ mol-photons L^{-1} s^{-1})
Soil Oa R Photo 0 h	5.03±0.19	Soil Oa R Bio 0 d	4.64±0.06
Soil Oa R Photo 1 h	4.28±0.29	Soil Oa R Bio 8 d	4.78±0.13
Soil Oa R Photo 2 h	3.96±0.02	Soil Oa R Bio 16 d	4.85±0.07
Soil Oa R Photo 4 h	3.79±0.01	Soil Oa R Bio 32 d	5.25±0.13
Soil Oa R Photo 8 h	3.52±0.13	Soil Oa R Photo-Bio 0 d	4.89±0.04
Soil Oa R Photo 16 h	3.29±0.12	Soil Oa R Photo-Bio 8 d	5.08±0.14
Soil Oa R Photo 32 h	2.99±0.12	Soil Oa R Photo-Bio 16 d	5.22±0.11
Soil Oa R Photo 64 h	2.54±0.15	Soil Oa R Photo-Bio 32 d	5.41±0.03
Soil Oa R Photo 96 h	2.13±0.08		
Sample Name	R_a ($\times 10^{-6}$ mol-photons L^{-1} s^{-1})	Sample Name	R_a ($\times 10^{-6}$ mol-photons L^{-1} s^{-1})
Leaf Photo 0 h	3.44±0.61	Leaf Bio 0 d	3.76±0.14
Leaf Photo 1 h	2.83±0.35	Leaf Bio 8 d	4.05±0.02
Leaf Photo 2 h	2.51±0.25	Leaf Bio 16 d	4.39±0.27
Leaf Photo 4 h	2.42±0.34	Leaf Bio 32 d	5.52±0.36
Leaf Photo 8 h	2.22±0.07	Leaf Photo-Bio 0 d	3.55±0.17
Leaf Photo 16 h	2.23±0.23	Leaf Photo-Bio 8 d	4.04±0.19
Leaf Photo 32 h	1.50±0.03	Leaf Photo-Bio 16 d	4.46±0.14
Leaf Photo 64 h	1.04±0.07	Leaf Photo-Bio 32 d	4.70±0.06
Leaf Photo 96 h	0.75±0.01		

"Photo" = photodegradation; "Bio" = biodegradation; "Photo-Bio" = photo-biodegradation; "L" = the limed tributary watershed W16L; "R" = the reference tributary watershed W24R. Errors represent one standard deviation from duplicate measurements.

**Table S6.** Summary of R_a for standardized leachate, surface water, and IHSS samples (continued)			
Sample Name	R_a ($\times 10^{-6}$ mol-photons L^{-1} s^{-1})	Sample Name	R_a ($\times 10^{-6}$ mol-photons L^{-1} s^{-1})
Soil Oa L-L	5.04±0.11	SRFA	2.53±0.06
Soil Bs L-L	1.39±0.03	SRHA	3.73±0.23
Soil Oa L-M	5.02±0.05	SRNOM	2.45±0.05
Soil Bs L-M	1.23±0.01	PPFA	3.53±0.19
Soil Oa L-H	5.09±0.28	PPHA	4.17±0.09
Soil Bs L-H	1.30±0.07	NRNOM	2.86±0.11
Soil Oa R-L	4.84±0.18	UMRNOM	2.46±0.05
Soil Bs R-L	1.76±0.06	ESHA	4.75±0.08
Soil Oa R-M	4.78±0.06		
Soil Bs R-M	1.48±0.02		
Soil Oa R-H	4.93±0.04		
Soil Bs R-H	1.60±0.01		
Stream L	2.91±0.34		
Stream R	2.04±0.24		
Honnedaga Lake	1.07±0.12		
Sample Name	R_a ($\times 10^{-6}$ mol-photons L^{-1} s^{-1})	Sample Name	R_a ($\times 10^{-6}$ mol-photons L^{-1} s^{-1})
SRFA Photo 0 h	2.53±0.01	Glucose Bio 0 d	0.21±0.01
SRFA Photo 4 h	2.15±0.01	Glucose Bio 8 d	1.58±0.11
SRFA Photo 16 h	1.69±0.04	Glucose Bio 16 d	1.68±0.13
SRFA Photo 64 h	1.22±0.03	Glucose Bio 32 d	2.19±0.26
ESHA Photo 0 h	4.75±0.08	ESHA Bio 0 d	4.75±0.08
ESHA Photo 4 h	4.22±0.09	ESHA Bio 8 d	4.72±0.18
ESHA Photo 16 h	3.64±0.01	ESHA Bio 16 d	4.74±0.13
ESHA Photo 64 h	3.53±0.01	ESHA Bio 32 d	4.87±0.17

"Photo" = photodegradation; "Bio" = biodegradation; "Photo-Bio" = photo-biodegradation; "L" = the limed tributary watershed W16L; "R" = the reference tributary watershed W24R. Errors represent one standard deviation from duplicate measurements.

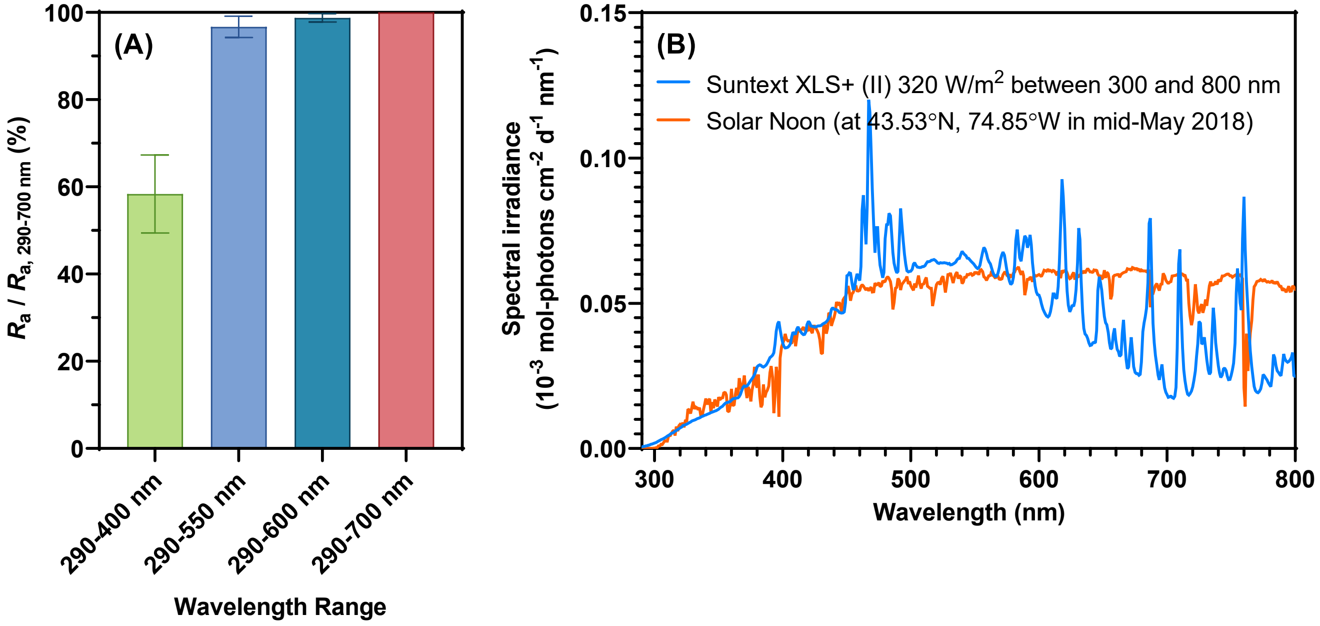

Figure S4. (A) The percentages of R_a integrated over a specific wavelength range with reference to R_a integrated over the wavelength range of 290-700 nm. Error bars indicate the standard deviation of R_a measured for standardized Honnedaga leachates and whole water samples (n=66). **(B)** Comparison of the xenon arc lamp spectral irradiance produced by the Suntest XLS+(II) solar simulator (controlled at 320 W/m^2 between 300 and 800 nm) and the noontime solar spectral irradiance modeled by the Simple Model for Atmospheric Transmission of Sunshine (SMARTS)[84, 85] for mid-May 2018 (i.e., field sampling) at 43.53º N, 74.85º W (i.e., the latitude and longitude coordinates for the Honnedaga Lake watershed).

6. Terephthalic acid (TPA) as a probe for $^{\bullet}OH$

TPA was spiked into samples to measure the photoproduction of $^{\bullet}OH$, recognizing that other lower-energy hydroxylating species[50-53] might also contribute to the observed TPA loss. For each standardized sample, the *net* formation of TPA hydroxylation product, hTPA, was monitored by considering the production of hTPA from TPA and the concurrent loss of hTPA via direct photolysis and its negligible reactions with $^{\bullet}OH$ and $^{1}O_2$:[54, 55]

$$R_{\mathrm{f,\,hTPA}} = \frac{\mathrm{d[hTPA]}}{\mathrm{dt}} = R^{\mathrm{corr}}_{\mathrm{prod,\,hTPA}} - R_{\mathrm{loss,\,hTPA}}$$

$$= k_{\mathrm{TPA},\,^{\bullet}\mathrm{OH}}[\mathrm{TPA}][^{\bullet}\mathrm{OH}]_{\mathrm{ss}}Y_{\mathrm{hTPA}} - R^{\mathrm{NO_3^-}}_{\mathrm{prod,\,hTPA}} - k_{\mathrm{direct\ photolysis,\,hTPA}}[\mathrm{hTPA}]SF_{\Sigma\lambda} - k_{\mathrm{hTPA},\,^{\bullet}\mathrm{OH}}[\mathrm{hTPA}][^{\bullet}\mathrm{OH}]_{\mathrm{ss}} - k_{\mathrm{hTPA},\,^{1}\mathrm{O_2}}[\mathrm{hTPA}][^{1}\mathrm{O_2}]_{\mathrm{ss}} \qquad \text{(S5)}$$

$$\approx k_{\mathrm{TPA},\,^{\bullet}\mathrm{OH}}[\mathrm{TPA}][^{\bullet}\mathrm{OH}]_{\mathrm{ss}}Y_{\mathrm{hTPA}} - k_{\mathrm{direct\ photolysis,\,hTPA}}[\mathrm{hTPA}]SF_{\Sigma\lambda}$$

where $R_{\mathrm{f,\,hTPA}}$ (M s^{-1}) is the formation rate of hTPA, $R^{\mathrm{corr}}_{\mathrm{prod,\,hTPA}}$ (M s^{-1}) is the production rate of hTPA from TPA corrected for that attributable to $^{\bullet}OH$ formed via NO_3^- photolysis (i.e., $R^{\mathrm{NO_3^-}}_{\mathrm{prod,\,hTPA}}$), $R_{\mathrm{loss,\,hTPA}}$ (M s^{-1}) is the loss rate of hTPA, $k_{\mathrm{TPA},\,^{\bullet}\mathrm{OH}}$ (4.2±(0.3)×10^9 M^{-1} s^{-1}) is the second-order reaction rate constant of TPA with $^{\bullet}OH$,[54, 56] [TPA] is the initial concentration of TPA (10 μM), $[^{\bullet}OH]_{ss}$ is the steady-state concentration of $^{\bullet}OH$, Y_{hTPA} is the formation yield hTPA from the reaction of TPA with $^{\bullet}OH$, $k_{\mathrm{direct\ photolysis,\,hTPA}}$ (s^{-1}) is the experimentally determined direct photolysis rate constant of hTPA (measured in ultrapure water spiked with an initial hTPA concentration of 100 nM), [hTPA] is the concentration of hTPA, $SF_{\Sigma\lambda}$ is the sample-specific light screening factor,[57] $k_{\mathrm{hTPA},\,^{\bullet}\mathrm{OH}}$ (6.3±(0.1)×10^9 M^{-1} s^{-1}) is the second-order reaction rate constant of hTPA with $^{\bullet}OH$,[54] $k_{\mathrm{hTPA},\,^{1}\mathrm{O_2}}$ (5.0±(0.1)×10^4 M^{-1} s^{-1}) is the second-order reaction rate constant of hTPA with $^{1}O_2$,[54] and $[^{1}O_2]_{ss}$ is the steady-state concentration of $^{1}O_2$.

For each standardized sample, a nonlinear least squares regression of hTPA data in the initial rate kinetics regime was performed using Equation S5 (i.e., $dx/dt = a + bx$ where dx/dt = d[hTPA]/dt and x = [hTPA]) to solve for the formation rate of $^{\bullet}OH$, $R_{\mathrm{f},\,^{\bullet}\mathrm{OH}}$ (M s^{-1}):[55, 58]

$$R_{\mathrm{f},\,^{\bullet}\mathrm{OH}} = \frac{R_{\mathrm{prod,\,hTPA}}^{\mathrm{corr}}}{Y_{\mathrm{hTPA}}} \quad \text{(S6)}$$

To account for the effects of pH and temperature on $R_{\mathrm{f,\,hTPA}}$, a pH- and temperature-adjusted Y_{hTPA} of 0.37±0.01 was derived by averaging the values predicted from $Y_{\mathrm{hTPA}} = [(30 + 0.43 \times \mathrm{pH}) / 100]_{293\ \mathrm{K}} \times [(0.0059 \pm 0.0011) \times T - (1.50 \pm 0.31)] / [(0.0059 \pm 0.0011) \times 293\ \mathrm{K} - (1.50 \pm 0.31)]$ using T = 298 K (i.e., the chamber temperature of solar simulator was controlled at 25±1 °C throughout the irradiation period) and the average pH of leachates and whole water samples was 6.5±0.1.[54, 56, 59-61]

The steady-state concentration of $^{\bullet}$OH in the presence of TPA, $[^{\bullet}\mathrm{OH}]_{\mathrm{ss}}$, was calculated as:[48, 55]

$$[^{\bullet}\mathrm{OH}]_{\mathrm{ss}} = \frac{R_{\mathrm{f},\,^{\bullet}\mathrm{OH}}}{k'_{\mathrm{q},\,^{\bullet}\mathrm{OH}}} = \frac{R_{\mathrm{prod,\,hTPA}}^{\mathrm{corr}}}{(k_{^{\bullet}\mathrm{OH,\,HCO_3^-}}[\mathrm{HCO_3^-}] + k_{^{\bullet}\mathrm{OH,\,CO_3^{2-}}}[\mathrm{CO_3^{2-}}] + k_{^{\bullet}\mathrm{OH,\,DOM}}[\mathrm{DOM}] + k_{\mathrm{TPA},\,^{\bullet}\mathrm{OH}}[\mathrm{TPA}])Y_{\mathrm{hTPA}}}$$
$$\approx \frac{R_{\mathrm{prod,\,hTPA}}^{\mathrm{corr}}}{(k_{^{\bullet}\mathrm{OH,\,DOM}}[\mathrm{DOM}] + k_{\mathrm{TPA},\,^{\bullet}\mathrm{OH}}[\mathrm{TPA}])Y_{\mathrm{hTPA}}} \quad \text{(S7)}$$

where $k'_{\mathrm{q},\,^{\bullet}\mathrm{OH}}$ (s^{-1}) is the pseudo-first order rate constant for $^{\bullet}$OH quenching, $k_{^{\bullet}\mathrm{OH,\,HCO_3^-}}$ (8.5×10^{6} M^{-1} s^{-1}) is the second-order reaction rate constant of $^{\bullet}$OH with HCO_3^-,[62] [HCO_3^-] is the concentration of HCO_3^-, $k_{^{\bullet}\mathrm{OH,\,CO_3^{2-}}}$ (3.9×10^{8} M^{-1} s^{-1}) is the second-order reaction rate constant of $^{\bullet}$OH with CO_3^{2-},[62] [CO_3^{2-}] is the concentration of CO_3^{2-}, $k_{^{\bullet}\mathrm{OH,\,DOM}}$ (2.9(±1.5)×10^{4} (mg C/L)$^{-1}$ s^{-1} or 3.4(±1.8)×10^{8} (mol C/L)$^{-1}$ s^{-1}) is the average second-order reaction rate constant of $^{\bullet}$OH with DOM compiled from the literature,[54, 63-72] and [DOM] is the concentration of DOC.

The apparent quantum yield of $^{\bullet}$OH attributable to DOM, $\Phi_{\mathrm{app},\,^{\bullet}\mathrm{OH}}$ (mol mol-photons^{-1}), was calculated as:[48, 49]

$$\Phi_{\mathrm{app},\,^{\bullet}\mathrm{OH}} = \frac{R_{\mathrm{f},\,^{\bullet}\mathrm{OH}}}{R_{\mathrm{a}}} = \frac{R_{\mathrm{prod,\,hTPA}}^{\mathrm{corr}}}{R_{\mathrm{a}}Y_{\mathrm{hTPA}}} \quad \text{(S8)}$$

Note that $\Phi_{\mathrm{app},\,^{\bullet}\mathrm{OH}}$ were not corrected for $^{\bullet}$OH production from the photo-Fenton pathway[63, 68, 73] given the low [Fe] (i.e., 0.26±0.20 µM; Table S3) in leachates and whole water samples. $\Phi_{\mathrm{app},\,^{\bullet}\mathrm{OH}}$ are summarized in **Table S7**.

Table S7. $\Phi_{app, \cdot OH}$ for standardized leachates, whole water samples, and model DOM solutions

Sample Name	$\Phi_{app, \cdot OH}$ ($\times 10^{-5}$ mol mol-photons^{-1})	Sample Name	$\Phi_{app, \cdot OH}$ ($\times 10^{-5}$ mol mol-photons^{-1})
Soil Oa L Photo 0 h	1.75±0.05	Soil Oa L Bio 0 d	1.75±0.05
Soil Oa L Photo 1 h	1.41±0.01	Soil Oa L Bio 8 d	2.47±0.34
Soil Oa L Photo 2 h	1.32±0.03	Soil Oa L Bio 16 d	2.29±0.11
Soil Oa L Photo 4 h	1.19±0.05	Soil Oa L Bio 32 d	2.15±0.11
Soil Oa L Photo 8 h	1.07±0.05	Soil Oa L Photo-Bio 0 d	1.29±0.02
Soil Oa L Photo 16 h	1.01±0.07	Soil Oa L Photo-Bio 8 d	2.32±0.12
Soil Oa L Photo 32 h	0.92±0.09	Soil Oa L Photo-Bio 16 d	2.24±0.02
Soil Oa L Photo 64 h	0.87±0.17	Soil Oa L Photo-Bio 32 d	2.16±0.02
Soil Oa L Photo 96 h	0.80±0.08		
Sample Name	$\Phi_{app, \cdot OH}$ ($\times 10^{-5}$ mol mol-photons^{-1})	Sample Name	$\Phi_{app, \cdot OH}$ ($\times 10^{-5}$ mol mol-photons^{-1})
Soil Oa R Photo 0 h	1.67±0.01	Soil Oa R Bio 0 d	1.74±0.05
Soil Oa R Photo 1 h	1.36±0.03	Soil Oa R Bio 8 d	3.20±0.13
Soil Oa R Photo 2 h	1.25±0.07	Soil Oa R Bio 16 d	3.05±0.12
Soil Oa R Photo 4 h	1.13±0.06	Soil Oa R Bio 32 d	2.95±0.02
Soil Oa R Photo 8 h	1.03±0.01	Soil Oa R Photo-Bio 0 d	1.22±0.03
Soil Oa R Photo 16 h	0.98±0.01	Soil Oa R Photo-Bio 8 d	2.57±0.08
Soil Oa R Photo 32 h	0.87±0.02	Soil Oa R Photo-Bio 16 d	2.56±0.14
Soil Oa R Photo 64 h	0.78±0.02	Soil Oa R Photo-Bio 32 d	2.37±0.19
Soil Oa R Photo 96 h	0.69±0.01		
Sample Name	$\Phi_{app, \cdot OH}$ ($\times 10^{-5}$ mol mol-photons^{-1})	Sample Name	$\Phi_{app, \cdot OH}$ ($\times 10^{-5}$ mol mol-photons^{-1})
Leaf Photo 0 h	1.25±0.17	Leaf Bio 0 d	1.24±0.01
Leaf Photo 1 h	1.11±0.10	Leaf Bio 8 d	3.09±0.22
Leaf Photo 2 h	0.94±0.05	Leaf Bio 16 d	3.41±0.04
Leaf Photo 4 h	0.85±0.09	Leaf Bio 32 d	2.66±0.06
Leaf Photo 8 h	0.77±0.01	Leaf Photo-Bio 0 d	0.93±0.01
Leaf Photo 16 h	0.68±0.05	Leaf Photo-Bio 8 d	3.25±0.03
Leaf Photo 32 h	0.62±0.01	Leaf Photo-Bio 16 d	2.94±0.11
Leaf Photo 64 h	0.57±0.01	Leaf Photo-Bio 32 d	3.06±0.15
Leaf Photo 96 h	0.54±0.02		

"Photo" = photodegradation; "Bio" = biodegradation; "Photo-Bio" = photo-biodegradation; "L" = the limed tributary watershed W16L; "R" = the reference tributary watershed W24R. Errors represent one standard deviation from duplicate measurements.

Table S7. $\Phi_{app, \cdot OH}$ for standardized leachates, whole water samples, and model DOM solutions (continued)

Sample Name	$\Phi_{app, \cdot OH}$ (×10^{-5} mol mol-photons^{-1})	Sample Name	$\Phi_{app, \cdot OH}$ (×10^{-5} mol mol-photons^{-1})
Soil Oa L-L	1.75±0.12	SRFA	2.24±0.13
Soil Bs L-L	6.76±0.41	SRHA	1.29±0.09
Soil Oa L-M	1.78±0.20	SRNOM	2.11±0.11
Soil Bs L-M	6.83±0.27	PPFA	1.69±0.10
Soil Oa L-H	1.77±0.20	PPHA	0.85±0.08
Soil Bs L-H	6.86±0.26	NRNOM	2.28±0.07
Soil Oa R-L	1.69±0.06	UMRNOM	2.32±0.13
Soil Bs R-L	6.52±0.29	ESHA	1.41±0.11
Soil Oa R-M	1.69±0.20		
Soil Bs R-M	6.56±0.32		
Soil Oa R-H	1.69±0.19		
Soil Bs R-H	6.54±0.22		
Stream L	2.42±0.20		
Stream R	2.50±0.21		
Honnedaga Lake	2.30±0.22		
Sample Name	$\Phi_{app, \cdot OH}$ (×10^{-5} mol mol-photons^{-1})	Sample Name	$\Phi_{app, \cdot OH}$ (×10^{-5} mol mol-photons^{-1})
SRFA Photo 0 h	2.41±0.13	Glucose Bio 0 d	0.90±0.04
SRFA Photo 4 h	2.22±0.13	Glucose Bio 8 d	11.35±0.36
SRFA Photo 16 h	1.93±0.12	Glucose Bio 16 d	20.23±0.86
SRFA Photo 64 h	1.53±0.13	Glucose Bio 32 d	20.27±1.66
ESHA Photo 0 h	1.41±0.11	ESHA Bio 0 d	1.41±0.11
ESHA Photo 4 h	1.23±0.09	ESHA Bio 8 d	2.59±0.07
ESHA Photo 16 h	1.07±0.08	ESHA Bio 16 d	2.55±0.05
ESHA Photo 64 h	0.86±0.08	ESHA Bio 32 d	2.61±0.08

"Photo" = photodegradation; "Bio" = biodegradation; "Photo-Bio" = photo-biodegradation; "L" = the limed tributary watershed W16L; "R" = the reference tributary watershed W24R. Errors represent one standard deviation from duplicate measurements.

7. Furfuryl alcohol (FFA) as a probe for $^1O_2$

FFA was spiked into samples to measure the photoproduction of $^1O_2$. For each standardized sample, the loss of FFA was monitored to determine the pseudo-first order rate constant for the photodegradation of FFA, $k_{\text{obs, FFA}}$ (s^{-1}), with the contributions from *apparent* direct photolysis of FFA caused by impurities[38, 74] (4.5±5.0% for Honnedaga samples) and the reaction of FFA with $^{\bullet}OH$ (22.2±4.4% for Honnedaga samples):[75, 76]

$$R_{\text{loss, FFA}} = -\frac{\text{d[FFA]}}{\text{dt}} = k_{\text{obs, FFA}}[\text{FFA}]$$
$$= k_{\text{FFA, }^1\text{O}_2}[\text{FFA}][^1\text{O}_2]_{\text{ss}} + k_{\text{direct photolysis, FFA}}[\text{FFA}]SF_{\Sigma\lambda} + k_{\text{FFA, }^{\bullet}\text{OH}}[\text{FFA}][^{\bullet}\text{OH}]_{\text{ss}} \quad \text{(S9)}$$

where $R_{\text{loss, FFA}}$ ($M\ s^{-1}$) is the loss rate of FFA, [FFA] is the initial concentration of FFA (10 μM), $k_{\text{FFA, }^1O_2}$ ($M^{-1}\ s^{-1}$) is the second-order reaction rate constant of FFA with $^1O_2$, and $[^1O_2]_{ss}$ is the steady-state concentration of $^1O_2$, $k_{\text{direct photolysis, FFA}}$ (s^{-1}) is the experimentally determined *apparent* direct photolysis rate constant of FFA, $SF_{\Sigma\lambda}$ is the sample-specific light screening factor, $k_{\text{FFA, }^{\bullet}OH}$ ($1.5\times10^{10}\ M^{-1}\ s^{-1}$) is the second-order reaction rate constant of FFA with $^{\bullet}OH$,[62] and $[^{\bullet}OH]_{ss}$ is the steady-state concentration of $^{\bullet}OH$ measured by TPA.

To account for the potential effect of temperature on the $^1O_2$ reaction kinetics of FFA, a temperature-adjusted $k_{\text{FFA, }^1O_2}$ of $1.06(\pm0.07)\times10^8\ M^{-1}\ s^{-1}$ was derived by substituting T = 25 °C into $k_{\text{FFA, }^1O_2} = (1.00 \pm 0.04) \times 10^8\ M^{-1}\ s^{-1} + [(2.1 \pm 0.3) \times 10^6\ M^{-1}\ s^{-1}\ °C^{-1}] \times (T - 22\ °C)$.[39]

For each standardized sample, the steady-state concentration of $^1O_2$, $[^1O_2]_{ss}$, was calculated as:[38, 39, 48, 77]

$$[^1\text{O}_2]_{\text{ss}} = \frac{R_{\text{f, }^1\text{O}_2}}{k_{\text{d}}^{\Delta}} = \frac{(k_{\text{obs, FFA}} - k_{\text{direct photolysis, FFA}}SF_{\Sigma\lambda} - k_{\text{FFA, }^{\bullet}\text{OH}}[^{\bullet}\text{OH}]_{\text{ss}})(k_{\text{d}}^{\Delta} + k_{\text{FFA, }^1\text{O}_2}[\text{FFA}])}{k_{\text{d}}^{\Delta}k_{\text{FFA, }^1\text{O}_2}}$$
$$\approx \frac{(k_{\text{obs, FFA}} - k_{\text{direct photolysis, FFA}}SF_{\Sigma\lambda} - k_{\text{FFA, }^{\bullet}\text{OH}}[^{\bullet}\text{OH}]_{\text{ss}})}{k_{\text{FFA, }^1\text{O}_2}} \quad \text{(S10)}$$

where $R_{\text{f, }^1O_2}$ ($M\ s^{-1}$) is the formation rate of $^1O_2$ and k_d^{Δ} ($2.78(\pm0.03)\times10^5\ s^{-1}$; temperature-adjusted for T = 25 °C[39]) is the pseudo-first order deactivation rate constant of $^1O_2$ by water.

The formation rate of $^1O_2$, $R_{\text{f, }^1O_2}$ ($M\ s^{-1}$), was calculated as:

$$R_{\text{f, }^1\text{O}_2} = [^1\text{O}_2]_{\text{ss}}k_{\text{d}}^{\Delta} \quad \text{(S11)}$$

The apparent quantum yield of $^1O_2$, $\Phi_{app,\,^1O_2}$ (mol mol-photons^{-1}), was calculated as:[48, 77]

$$\Phi_{app,\,^1O_2} = \frac{R_{f,\,^1O_2}}{R_a} = \frac{(k_d^{\Delta} + k_{FFA,\,^1O_2}[FFA])[^1O_2]_{ss}}{R_a} \approx \frac{k_d^{\Delta}[^1O_2]_{ss}}{R_a} \qquad (S12)$$

To determine $\Phi_{app,\,^1O_2}$ attributable to high-energy $^3DOM^*$ (i.e., $\Phi_{app,\,^1O_2,\,high\text{-}energy}$) and low-energy $^3DOM^*$ (i.e., $\Phi_{app,\,^1O_2,\,low\text{-}energy}$), *t,t*-HDO (2 mM) was spiked into FFA-containing (10 μM) samples to preferentially quench high-energy $^3DOM^*$ capable of sensitizing *t,t*-HDO isomerization (i.e., $^3DOM^*$ with $E_T \geq 250$ kJ mol^{-1} [78]). The percentage contribution of $\Phi_{app,\,^1O_2,\,high\text{-}energy}$ to $\Phi_{app,\,^1O_2}$ and the percentage contribution of $\Phi_{app,\,^1O_2,\,low\text{-}energy}$ to $\Phi_{app,\,^1O_2}$ were calculated as:[79]

$$\%\Phi_{app,\,^1O_2,\,high\text{-}energy} = \%\Phi_{app,\,^1O_2\_^3DOM^*_{HDO}} = \frac{(\Phi_{app,\,^1O_2} - \Phi_{app,\,^1O_2,\,HDO})}{\Phi_{app,\,^1O_2}} \times 100\% \qquad (S13)$$

$$\%\Phi_{app,\,^1O_2,\,low\text{-}energy} = \frac{\Phi_{app,\,^1O_2,\,HDO}}{\Phi_{app,\,^1O_2}} \times 100\% \qquad (S14)$$

$\Phi_{app,\,^1O_2}$, $\Phi_{app,\,^1O_2,\,high\text{-}energy}$, and $\Phi_{app,\,^1O_2,\,low\text{-}energy}$ are summarized in **Table S8**. $\%\Phi_{app,\,^1O_2,\,high\text{-}energy}$ and $\%\Phi_{app,\,^1O_2,\,low\text{-}energy}$ are summarized in **Table S9**.

**Table S8.** $\Phi_{app, {}^1O_2}$ for standardized leachates, whole water samples, and model DOM solutions			
Sample Name	$\Phi_{app, {}^1O_2}$ ($\times10^{-2}$ mol mol-photons^{-1})	$\Phi_{app, {}^1O_2, high-energy}$ ($\times10^{-2}$ mol mol-photons^{-1})	$\Phi_{app, {}^1O_2, low-energy}$ ($\times10^{-2}$ mol mol-photons^{-1})
Soil Oa L Photo 0 h	1.56±0.10	0.42±0.04	1.14±0.14
Soil Oa L Photo 1 h	1.11±0.06	0.42±0.04	0.69±0.11
Soil Oa L Photo 2 h	1.03±0.09	0.41±0.02	0.62±0.10
Soil Oa L Photo 4 h	0.92±0.07	0.41±0.03	0.51±0.11
Soil Oa L Photo 8 h	0.81±0.01	0.41±0.08	0.40±0.07
Soil Oa L Photo 16 h	0.75±0.02	0.40±0.07	0.35±0.05
Soil Oa L Photo 32 h	0.70±0.04	0.40±0.09	0.30±0.04
Soil Oa L Photo 64 h	0.65±0.12	0.40±0.04	0.25±0.09
Soil Oa L Photo 96 h	0.58±0.03	0.40±0.08	0.19±0.05
Sample Name	$\Phi_{app, {}^1O_2}$ ($\times10^{-2}$ mol mol-photons^{-1})	$\Phi_{app, {}^1O_2, high-energy}$ ($\times10^{-2}$ mol mol-photons^{-1})	$\Phi_{app, {}^1O_2, low-energy}$ ($\times10^{-2}$ mol mol-photons^{-1})
Soil Oa L Bio 0 d	1.56±0.11	0.42±0.06	1.15±0.17
Soil Oa L Bio 8 d	2.49±0.15	0.43±0.11	2.06±0.26
Soil Oa L Bio 16 d	3.19±0.20	0.44±0.07	2.75±0.27
Soil Oa L Bio 32 d	3.36±0.26	0.44±0.04	2.92±0.30
Soil Oa L Photo-Bio 0 d	1.03±0.02	0.41±0.06	0.62±0.08
Soil Oa L Photo-Bio 8 d	1.35±0.07	0.42±0.02	0.93±0.09
Soil Oa L Photo-Bio 16 d	1.61±0.10	0.43±0.02	1.18±0.13
Soil Oa L Photo-Bio 32 d	1.87±0.15	0.43±0.03	1.43±0.12
Sample Name	$\Phi_{app, {}^1O_2}$ ($\times10^{-2}$ mol mol-photons^{-1})	$\Phi_{app, {}^1O_2, high-energy}$ ($\times10^{-2}$ mol mol-photons^{-1})	$\Phi_{app, {}^1O_2, low-energy}$ ($\times10^{-2}$ mol mol-photons^{-1})
Soil Oa R Photo 0 h	1.56±0.08	0.41±0.02	1.14±0.10
Soil Oa R Photo 1 h	1.23±0.01	0.43±0.06	0.80±0.07
Soil Oa R Photo 2 h	1.14±0.10	0.43±0.12	0.71±0.03
Soil Oa R Photo 4 h	1.01±0.07	0.42±0.05	0.59±0.12
Soil Oa R Photo 8 h	0.92±0.05	0.42±0.11	0.49±0.06
Soil Oa R Photo 16 h	0.81±0.04	0.42±0.05	0.39±0.09
Soil Oa R Photo 32 h	0.76±0.02	0.42±0.07	0.34±0.10
Soil Oa R Photo 64 h	0.70±0.02	0.41±0.06	0.29±0.07
Soil Oa R Photo 96 h	0.65±0.05	0.41±0.03	0.24±0.08
Sample Name	$\Phi_{app, {}^1O_2}$ ($\times10^{-2}$ mol mol-photons^{-1})	$\Phi_{app, {}^1O_2, high-energy}$ ($\times10^{-2}$ mol mol-photons^{-1})	$\Phi_{app, {}^1O_2, low-energy}$ ($\times10^{-2}$ mol mol-photons^{-1})
Soil Oa R Bio 0 d	1.60±0.14	0.43±0.01	1.17±0.14
Soil Oa R Bio 8 d	2.78±0.19	0.44±0.13	2.34±0.33
Soil Oa R Bio 16 d	3.39±0.31	0.45±0.01	2.93±0.29
Soil Oa R Bio 32 d	3.57±0.27	0.46±0.02	3.12±0.25
Soil Oa R Photo-Bio 0 d	1.14±0.08	0.42±0.02	0.72±0.10
Soil Oa R Photo-Bio 8 d	1.51±0.14	0.44±0.03	1.07±0.11
Soil Oa R Photo-Bio 16 d	1.68±0.15	0.44±0.02	1.24±0.13
Soil Oa R Photo-Bio 32 d	2.03±0.15	0.45±0.03	1.58±0.18

"Photo" = photodegradation; "Bio" = biodegradation; "Photo-Bio" = photo-biodegradation; "L" = the limed tributary watershed W16L; "R" = the reference tributary watershed W24R. Errors represent one standard deviation from duplicate measurements.

**Table S8.** $\Phi_{app, {}^1O_2}$ for standardized leachates, whole water samples, and model DOM solutions (continued)			
Sample Name	$\Phi_{app, {}^1O_2}$ ($\times 10^{-2}$ mol mol-photons^{-1})	$\Phi_{app, {}^1O_2, high-energy}$ ($\times 10^{-2}$ mol mol-photons^{-1})	$\Phi_{app, {}^1O_2, low-energy}$ ($\times 10^{-2}$ mol mol-photons^{-1})
Leaf Photo 0 h	1.42±0.12	0.39±0.06	1.04±0.07
Leaf Photo 1 h	1.08±0.02	0.38±0.04	0.69±0.02
Leaf Photo 2 h	0.93±0.01	0.37±0.05	0.56±0.04
Leaf Photo 4 h	0.87±0.01	0.37±0.01	0.50±0.01
Leaf Photo 8 h	0.77±0.04	0.37±0.02	0.40±0.06
Leaf Photo 16 h	0.72±0.02	0.36±0.02	0.36±0.01
Leaf Photo 32 h	0.66±0.04	0.36±0.02	0.30±0.02
Leaf Photo 64 h	0.61±0.01	0.36±0.01	0.25±0.01
Leaf Photo 96 h	0.55±0.04	0.36±0.02	0.20±0.02
Sample Name	$\Phi_{app, {}^1O_2}$ ($\times 10^{-2}$ mol mol-photons^{-1})	$\Phi_{app, {}^1O_2, high-energy}$ ($\times 10^{-2}$ mol mol-photons^{-1})	$\Phi_{app, {}^1O_2, low-energy}$ ($\times 10^{-2}$ mol mol-photons^{-1})
Leaf Bio 0 d	1.42±0.10	0.38±0.01	1.04±0.09
Leaf Bio 8 d	2.38±0.01	0.40±0.30	1.98±0.29
Leaf Bio 16 d	2.81±0.10	0.41±0.01	2.40±0.10
Leaf Bio 32 d	3.10±0.01	0.41±0.15	2.69±0.15
Leaf Photo-Bio 0 d	0.94±0.04	0.38±0.04	0.56±0.08
Leaf Photo-Bio 8 d	1.36±0.05	0.39±0.04	0.97±0.09
Leaf Photo-Bio 16 d	1.79±0.09	0.40±0.03	1.39±0.13
Leaf Photo-Bio 32 d	1.98±0.15	0.40±0.01	1.58±0.16
Sample Name	$\Phi_{app, {}^1O_2}$ ($\times 10^{-2}$ mol mol-photons^{-1})	$\Phi_{app, {}^1O_2, high-energy}$ ($\times 10^{-2}$ mol mol-photons^{-1})	$\Phi_{app, {}^1O_2, low-energy}$ ($\times 10^{-2}$ mol mol-photons^{-1})
Soil Oa L-L	1.56±0.12	0.41±0.07	1.15±0.19
Soil Bs L-L	5.97±0.44	1.36±0.17	4.61±0.61
Soil Oa L-M	1.57±0.09	0.42±0.07	1.15±0.16
Soil Bs L-M	5.78±0.87	1.31±0.20	4.48±1.07
Soil Oa L-H	1.56±0.10	0.41±0.12	1.15±0.21
Soil Bs L-H	6.12±0.69	1.39±0.29	4.73±0.98
Soil Oa R-L	1.59±0.13	0.43±0.10	1.16±0.23
Soil Bs R-L	6.03±0.52	1.39±0.33	4.64±0.85
Soil Oa R-M	1.60±0.10	0.43±0.08	1.16±0.18
Soil Bs R-M	5.94±0.60	1.38±0.27	4.57±0.87
Soil Oa R-H	1.60±0.09	0.41±0.08	1.19±0.17
Soil Bs R-H	5.84±0.75	1.35±0.27	4.49±0.48
Stream L	2.33±0.17	0.63±0.09	1.69±0.25
Stream R	2.39±0.16	0.64±0.09	1.75±0.26
Honnedaga Lake	1.97±0.15	0.51±0.07	1.45±0.22

"Photo" = photodegradation; "Bio" = biodegradation; "Photo-Bio" = photo-biodegradation; "L" = the limed tributary watershed W16L; "R" = the reference tributary watershed W24R. Errors represent one standard deviation from duplicate measurements.

**Table S8.** $\Phi_{app, {}^1O_2}$ for standardized leachates, whole water samples, and model DOM solutions (continued)			
Sample Name	$\Phi_{app, {}^1O_2}$ ($\times 10^{-2}$ mol mol-photons^{-1})	$\Phi_{app, {}^1O_2, high\text{-}energy}$ ($\times 10^{-2}$ mol mol-photons^{-1})	$\Phi_{app, {}^1O_2, low\text{-}energy}$ ($\times 10^{-2}$ mol mol-photons^{-1})
SRFA	2.58±0.18	1.14±0.08	1.42±0.20
SRHA	1.47±0.01	0.44±0.07	1.02±0.07
SRNOM	2.39±0.15	1.04±0.07	1.35±0.08
PPFA	2.57±0.08	1.15±0.17	1.42±0.25
PPHA	1.57±0.09	0.32±0.04	1.25±0.13
NRNOM	2.78±0.10	1.27±0.19	1.50±0.29
UMRNOM	2.75±0.22	1.31±0.11	1.44±0.33
ESHA	1.41±0.07	0.32±0.04	1.09±0.07
Sample Name	$\Phi_{app, {}^1O_2}$ ($\times 10^{-2}$ mol mol-photons^{-1})	$\Phi_{app, {}^1O_2, high\text{-}energy}$ ($\times 10^{-2}$ mol mol-photons^{-1})	$\Phi_{app, {}^1O_2, low\text{-}energy}$ ($\times 10^{-2}$ mol mol-photons^{-1})
SRFA Photo 0 h	2.66±0.23	1.19±0.02	1.47±0.21
SRFA Photo 4 h	2.63±0.23	1.18±0.18	1.44±0.05
SRFA Photo 16 h	2.56±0.16	1.17±0.09	1.39±0.25
SRFA Photo 64 h	2.42±0.21	1.13±0.03	1.29±0.24
ESHA Photo 0 h	1.41±0.09	0.32±0.02	1.09±0.07
ESHA Photo 4 h	1.30±0.05	0.31±0.09	0.99±0.14
ESHA Photo 16 h	1.20±0.10	0.29±0.01	0.91±0.10
ESHA Photo 64 h	1.08±0.10	0.27±0.05	0.81±0.05
Glucose Bio 0 d	0.25±0.03	0.13±0.05	0.12±0.02
Glucose Bio 8 d	4.22±0.31	1.22±0.20	3.00±0.30
Glucose Bio 16 d	5.26±0.22	1.21±0.13	4.05±0.30
Glucose Bio 32 d	6.36±0.39	1.30±0.15	5.06±0.31
ESHA Bio 0 d	1.42±0.08	0.32±0.06	1.10±0.14
ESHA Bio 8 d	1.43±0.06	0.33±0.01	1.11±0.06
ESHA Bio 16 d	1.43±0.08	0.33±0.02	1.11±0.11
ESHA Bio 32 d	1.44±0.07	0.32±0.08	1.12±0.14
"Photo" = photodegradation; "Bio" = biodegradation; "Photo-Bio" = photo-biodegradation; "L" = the limed tributary watershed W16L; "R" = the reference tributary watershed W24R. Errors represent one standard deviation from duplicate measurements.			

**Table S9.** $\%\Phi_{app,\ ^1O_2,\ high\text{-}energy}$ and $\%\Phi_{app,\ ^1O_2,\ low\text{-}energy}$ for standardized leachates, whole water samples, and model DOM solutions		
Sample Name	$\%\Phi_{app,\ ^1O_2,\ high\text{-}energy}$	$\%\Phi_{app,\ ^1O_2,\ low\text{-}energy}$
Soil Oa L Photo 0 h	26.9±4.3	73.2±10.3
Soil Oa L Photo 1 h	37.9±6.1	62.3±10.4
Soil Oa L Photo 2 h	40.4±5.0	59.8±11.1
Soil Oa L Photo 4 h	45.1±7.3	55.2±12.5
Soil Oa L Photo 8 h	50.2±9.5	49.8±9.2
Soil Oa L Photo 16 h	53.5±8.1	46.4±7.2
Soil Oa L Photo 32 h	57.2±8.9	42.5±6.8
Soil Oa L Photo 64 h	62.1±6.4	38.6±15.6
Soil Oa L Photo 96 h	67.8±10.4	31.9±9.0
Sample Name	$\%\Phi_{app,\ ^1O_2,\ high\text{-}energy}$	$\%\Phi_{app,\ ^1O_2,\ low\text{-}energy}$
Soil Oa L Bio 0 d	26.8±5.6	73.4±11.9
Soil Oa L Bio 8 d	17.5±5.4	82.7±11.6
Soil Oa L Bio 16 d	13.8±3.0	86.3±10.2
Soil Oa L Bio 32 d	13.3±2.2	86.8±11.2
Soil Oa L Photo-Bio 0 d	40.0±7.0	60.1±8.2
Soil Oa L Photo-Bio 8 d	31.5±2.9	68.6±7.5
Soil Oa L Photo-Bio 16 d	26.8±3.0	73.3±9.1
Soil Oa L Photo-Bio 32 d	23.3±0.3	76.7±8.8
Sample Name	$\%\Phi_{app,\ ^1O_2,\ high\text{-}energy}$	$\%\Phi_{app,\ ^1O_2,\ low\text{-}energy}$
Soil Oa R Photo 0 h	26.6±2.8	73.4±7.5
Soil Oa R Photo 1 h	34.7±4.9	65.3±5.4
Soil Oa R Photo 2 h	37.0±7.8	62.6±5.9
Soil Oa R Photo 4 h	42.2±7.7	58.0±12.4
Soil Oa R Photo 8 h	45.8±9.3	54.0±7.0
Soil Oa R Photo 16 h	51.8±8.7	48.4±11.1
Soil Oa R Photo 32 h	54.9±11.1	45.3±12.6
Soil Oa R Photo 64 h	58.7±9.4	41.4±10.4
Soil Oa R Photo 96 h	64.1±9.7	36.3±13.0
Sample Name	$\%\Phi_{app,\ ^1O_2,\ high\text{-}energy}$	$\%\Phi_{app,\ ^1O_2,\ low\text{-}energy}$
Soil Oa R Bio 0 d	27.1±2.3	73.0±11.0
Soil Oa R Bio 8 d	16.0±6.0	84.2±13.2
Soil Oa R Bio 16 d	13.4±0.8	86.6±11.7
Soil Oa R Bio 32 d	12.9±0.3	87.2±9.6
Soil Oa R Photo-Bio 0 d	37.3±4.6	62.8±10.0
Soil Oa R Photo-Bio 8 d	29.0±0.8	71.0±10.1
Soil Oa R Photo-Bio 16 d	26.4±1.2	73.7±10.2
Soil Oa R Photo-Bio 32 d	22.2±3.2	77.9±10.4

"Photo" = photodegradation; "Bio" = biodegradation; "Photo-Bio" = photo-biodegradation; "L" = the limed tributary watershed W16L; "R" = the reference tributary watershed W24R. Errors represent one standard deviation from duplicate measurements.

**Table S9.** %$\Phi_{app,\ ^1O_2,\ high\text{-}energy}$ and %$\Phi_{app,\ ^1O_2,\ low\text{-}energy}$ for standardized leachates, whole water samples, and model DOM solutions (continued)		
Sample Name	%$\Phi_{app,\ ^1O_2,\ high\text{-}energy}$	%$\Phi_{app,\ ^1O_2,\ low\text{-}energy}$
Leaf Photo 0 h	27.1±1.6	72.9±7.7
Leaf Photo 1 h	35.6±3.3	64.4±2.5
Leaf Photo 2 h	39.9±4.7	60.1±4.3
Leaf Photo 4 h	42.6±0.8	57.4±1.0
Leaf Photo 8 h	47.9±5.2	52.2±8.4
Leaf Photo 16 h	50.1±1.3	49.9±1.1
Leaf Photo 32 h	54.4±0.1	45.6±3.5
Leaf Photo 64 h	59.0±0.6	41.0±0.8
Leaf Photo 96 h	64.7±0.5	35.3±3.9
Sample Name	%$\Phi_{app,\ ^1O_2,\ high\text{-}energy}$	%$\Phi_{app,\ ^1O_2,\ low\text{-}energy}$
Leaf Bio 0 d	27.1±1.3	72.9±8.5
Leaf Bio 8 d	16.8±12.6	83.2±12.3
Leaf Bio 16 d	14.5±0.5	85.5±4.5
Leaf Bio 32 d	13.3±4.7	86.7±4.9
Leaf Photo-Bio 0 d	40.3±6.4	59.8±9.4
Leaf Photo-Bio 8 d	28.9±3.8	71.2±7.0
Leaf Photo-Bio 16 d	22.3±3.0	77.7±8.1
Leaf Photo-Bio 32 d	20.4±2.0	79.7±10.1
Sample Name	%$\Phi_{app,\ ^1O_2,\ high\text{-}energy}$	%$\Phi_{app,\ ^1O_2,\ low\text{-}energy}$
Soil Oa L-L	26.5±6.5	73.8±13.2
Soil Bs L-L	22.9±4.6	77.2±11.8
Soil Oa L-M	26.9±6.3	73.3±11.3
Soil Bs L-M	23.1±6.9	77.4±21.8
Soil Oa L-H	26.3±9.1	73.9±14.3
Soil Bs L-H	23.1±7.4	77.3±18.3
Soil Oa R-L	27.2±8.5	73.2±15.6
Soil Bs R-L	23.4±7.5	76.9±15.6
Soil Oa R-M	27.4±6.5	72.9±12.2
Soil Bs R-M	23.5±6.8	76.8±16.5
Soil Oa R-H	25.8±6.4	74.4±11.7
Soil Bs R-H	23.0±1.7	76.9±12.9
Stream L	27.4±5.7	72.8±12.1
Stream R	27.0±5.7	73.2±11.7
Honnedaga Lake	26.4±5.5	73.8±12.3

"Photo" = photodegradation; "Bio" = biodegradation; "Photo-Bio" = photo-biodegradation; "L" = the limed tributary watershed W16L; "R" = the reference tributary watershed W24R. Errors represent one standard deviation from duplicate measurements.

Table S9. $\%\Phi_{app,\ ^1O_2,\ high\text{-}energy}$ and $\%\Phi_{app,\ ^1O_2,\ low\text{-}energy}$ for standardized leachates, whole water samples, and model DOM solutions (continued)

Sample Name	$\%\Phi_{app,\ ^1O_2,\ high\text{-}energy}$	$\%\Phi_{app,\ ^1O_2,\ low\text{-}energy}$
SRFA	45.5±6.9	54.7±10.4
SRHA	30.3±4.7	69.7±5.0
SRNOM	43.6±0.1	56.4±4.9
PPFA	44.9±7.9	55.2±9.8
PPHA	20.4±3.5	79.7±9.3
NRNOM	46.0±8.6	54.1±10.6
UMRNOM	47.9±8.0	52.4±12.8
ESHA	22.8±3.2	77.3±10.1
Sample Name	$\%\Phi_{app,\ ^1O_2,\ high\text{-}energy}$	$\%\Phi_{app,\ ^1O_2,\ low\text{-}energy}$
SRFA Photo 0 h	44.8±3.1	55.3±9.1
SRFA Photo 4 h	44.9±3.0	55.0±5.2
SRFA Photo 16 h	45.9±6.3	54.3±10.3
SRFA Photo 64 h	47.0±5.5	53.2±11.0
ESHA Photo 0 h	23.0±0.1	77.0±7.3
ESHA Photo 4 h	23.9±7.6	76.2±10.9
ESHA Photo 16 h	24.4±1.9	75.7±10.4
ESHA Photo 64 h	24.7±2.3	75.2±8.8
Glucose Bio 0 d	49.5±13.3	49.5±7.8
Glucose Bio 8 d	29.0±4.6	71.1±6.8
Glucose Bio 16 d	23.1±3.1	77.0±6.7
Glucose Bio 32 d	20.5±1.8	79.5±5.5
ESHA Bio 0 d	22.6±5.4	77.5±10.8
ESHA Bio 8 d	22.8±0.6	77.2±5.1
ESHA Bio 16 d	22.8±2.9	77.2±8.7
ESHA Bio 32 d	22.3±6.5	77.8±10.7

"Photo" = photodegradation; "Bio" = biodegradation; "Photo-Bio" = photo-biodegradation; "L" = the limed tributary watershed W16L; "R" = the reference tributary watershed W24R. Errors represent one standard deviation from duplicate measurements.

8. 2,4,6-Trimethylphenol (TMP) as an electron transfer probe for $^3DOM^*$

TMP was spiked into samples to measure the photoproduction of $^3DOM^*_{TMP}$. For each standardized sample, the loss of TMP was monitored to determine the pseudo-first order rate constant for the photodegradation of TMP, $k_{obs, TMP}$ (s^{-1}), with the negligible contribution from direct photolysis of TMP (1.3±2.0% for Honnedaga samples)[80] and its reactions with $^{\bullet}OH$ (4.5±1.6% for Honnedaga samples) and $^1O_2$ (6.5±1.5% for Honnedaga samples):[40, 81-89]

$$R_{loss, TMP} = -\frac{d[TMP]}{dt} = k_{obs, TMP}[TMP]$$

$$= k_{TMP, ^3DOM^*_{TMP}}[TMP][^3DOM^*_{TMP}]_{ss} + k_{direct\ photolysis, TMP}[TMP]SF_{\Sigma\lambda} + k_{TMP, ^1O_2}[TMP][^1O_2]_{ss} + k_{TMP, ^{\bullet}OH}[TMP][^{\bullet}OH]_{ss} \quad (S15)$$

where $R_{loss, TMP}$ (M s^{-1}) is the loss rate of TMP, [TMP] is the initial concentration of TMP (10 μM), $k_{TMP, ^3DOM^*_{TMP}}$ (M^{-1} s^{-1}) is the second-order reaction rate constant of TMP with $^3DOM^*_{TMP}$, $[^3DOM^*_{TMP}]_{ss}$ is the steady-state concentration of $^3DOM^*_{TMP}$, $k_{direct\ photolysis, TMP}$ (s^{-1}) is the experimentally determined direct photolysis rate constant of TMP, $SF_{\Sigma\lambda}$ is the sample-specific light screening factor, $k_{TMP, ^1O_2}$ ($5.1(\pm0.2)\times10^7$ M^{-1} s^{-1}) is the second-order reaction rate constant of TMP with $^1O_2$,[90] $[^1O_2]_{ss}$ is the steady-state concentration of $^1O_2$ measured by FFA, $k_{TMP, ^{\bullet}OH}$ ($1.6(\pm0.1)\times10^{10}$ M^{-1} s^{-1}) is the estimated second-order reaction rate constant of TMP with $^{\bullet}OH$,[62, 91] and $[^{\bullet}OH]_{ss}$ is the steady-state concentration of $^{\bullet}OH$ measured by TPA.

To account for the inhibition of TMP loss by reduced DOM moieties,[92-96] the pseudo-first order rate constant for the loss of TMP attributable to $^3DOM^*$ was further corrected for DOM-induced inhibition:[41, 82]

$$k^{corr}_{obs, TMP} = \frac{(k_{obs, TMP} - k_{direct\ photolysis, TMP}SF_{\Sigma\lambda} - k_{TMP, ^1O_2}[^1O_2]_{ss} - k_{TMP, ^{\bullet}OH}[^{\bullet}OH]_{ss})}{IF_{TMP}} \quad (S16)$$

where $k^{corr}_{obs, TMP}$ (s^{-1}) is the pseudo-first order rate constant for the loss of TMP attributable to $^3DOM^*$ corrected for inhibition and IF_{TMP} is the inhibition factor predicted from $1/IF_{TMP} = 0.021[DOC] + 0.965$ [41] using [DOC] (i.e., 4 mg/L) for standardized leachate and water samples.

For each standardized sample, the steady-state concentration of $^3DOM^*_{TMP}$, $[^3DOM^*_{TMP}]_{ss}$, was calculated as:[82, 97]

$$[^3\mathrm{DOM}^*_{\mathrm{TMP}}]_{\mathrm{ss}} = \frac{R_{\mathrm{f},\,^3\mathrm{DOM}^*_{\mathrm{TMP}}}}{k'_{\mathrm{q},\,^3\mathrm{DOM}^*_{\mathrm{TMP}}}} = \frac{k^{\mathrm{corr}}_{\mathrm{obs,\,TMP}}(k'_{\mathrm{q},\,^3\mathrm{DOM}^*_{\mathrm{TMP}}} + k_{\mathrm{TMP},\,^3\mathrm{DOM}^*_{\mathrm{TMP}}}[\mathrm{TMP}])}{k'_{\mathrm{q},\,^3\mathrm{DOM}^*_{\mathrm{TMP}}}k_{\mathrm{TMP},\,^3\mathrm{DOM}^*_{\mathrm{TMP}}}} \approx \frac{k^{\mathrm{corr}}_{\mathrm{obs,\,TMP}}}{k_{\mathrm{TMP},\,^3\mathrm{DOM}^*_{\mathrm{TMP}}}} \quad \text{(S17)}$$

where $R_{\mathrm{f},\,^3\mathrm{DOM}^*_{\mathrm{TMP}}}$ (M s^{-1}) is the formation rate of $^3DOM^*_{TMP}$, $k'_{\mathrm{q},\,^3\mathrm{DOM}^*_{\mathrm{TMP}}}$ ($3.2(\pm0.6)\times10^5$ s^{-1}; note that $k'_{\mathrm{q},\,^3\mathrm{DOM}^*_{\mathrm{TMP}}} \gg k_{\mathrm{TMP},\,^3\mathrm{DOM}^*_{\mathrm{TMP}}}[\mathrm{TMP}]$ with a maximum value of $9.7(\pm1.2)\times10^3$ s^{-1} at [TMP] = 10 μM) is the sum of pseudo-first order rate constants for $^3DOM^*_{TMP}$ quenching via energy transfer to dissolved O_2 ($2.3(\pm0.2)\times10^5$ s^{-1} calculated from $k_{O_2}[O_{2(aq)}]$ where $k_{O_2} = 8.9(\pm0.6)\times10^8$ M^{-1} s^{-1} and $[O_{2(aq)}]$ = ~258 μM at T = 25 °C[98]) and via other non-O_2 dependent nonradiative relaxation pathways ($k^{\mathrm{T}}_{\mathrm{d}} = 9.0(\pm2.8)\times10^4$ s^{-1}).[95, 96]

To solve for $k_{\mathrm{TMP},\,^3\mathrm{DOM}^*_{\mathrm{TMP}}}$ (**Table S10**), a linear regression of [TMP] (at varying concentrations of 10, 20, 50, 100, and 250 μM) and $k^{\mathrm{corr}}_{\mathrm{obs,\,TMP}}$ data was performed using the linearized form of Equation S18 (i.e., $y = ax + b$ where $y = 1/k^{\mathrm{corr,IF}}_{\mathrm{obs,\,TMP}}$ and x = [TMP]):[41, 47, 82, 99, 100]

$$\frac{1}{k^{\mathrm{corr}}_{\mathrm{obs,\,TMP}}} = \frac{[\mathrm{TMP}]}{R_{\mathrm{f},\,^3\mathrm{DOM}^*_{\mathrm{TMP}}}} + \frac{k'_{\mathrm{q},\,^3\mathrm{DOM}^*_{\mathrm{TMP}}}}{R_{\mathrm{f},\,^3\mathrm{DOM}^*_{\mathrm{TMP}}}k_{\mathrm{TMP},\,^3\mathrm{DOM}^*_{\mathrm{TMP}}}} \quad \text{(S18)}$$

The formation rate of $^3DOM^*_{TMP}$ (at [TMP] = 10 μM), $R_{\mathrm{f},\,^3\mathrm{DOM}^*_{\mathrm{TMP}}}$ (M s^{-1}), was calculated as:

$$R_{\mathrm{f},\,^3\mathrm{DOM}^*_{\mathrm{TMP}}} = [^3\mathrm{DOM}^*_{\mathrm{TMP}}]_{\mathrm{ss}}k'_{\mathrm{q},\,^3\mathrm{DOM}^*_{\mathrm{TMP}}} \quad \text{(S19)}$$

The apparent quantum yield of $^3DOM^*_{TMP}$, $\Phi_{\mathrm{app},\,^3\mathrm{DOM}^*_{\mathrm{TMP}}}$ (mol mol-photons^{-1}), was calculated as:[41, 47, 82, 101]

$$\Phi_{\mathrm{app},\,^3\mathrm{DOM}^*_{\mathrm{TMP}}} = \frac{R_{\mathrm{f},\,^3\mathrm{DOM}^*_{\mathrm{TMP}}}}{R_{\mathrm{a}}} \approx \frac{[^3\mathrm{DOM}^*_{\mathrm{TMP}}]_{\mathrm{ss}}k'_{\mathrm{q},\,^3\mathrm{DOM}^*_{\mathrm{TMP}}}}{R_{\mathrm{a}}} \quad \text{(S20)}$$

To determine $\Phi_{\mathrm{app},\,^3\mathrm{DOM}^*_{\mathrm{TMP}}}$ attributable to high-energy $^3DOM^*$ (i.e., $\Phi_{\mathrm{app},\,^3\mathrm{DOM}^*_{\mathrm{TMP},\,\mathrm{high\text{-}energy}}}$) and low-energy $^3DOM^*$ (i.e., $\Phi_{\mathrm{app},\,^3\mathrm{DOM}^*_{\mathrm{TMP},\,\mathrm{low\text{-}energy}}}$), *t,t*-HDO (2 mM) was spiked into TMP-containing (10 μM) samples to preferentially quench high-energy $^3DOM^*$ capable of sensitizing *t,t*-HDO isomerization (i.e., $^3DOM^*$ with $E_T \geq$

250 kJ mol^{-1} [78]). The percentage contribution of $\Phi_{app,\ ^3DOM^*_{TMP},\ high\text{-}energy}$ to $\Phi_{app,\ ^3DOM^*_{TMP}}$ and the percentage contribution of $\Phi_{app,\ ^3DOM^*_{TMP},\ low\text{-}energy}$ to $\Phi_{app,\ ^3DOM^*_{TMP}}$ were calculated as:[79]

$$\%\Phi_{app,\ ^3DOM^*_{TMP},\ high\text{-}energy} = \%\Phi_{app,\ ^3DOM^*_{TMP}\_^3DOM^*_{HDO}} = \frac{(\Phi_{app,\ ^3DOM^*_{TMP}} - \Phi_{app,\ ^3DOM^*_{TMP},\ HDO})}{\Phi_{app,\ ^3DOM^*_{TMP}}} \times 100\% \quad (S21)$$

$$\%\Phi_{app,\ ^3DOM^*_{TMP},\ low\text{-}energy} = \frac{\Phi_{app,\ ^3DOM^*_{TMP},\ HDO}}{\Phi_{app,\ ^3DOM^*_{TMP}}} \times 100\% \quad (S22)$$

$\Phi_{app,\ ^3DOM^*_{TMP}}$, $\Phi_{app,\ ^3DOM^*_{TMP},\ high\text{-}energy}$, and $\Phi_{app,\ ^3DOM^*_{TMP},\ low\text{-}energy}$ are summarized in **Table S11**. $\%\Phi_{app,\ ^3DOM^*_{TMP},\ high\text{-}energy}$ and $\%\Phi_{app,\ ^3DOM^*_{TMP},\ low\text{-}energy}$ are summarized in **Table S12**.

Table S10. $k_{\mathrm{TMP},\,^3\mathrm{DOM}^*_{\mathrm{TMP}}}$ for selected standardized leachates and whole water samples

Sample Name	$k_{\mathrm{TMP},\,^3\mathrm{DOM}^*_{\mathrm{TMP}}}$ ($\times 10^8$ M^{-1} s^{-1})
Soil Oa L Photo 0 h	8.63±0.80
Soil Oa L Photo 64 h	7.85±0.26
Soil Oa L Photo 96 h	7.64±0.77
Soil Oa R Photo 0 h	8.81±0.79
Soil Oa R Photo 64 h	8.00±0.69
Leaf Photo 0 h	8.89±1.00
Leaf Photo 16 h	8.50±0.22
Leaf Photo 96 h	8.22±0.64
Soil Oa L Bio 32 d	9.28±0.58
Soil Oa R Bio 32 d	9.29±1.15
Leaf Bio 32 d	9.69±1.16
Stream L	8.71±0.46
Stream R	8.43±0.39
Honnedaga Lake	8.99±0.71

"Photo" = photodegradation; "Bio" = biodegradation; "L" = the limed tributary watershed W16L; "R" = the reference tributary watershed W24R. Errors represent one standard deviation from duplicate measurements. Only selected samples were analyzed due to limited sample volumes.

**Table S11.** $\Phi_{app,\ ^3DOM^*_{TMP}}$ for standardized leachates, whole water samples, and model DOM solutions			
Sample Name	$\Phi_{app,\ ^3DOM^*_{TMP}}$ ($\times10^{-2}$ mol mol-photons^{-1})	$\Phi_{app,\ ^3DOM^*_{TMP},\ high\text{-}energy}$ ($\times10^{-2}$ mol mol-photons^{-1})	$\Phi_{app,\ ^3DOM^*_{TMP},\ low\text{-}energy}$ ($\times10^{-2}$ mol mol-photons^{-1})
Soil Oa L Photo 0 h	1.73±0.13	0.97±0.08	0.76±0.04
Soil Oa L Photo 1 h	1.05±0.05	0.56±0.01	0.49±0.04
Soil Oa L Photo 2 h	0.94±0.06	0.49±0.02	0.45±0.04
Soil Oa L Photo 4 h	0.77±0.03	0.37±0.02	0.40±0.05
Soil Oa L Photo 8 h	0.62±0.02	0.27±0.03	0.35±0.01
Soil Oa L Photo 16 h	0.53±0.04	0.19±0.02	0.34±0.02
Soil Oa L Photo 32 h	0.45±0.06	0.14±0.05	0.32±0.02
Soil Oa L Photo 64 h	0.38±0.07	0.09±0.01	0.29±0.07
Soil Oa L Photo 96 h	0.29±0.01	0.05±0.01	0.24±0.01
Sample Name	$\Phi_{app,\ ^3DOM^*_{TMP}}$ ($\times10^{-2}$ mol mol-photons^{-1})	$\Phi_{app,\ ^3DOM^*_{TMP},\ high\text{-}energy}$ ($\times10^{-2}$ mol mol-photons^{-1})	$\Phi_{app,\ ^3DOM^*_{TMP},\ low\text{-}energy}$ ($\times10^{-2}$ mol mol-photons^{-1})
Soil Oa L Bio 0 d	1.74±0.13	0.97±0.07	0.76±0.05
Soil Oa L Bio 8 d	3.17±0.11	1.92±0.03	1.25±0.08
Soil Oa L Bio 16 d	4.15±0.42	2.53±0.31	1.62±0.12
Soil Oa L Bio 32 d	4.32±0.36	2.70±0.24	1.62±0.12
Soil Oa L Photo-Bio 0 d	0.94±0.01	0.48±0.02	0.47±0.01
Soil Oa L Photo-Bio 8 d	1.43±0.06	0.79±0.04	0.64±0.03
Soil Oa L Photo-Bio 16 d	1.81±0.11	1.03±0.07	0.77±0.04
Soil Oa L Photo-Bio 32 d	2.15±0.09	1.29±0.06	0.87±0.03
Sample Name	$\Phi_{app,\ ^3DOM^*_{TMP}}$ ($\times10^{-2}$ mol mol-photons^{-1})	$\Phi_{app,\ ^3DOM^*_{TMP},\ high\text{-}energy}$ ($\times10^{-2}$ mol mol-photons^{-1})	$\Phi_{app,\ ^3DOM^*_{TMP},\ low\text{-}energy}$ ($\times10^{-2}$ mol mol-photons^{-1})
Soil Oa R Photo 0 h	1.74±0.08	0.97±0.05	0.77±0.03
Soil Oa R Photo 1 h	1.22±0.03	0.66±0.03	0.56±0.01
Soil Oa R Photo 2 h	1.09±0.07	0.57±0.02	0.52±0.05
Soil Oa R Photo 4 h	0.90±0.04	0.44±0.01	0.46±0.03
Soil Oa R Photo 8 h	0.76±0.03	0.33±0.06	0.42±0.03
Soil Oa R Photo 16 h	0.60±0.03	0.22±0.01	0.37±0.02
Soil Oa R Photo 32 h	0.52±0.02	0.16±0.04	0.37±0.02
Soil Oa R Photo 64 h	0.44±0.02	0.11±0.03	0.34±0.01
Soil Oa R Photo 96 h	0.37±0.02	0.07±0.03	0.30±0.01
Sample Name	$\Phi_{app,\ ^3DOM^*_{TMP}}$ ($\times10^{-2}$ mol mol-photons^{-1})	$\Phi_{app,\ ^3DOM^*_{TMP},\ high\text{-}energy}$ ($\times10^{-2}$ mol mol-photons^{-1})	$\Phi_{app,\ ^3DOM^*_{TMP},\ low\text{-}energy}$ ($\times10^{-2}$ mol mol-photons^{-1})
Soil Oa R Bio 0 d	1.77±0.14	0.98±0.09	0.79±0.05
Soil Oa R Bio 8 d	3.51±0.02	2.09±0.10	1.42±0.08
Soil Oa R Bio 16 d	4.34±0.31	2.61±0.19	1.74±0.12
Soil Oa R Bio 32 d	4.61±0.23	2.86±0.13	1.75±0.10
Soil Oa R Photo-Bio 0 d	1.09±0.07	0.58±0.03	0.51±0.04
Soil Oa R Photo-Bio 8 d	1.63±0.11	0.90±0.07	0.72±0.04
Soil Oa R Photo-Bio 16 d	1.87±0.14	1.08±0.07	0.80±0.07
Soil Oa R Photo-Bio 32 d	2.38±0.18	1.42±0.12	0.96±0.06

"Photo" = photodegradation; "Bio" = biodegradation; "Photo-Bio" = photo-biodegradation; "L" = the limed tributary watershed W16L; "R" = the reference tributary watershed W24R. Errors represent one standard deviation from duplicate measurements.

Table S11. $\Phi_{app, ^3DOM^*_{TMP}}$ for standardized leachates, whole water samples, and model DOM solutions (continued)

Sample Name	$\Phi_{app, ^3DOM^*_{TMP}}$ ($\times10^{-2}$ mol mol-photons^{-1})	$\Phi_{app, ^3DOM^*_{TMP}, high-energy}$ ($\times10^{-2}$ mol mol-photons^{-1})	$\Phi_{app, ^3DOM^*_{TMP}, low-energy}$ ($\times10^{-2}$ mol mol-photons^{-1})
Leaf Photo 0 h	1.57±0.13	0.82±0.06	0.75±0.07
Leaf Photo 1 h	1.06±0.07	0.53±0.06	0.53±0.02
Leaf Photo 2 h	0.85±0.05	0.42±0.05	0.43±0.01
Leaf Photo 4 h	0.76±0.07	0.35±0.06	0.40±0.01
Leaf Photo 8 h	0.61±0.02	0.26±0.04	0.36±0.02
Leaf Photo 16 h	0.55±0.05	0.20±0.06	0.35±0.01
Leaf Photo 32 h	0.46±0.01	0.13±0.04	0.33±0.03
Leaf Photo 64 h	0.38±0.01	0.09±0.03	0.29±0.01
Leaf Photo 96 h	0.30±0.02	0.06±0.02	0.24±0.03
Sample Name	$\Phi_{app, ^3DOM^*_{TMP}}$ ($\times10^{-2}$ mol mol-photons^{-1})	$\Phi_{app, ^3DOM^*_{TMP}, high-energy}$ ($\times10^{-2}$ mol mol-photons^{-1})	$\Phi_{app, ^3DOM^*_{TMP}, low-energy}$ ($\times10^{-2}$ mol mol-photons^{-1})
Leaf Bio 0 d	1.57±0.09	0.82±0.05	0.75±0.04
Leaf Bio 8 d	3.02±0.03	1.73±0.07	1.29±0.10
Leaf Bio 16 d	3.55±0.02	2.05±0.01	1.50±0.03
Leaf Bio 32 d	3.97±0.03	2.39±0.01	1.59±0.03
Leaf Photo-Bio 0 d	0.85±0.01	0.42±0.02	0.43±0.02
Leaf Photo-Bio 8 d	1.49±0.06	0.79±0.04	0.70±0.02
Leaf Photo-Bio 16 d	2.08±0.12	1.14±0.06	0.94±0.05
Leaf Photo-Bio 32 d	2.36±0.19	1.36±0.11	1.00±0.08
Sample Name	$\Phi_{app, ^3DOM^*_{TMP}}$ ($\times10^{-2}$ mol mol-photons^{-1})	$\Phi_{app, ^3DOM^*_{TMP}, high-energy}$ ($\times10^{-2}$ mol mol-photons^{-1})	$\Phi_{app, ^3DOM^*_{TMP}, low-energy}$ ($\times10^{-2}$ mol mol-photons^{-1})
Soil Oa L-L	1.74±0.16	0.97±0.21	0.76±0.06
Soil Bs L-L	7.06±0.73	4.35±1.04	2.70±0.30
Soil Oa L-M	1.76±0.11	0.99±0.17	0.77±0.06
Soil Bs L-M	6.86±0.85	4.11±1.06	2.75±0.21
Soil Oa L-H	1.74±0.13	0.98±0.19	0.75±0.06
Soil Bs L-H	7.20±0.68	4.50±0.85	2.70±0.17
Soil Oa R-L	1.78±0.11	0.99±0.17	0.80±0.06
Soil Bs R-L	7.13±0.49	4.32±0.69	2.80±0.19
Soil Oa R-M	1.78±0.11	0.99±0.17	0.78±0.06
Soil Bs R-M	7.02±0.49	4.16±0.22	2.87±0.26
Soil Oa R-H	1.77±0.14	0.98±0.20	0.79±0.06
Soil Bs R-H	6.84±0.52	4.04±0.85	2.80±0.32
Stream L	2.60±0.20	1.54±0.10	1.06±0.11
Stream R	2.70±0.25	1.58±0.14	1.12±0.11
Honnedaga Lake	2.24±0.16	1.38±0.10	0.86±0.07

"Photo" = photodegradation; "Bio" = biodegradation; "Photo-Bio" = photo-biodegradation; "L" = the limed tributary watershed W16L; "R" = the reference tributary watershed W24R. Errors represent one standard deviation from duplicate measurements.

**Table S11.** $\Phi_{app,\ ^3DOM^*_{TMP}}$ for standardized leachates, whole water samples, and model DOM solutions (continued)			
Sample Name	$\Phi_{app,\ ^3DOM^*_{TMP}}$ ($\times10^{-2}$ mol mol-photons^{-1})	$\Phi_{app,\ ^3DOM^*_{TMP},\ high\text{-}energy}$ ($\times10^{-2}$ mol mol-photons^{-1})	$\Phi_{app,\ ^3DOM^*_{TMP},\ low\text{-}energy}$ ($\times10^{-2}$ mol mol-photons^{-1})
SRFA	2.12±0.12	1.17±0.13	0.95±0.07
SRHA	1.55±0.07	0.32±0.07	1.24±0.01
SRNOM	2.04±0.08	0.93±0.12	1.11±0.03
PPFA	2.10±0.05	0.92±0.05	1.18±0.01
PPHA	1.92±0.05	0.55±0.01	1.38±0.06
NRNOM	2.26±0.18	1.15±0.20	1.11±0.02
UMRNOM	2.18±0.03	1.24±0.06	0.94±0.03
ESHA	1.53±0.02	0.40±0.11	1.13±0.13
Sample Name	$\Phi_{app,\ ^3DOM^*_{TMP}}$ ($\times10^{-2}$ mol mol-photons^{-1})	$\Phi_{app,\ ^3DOM^*_{TMP},\ high\text{-}energy}$ ($\times10^{-2}$ mol mol-photons^{-1})	$\Phi_{app,\ ^3DOM^*_{TMP},\ low\text{-}energy}$ ($\times10^{-2}$ mol mol-photons^{-1})
SRFA Photo 0 h	2.20±0.11	1.21±0.20	0.99±0.09
SRFA Photo 4 h	2.17±0.04	1.18±0.18	0.99±0.14
SRFA Photo 16 h	2.01±0.10	1.09±0.18	0.92±0.07
SRFA Photo 64 h	1.82±0.04	0.99±0.11	0.83±0.07
ESHA Photo 0 h	1.53±0.02	0.40±0.11	1.13±0.13
ESHA Photo 4 h	1.48±0.04	0.35±0.16	1.13±0.12
ESHA Photo 16 h	1.36±0.01	0.30±0.15	1.06±0.15
ESHA Photo 64 h	1.22±0.03	0.21±0.07	1.01±0.10
Glucose Bio 0 d	0.19±0.01	0.01±0.01	0.18±0.01
Glucose Bio 8 d	4.52±0.19	2.73±0.19	1.79±0.01
Glucose Bio 16 d	6.07±0.37	3.77±0.38	2.30±0.03
Glucose Bio 32 d	7.19±0.55	4.61±0.49	2.58±0.12
ESHA Bio 0 d	1.53±0.02	0.40±0.15	1.13±0.13
ESHA Bio 8 d	1.55±0.05	0.41±0.16	1.14±0.11
ESHA Bio 16 d	1.56±0.01	0.41±0.15	1.15±0.15
ESHA Bio 32 d	1.56±0.07	0.41±0.08	1.16±0.15

"Photo" = photodegradation; "Bio" = biodegradation; "Photo-Bio" = photo-biodegradation; "L" = the limed tributary watershed W16L; "R" = the reference tributary watershed W24R. Errors represent one standard deviation from duplicate measurements.

Table S12. $\%\Phi_{app,\ ^3DOM^*_{TMP},\ high\text{-}energy}$ and $\%\Phi_{app,\ ^3DOM^*_{TMP},\ low\text{-}energy}$ for standardized leachates, whole water samples, and model DOM solutions

Sample Name	$\%\Phi_{app,\ ^3DOM^*_{TMP},\ high\text{-}energy}$	$\%\Phi_{app,\ ^3DOM^*_{TMP},\ low\text{-}energy}$
Soil Oa L Photo 0 h	56.2±0.7	43.7±7.8
Soil Oa L Photo 1 h	53.2±1.5	46.8±6.4
Soil Oa L Photo 2 h	51.8±1.5	48.2±7.9
Soil Oa L Photo 4 h	48.4±3.9	51.7±7.1
Soil Oa L Photo 8 h	43.1±3.8	56.9±3.7
Soil Oa L Photo 16 h	36.5±1.0	63.5±8.0
Soil Oa L Photo 32 h	29.5±6.5	70.0±14.5
Soil Oa L Photo 64 h	23.9±5.9	76.7±26.8
Soil Oa L Photo 96 h	17.5±0.3	82.5±5.1
Sample Name	$\%\Phi_{app,\ ^3DOM^*_{TMP},\ high\text{-}energy}$	$\%\Phi_{app,\ ^3DOM^*_{TMP},\ low\text{-}energy}$
Soil Oa L Bio 0 d	56.1±0.1	43.9±7.9
Soil Oa L Bio 8 d	60.5±1.0	39.5±4.3
Soil Oa L Bio 16 d	60.8±1.2	39.1±10.5
Soil Oa L Bio 32 d	62.4±0.3	37.5±8.8
Soil Oa L Photo-Bio 0 d	50.4±1.3	49.6±1.3
Soil Oa L Photo-Bio 8 d	55.3±0.1	44.7±4.8
Soil Oa L Photo-Bio 16 d	57.2±0.6	42.8±6.5
Soil Oa L Photo-Bio 32 d	59.8±0.4	40.2±4.6
Sample Name	$\%\Phi_{app,\ ^3DOM^*_{TMP},\ high\text{-}energy}$	$\%\Phi_{app,\ ^3DOM^*_{TMP},\ low\text{-}energy}$
Soil Oa R Photo 0 h	55.6±0.5	44.4±4.7
Soil Oa R Photo 1 h	54.0±1.4	46.0±2.1
Soil Oa R Photo 2 h	52.7±1.7	47.3±8.0
Soil Oa R Photo 4 h	48.7±1.7	51.4±5.5
Soil Oa R Photo 8 h	44.0±6.0	55.9±5.3
Soil Oa R Photo 16 h	37.4±0.7	62.6±6.5
Soil Oa R Photo 32 h	29.6±6.8	70.2±5.8
Soil Oa R Photo 64 h	23.5±5.1	76.3±5.4
Soil Oa R Photo 96 h	17.8±6.4	82.0±6.5
Sample Name	$\%\Phi_{app,\ ^3DOM^*_{TMP},\ high\text{-}energy}$	$\%\Phi_{app,\ ^3DOM^*_{TMP},\ low\text{-}energy}$
Soil Oa R Bio 0 d	55.5±0.6	44.5±8.5
Soil Oa R Bio 8 d	59.5±2.5	40.5±2.3
Soil Oa R Bio 16 d	60.0±0.2	40.0±7.6
Soil Oa R Bio 32 d	62.1±0.2	37.9±5.4
Soil Oa R Photo-Bio 0 d	52.9±0.3	47.1±7.1
Soil Oa R Photo-Bio 8 d	55.6±0.5	44.4±7.0
Soil Oa R Photo-Bio 16 d	57.6±0.5	42.4±8.1
Soil Oa R Photo-Bio 32 d	59.8±0.4	40.1±8.0

"Photo" = photodegradation; "Bio" = biodegradation; "Photo-Bio" = photo-biodegradation; "L" = the limed tributary watershed W16L; "R" = the reference tributary watershed W24R. Errors represent one standard deviation from duplicate measurements.

**Table S12.** %$\Phi_{app,\ ^3DOM^*_{TMP},\ high\text{-}energy}$ and %$\Phi_{app,\ ^3DOM^*_{TMP},\ low\text{-}energy}$ for standardized leachates, whole water samples, and model DOM solutions (continued)		
Sample Name	%$\Phi_{app,\ ^3DOM^*_{TMP},\ high\text{-}energy}$	%$\Phi_{app,\ ^3DOM^*_{TMP},\ low\text{-}energy}$
Leaf Photo 0 h	52.1±0.5	48.0±9.7
Leaf Photo 1 h	50.0±2.0	49.9±7.2
Leaf Photo 2 h	48.9±2.9	51.0±6.2
Leaf Photo 4 h	46.3±3.8	53.5±9.8
Leaf Photo 8 h	41.7±5.3	58.2±4.6
Leaf Photo 16 h	36.4±7.2	63.2±9.5
Leaf Photo 32 h	28.2±8.9	71.7±7.8
Leaf Photo 64 h	23.1±6.5	76.8±5.1
Leaf Photo 96 h	18.7±6.3	81.5±12.3
Sample Name	%$\Phi_{app,\ ^3DOM^*_{TMP},\ high\text{-}energy}$	%$\Phi_{app,\ ^3DOM^*_{TMP},\ low\text{-}energy}$
Leaf Bio 0 d	52.0±0.1	48.0±6.4
Leaf Bio 8 d	57.3±2.9	42.7±3.5
Leaf Bio 16 d	57.7±0.6	42.3±1.1
Leaf Bio 32 d	60.1±0.4	39.9±1.0
Leaf Photo-Bio 0 d	49.0±2.4	51.0±2.5
Leaf Photo-Bio 8 d	52.8±0.5	47.1±4.6
Leaf Photo-Bio 16 d	54.9±0.1	45.2±6.3
Leaf Photo-Bio 32 d	57.6±0.1	42.4±8.8
Sample Name	%$\Phi_{app,\ ^3DOM^*_{TMP},\ high\text{-}energy}$	%$\Phi_{app,\ ^3DOM^*_{TMP},\ low\text{-}energy}$
Soil Oa L-L	55.7±7.3	43.9±9.7
Soil Bs L-L	61.3±8.3	38.3±11.3
Soil Oa L-M	56.2±6.2	43.6±7.2
Soil Bs L-M	59.4±8.1	40.1±12.8
Soil Oa L-H	56.4±6.6	43.4±8.0
Soil Bs L-H	62.2±6.0	37.5±9.7
Soil Oa R-L	55.1±6.2	44.7±7.1
Soil Bs R-L	60.5±5.4	39.3±7.4
Soil Oa R-M	55.6±6.1	44.2±7.1
Soil Bs R-M	59.2±0.9	40.8±7.9
Soil Oa R-H	54.9±7.1	44.8±8.7
Soil Bs R-H	58.7±7.9	41.0±9.0
Stream L	59.2±0.9	40.9±8.9
Stream R	58.6±0.2	41.4±10.1
Honnedaga Lake	61.5±0.2	38.5±7.9

"Photo" = photodegradation; "Bio" = biodegradation; "Photo-Bio" = photo-biodegradation; "L" = the limed tributary watershed W16L; "R" = the reference tributary watershed W24R. Errors represent one standard deviation from duplicate measurements.

Table S12. %$\Phi_{app,\ ^3DOM^*_{TMP},\ high\text{-}energy}$ and %$\Phi_{app,\ ^3DOM^*_{TMP},\ low\text{-}energy}$ for standardized leachates, whole water samples, and model DOM solutions (continued)

Sample Name	%$\Phi_{app,\ ^3DOM^*_{TMP},\ high\text{-}energy}$	%$\Phi_{app,\ ^3DOM^*_{TMP},\ low\text{-}energy}$
SRFA	55.1±2.6	44.8±2.7
SRHA	20.5±3.4	79.5±4.2
SRNOM	45.7±3.8	54.3±4.4
PPFA	43.9±1.5	56.0±2.3
PPHA	28.4±1.3	71.7±4.4
NRNOM	50.9±4.9	48.9±7.8
UMRNOM	56.8±1.9	43.2±1.9
ESHA	25.9±7.0	74.1±9.2
Sample Name	%$\Phi_{app,\ ^3DOM^*_{TMP},\ high\text{-}energy}$	%$\Phi_{app,\ ^3DOM^*_{TMP},\ low\text{-}energy}$
SRFA Photo 0 h	54.8±6.3	45.1±6.4
SRFA Photo 4 h	54.4±7.3	45.5±6.7
SRFA Photo 16 h	54.1±6.0	45.8±6.3
SRFA Photo 64 h	54.1±5.1	45.8±4.6
ESHA Photo 0 h	26.0±7.7	74.1±8.8
ESHA Photo 4 h	23.7±10	76.1±8.4
ESHA Photo 16 h	22.1±11	77.9±11.2
ESHA Photo 64 h	17.6±6.1	82.5±8.7
Glucose Bio 0 d	5.4±4.1	94.6±11.7
Glucose Bio 8 d	60.4±1.7	39.6±0.3
Glucose Bio 16 d	62.1±2.3	37.9±3.0
Glucose Bio 32 d	64.0±2.3	35.9±3.5
ESHA Bio 0 d	25.9±9.4	74.0±8.6
ESHA Bio 8 d	26.0±9.4	73.8±7.7
ESHA Bio 16 d	26.1±9.8	73.9±9.6
ESHA Bio 32 d	26.2±6.1	73.9±10.4

"Photo" = photodegradation; "Bio" = biodegradation; "Photo-Bio" = photo-biodegradation; "L" = the limed tributary watershed W16L; "R" = the reference tributary watershed W24R. Errors represent one standard deviation from duplicate measurements.

9. *trans,trans*-2,4-Hexadien-1-ol (*t,t*-HDO) as an energy transfer probe for $^3DOM^*$

t,t-HDO (i.e., sorbic alcohol) was spiked into samples to measure the photoproduction of $^3DOM^*_{HDO}$. For each standardized sample, the formation of three *t,t*-HDO isomers (i.e., *c,c*-HDO, *c,t*-HDO, and *t,c*-HDO) and the loss of *t,t*-HDO were monitored to derive the overall production rate of four isomers, $R_{prod,\ HDO}$ (M s^{-1}), for each initial *t,t*-HDO concentration:[42, 102]

$$\begin{aligned} R_{prod,\ HDO} &= \frac{d[HDO]}{dt} = R_{f,\ c,t\text{-HDO}} + R_{f,\ c,c\text{-HDO}} + R_{f,\ t,c\text{-HDO}} + R_{f,\ t,t\text{-HDO}} \\ &= \frac{d[c,t\text{-HDO}]}{dt} + \frac{d[c,c\text{-HDO}]}{dt} + \frac{d[t,c\text{-HDO}]}{dt} + \frac{d[t,t\text{-HDO}]}{dt} \end{aligned} \quad (S23)$$

where $R_{f,\ c,t\text{-HDO}}$ (M s^{-1}) is the formation rate of *c,t*-HDO, $R_{f,\ c,c\text{-HDO}}$ (M s^{-1}) is the formation rate of *c,c*-HDO, $R_{f,\ t,c\text{-HDO}}$ (M s^{-1}) is the formation rate of *t,c*-HDO, and $R_{f,\ t,t\text{-HDO}}$ (M s^{-1}) is the *reformation* rate of *t,t*-HDO upon the relaxation of the excited triplet state of *t,t*-HDO (i.e., *t,t*-HDO*).

To determine the rate constant for *t,t*-HDO reformation relative to that of *c,t*-HDO, a multiple linear regression of the *t,t*-HDO and *c,t*-HDO data in the initial rate kinetics regime was performed using Equation S24 (i.e., $y = ax_1 + bx_2$ where y = d[*t,t*-HDO]/dt, x_1 = d[*c,t*-HDO]/dt, and x_2 = [*t,t*-HDO]):[42, 102]

$$\frac{d[t,t\text{-HDO}]}{dt} = \frac{k_{t,t\text{-HDO}}}{k_{c,t\text{-HDO}}} \frac{d[c,t\text{-HDO}]}{dt} - k'_{t,t\text{-HDO}}[t,t\text{-HDO}] \quad (S24)$$

where $k_{t,t\text{-HDO}}$ (s^{-1}) is the pseudo-first order rate constant for *t,t*-HDO reformation from *t,t*-HDO*, $k_{c,t\text{-HDO}}$ (s^{-1}) is the pseudo-first order rate constant for *c,t*-HDO formation from *t,t*-HDO*, $k'_{t,t\text{-HDO}}$ (s^{-1}) is the sum of pseudo-first-order rate constants for the reaction of *t,t*-HDO with $^3DOM^*$ to form *t,t*-HDO* and reactions of *t,t*-HDO with other scavengers under steady-state conditions (e.g., $^\bullet OH$ and $^1O_2$[42]), and [*t,t*-HDO] is the initial concentration of *t,t*-HDO.

To approximate the sample-specific *t,t*-HDO reformation, a representative relative rate constant (i.e., $k_{t,t\text{-HDO}}/k_{c,t\text{-HDO}}$) of 2.68±0.17 was derived by regressing Equation S24 with data from all leachate and water samples. This relative rate constant agreed well with the value (2.74±0.36) measured for surface water samples collected from Adirondack lakes in our previous work.[4]

For each standardized sample, the steady-state concentration of $^3DOM^*$ in the *presence* of *t,t*-HDO, $[^3DOM^*_{HDO}]_{ss}$, was calculated by considering the formation of $^3DOM^*_{HDO}$ and the simultaneous quenching by *t,t*-HDO and other deactivation processes:[42]

$$[^3DOM^*_{HDO}]_{ss} = \frac{R_{f,\,^3DOM^*_{HDO}}}{k_{t,t\text{-HDO},\,^3DOM^*_{HDO}}[t,t\text{-HDO}] + k'_{q,\,^3DOM^*_{HDO}}} \quad \text{(S25)}$$

where $R_{f,\,^3DOM^*_{HDO}}$ (M s^{-1}) is the formation rate of $^3DOM^*_{HDO}$, $k_{t,t\text{-HDO},\,^3DOM^*_{HDO}}$ (M^{-1} s^{-1}) is the second-order reaction rate constant of *t,t*-HDO with $^3DOM^*_{HDO}$, and $k'_{q,\,^3DOM^*_{HDO}}$ ($3.2(\pm0.6)\times10^5$ s^{-1}) is the sum of pseudo-first order rate constants for $^3DOM^*_{HDO}$ quenching via energy transfer to dissolved O_2 ($2.1(\pm0.2)\times10^5$ s^{-1} calculated from $k_{O_2}[O_{2(aq)}]$ where $k_{O_2} = 8.9(\pm0.6)\times10^8$ M^{-1} s^{-1} and $[O_{2(aq)}]$ = ~258 μM at T = 25 °C[98]) and via other non-O_2 dependent nonradiative relaxation pathways ($k_d^T = 9.0(\pm2.8)\times10^4$ s^{-1}).[79, 90]

To solve for $k_{t,t\text{-HDO},\,^3DOM^*_{HDO}}$ (**Table S13**), a linear regression of [*t,t*-HDO] (at varying concentrations of 50, 100, 250, 500, 1000, and 2000 μM) and $R_{p,\,HDO}$ data was performed with the linearized form of Equation S26 (i.e., $y = ax + b$ where $y = [t,t\text{-HDO}]/R_{p,\,HDO}$ and $x = [t,t\text{-HDO}]$):[42]

$$\frac{[t,t\text{-HDO}]}{R_{p,\,HDO}} = \frac{[t,t\text{-HDO}]}{R_{f,\,^3DOM^*_{HDO}}} + \frac{k'_{q,\,^3DOM^*_{HDO}}}{R_{f,\,^3DOM^*_{HDO}}\,k_{t,t\text{-HDO},\,^3DOM^*_{HDO}}} \quad \text{(S26)}$$

The formation rate of $^3DOM^*_{HDO}$, $R_{f,\,^3DOM^*_{HDO}}$ (M s^{-1}), was calculated as:

$$R_{f,\,^3DOM^*_{HDO}} = \frac{1}{\text{slope}} \quad \text{(S27)}$$

For each standardized sample, the steady-state concentration of $^3DOM^*$ in the *absence* of *t,t*-HDO, $[^3DOM^*_{HDO}]_{ss}$, was calculated as:

$$[^3DOM^*_{HDO}]_{ss} = \frac{R_{f,\,^3DOM^*_{HDO}}}{k'_{q,\,^3DOM^*_{HDO}}} = \frac{1}{k'_{q,\,^3DOM^*_{HDO}}}\frac{1}{\text{slope}} \quad \text{(S28)}$$

The apparent quantum yield of $^3DOM^*_{HDO}$, $\Phi_{app,\,^3DOM^*_{HDO}}$ (mol mol-photons^{-1}), was calculated as:[42]

$$\Phi_{\mathrm{app,\ ^3DOM^*_{HDO}}} = \frac{R_{\mathrm{f,\ ^3DOM^*_{HDO}}}}{R_\mathrm{a}} = \frac{1}{R_\mathrm{a}}\frac{1}{\mathrm{slope}} \tag{S29}$$

The yield of $^1O_2$ the O_2-dependent quenching of $^3DOM^*_{HDO}$ were calculated as:[79]

$$f_{\mathrm{^1O_2\_^3DOM^*_{HDO}}} = \frac{\Phi_{\mathrm{app,\ ^1O_2}}(k_{\mathrm{O_2}}[\mathrm{O_{2(aq)}}] + k_\mathrm{d}^\mathrm{T})}{\Phi_{\mathrm{app,\ ^3DOM^*_{HDO}}} k_{\mathrm{O_2}}[\mathrm{O_{2(aq)}}]} \tag{S30}$$

$\Phi_{\mathrm{app,\ ^3DOM^*_{HDO}}}$ are summarized in **Table S14**. $f_{\mathrm{^1O_2\_^3DOM^*_{HDO}}}$ are summarized in **Table S15**.

Table S13. $k_{t,t\text{-HDO},\,^3\text{DOM}^*_\text{HDO}}$ for standardized leachates, whole water samples, and model DOM solutions

Sample Name	$k_{t,t\text{-HDO},\,^3\text{DOM}^*_\text{HDO}}$ ($\times 10^8$ M^{-1} s^{-1})	Sample Name	$k_{t,t\text{-HDO},\,^3\text{DOM}^*_\text{HDO}}$ ($\times 10^8$ M^{-1} s^{-1})
Soil Oa L Photo 0 h	8.82±1.39	Soil Oa L Bio 0 d	8.83±1.47
Soil Oa L Photo 1 h	8.43±1.32	Soil Oa L Bio 8 d	8.94±1.40
Soil Oa L Photo 2 h	8.34±1.31	Soil Oa L Bio 16 d	9.09±1.44
Soil Oa L Photo 4 h	8.31±1.52	Soil Oa L Bio 32 d	9.22±1.63
Soil Oa L Photo 8 h	8.27±1.31	Soil Oa L Photo-Bio 0 d	8.34±1.39
Soil Oa L Photo 16 h	8.25±1.31	Soil Oa L Photo-Bio 8 d	8.55±1.47
Soil Oa L Photo 32 h	8.06±1.36	Soil Oa L Photo-Bio 16 d	8.71±1.37
Soil Oa L Photo 64 h	8.00±1.32	Soil Oa L Photo-Bio 32 d	8.96±1.41
Soil Oa L Photo 96 h	7.92±1.24		
Sample Name	$k_{t,t\text{-HDO},\,^3\text{DOM}^*_\text{HDO}}$ ($\times 10^8$ M^{-1} s^{-1})	Sample Name	$k_{t,t\text{-HDO},\,^3\text{DOM}^*_\text{HDO}}$ ($\times 10^8$ M^{-1} s^{-1})
Soil Oa R Photo 0 h	8.91±1.73	Soil Oa R Bio 0 d	8.93±1.41
Soil Oa R Photo 1 h	8.55±1.47	Soil Oa R Bio 8 d	9.03±1.47
Soil Oa R Photo 2 h	8.46±1.35	Soil Oa R Bio 16 d	9.13±1.82
Soil Oa R Photo 4 h	8.45±1.35	Soil Oa R Bio 32 d	9.28±1.56
Soil Oa R Photo 8 h	8.18±1.44	Soil Oa R Photo-Bio 0 d	8.45±1.33
Soil Oa R Photo 16 h	8.14±1.37	Soil Oa R Photo-Bio 8 d	8.61±1.41
Soil Oa R Photo 32 h	8.08±1.28	Soil Oa R Photo-Bio 16 d	8.79±1.61
Soil Oa R Photo 64 h	8.03±1.44	Soil Oa R Photo-Bio 32 d	9.15±1.48
Soil Oa R Photo 96 h	7.97±1.30		
Sample Name	$k_{t,t\text{-HDO},\,^3\text{DOM}^*_\text{HDO}}$ ($\times 10^8$ M^{-1} s^{-1})	Sample Name	$k_{t,t\text{-HDO},\,^3\text{DOM}^*_\text{HDO}}$ ($\times 10^8$ M^{-1} s^{-1})
Leaf Photo 0 h	8.94±1.84	Leaf Bio 0 d	8.93±1.43
Leaf Photo 1 h	8.92±1.55	Leaf Bio 8 d	9.04±1.58
Leaf Photo 2 h	8.89±1.63	Leaf Bio 16 d	9.18±1.64
Leaf Photo 4 h	8.82±1.40	Leaf Bio 32 d	9.27±2.11
Leaf Photo 8 h	8.68±1.52	Leaf Photo-Bio 0 d	8.90±1.90
Leaf Photo 16 h	8.59±1.50	Leaf Photo-Bio 8 d	8.97±1.57
Leaf Photo 32 h	8.54±1.54	Leaf Photo-Bio 16 d	9.10±1.82
Leaf Photo 64 h	8.48±1.34	Leaf Photo-Bio 32 d	9.49±1.76
Leaf Photo 96 h	8.27±1.32		

"Photo" = photodegradation; "Bio" = biodegradation; "Photo-Bio" = photo-biodegradation; "L" = the limed tributary watershed W16L; "R" = the reference tributary watershed W24R. Errors represent one standard deviation from duplicate measurements.

**Table S13.** $k_{t,t\text{-HDO},\,^3\text{DOM}^*_\text{HDO}}$ for standardized leachates, whole water samples, and model DOM solutions (continued)			
Sample Name	$k_{t,t\text{-HDO},\,^3\text{DOM}^*_\text{HDO}}$ (×10^8 M^{-1} s^{-1})	Sample Name	$k_{t,t\text{-HDO},\,^3\text{DOM}^*_\text{HDO}}$ (×10^8 M^{-1} s^{-1})
Soil Oa L-L	8.87±1.44	SRFA	7.75±1.25
Soil Bs L-L	9.29±1.47	SRHA	6.78±1.08
Soil Oa L-M	8.80±1.38	SRNOM	7.21±1.18
Soil Bs L-M	9.13±1.43	PPFA	7.98±1.27
Soil Oa L-H	8.94±1.60	PPHA	6.94±1.23
Soil Bs L-H	9.23±1.47	NRNOM	8.18±1.43
Soil Oa R-L	8.87±1.43	UMRNOM	8.31±1.31
Soil Bs R-L	9.20±1.47	ESHA	8.87±1.46
Soil Oa R-M	9.15±1.51		
Soil Bs R-M	9.50±1.53		
Soil Oa R-H	8.85±1.39		
Soil Bs R-H	9.23±1.46		
Stream L	8.27±1.32		
Stream R	8.62±1.41		
Honnedaga Lake	7.84±1.23		
Sample Name	$k_{t,t\text{-HDO},\,^3\text{DOM}^*_\text{HDO}}$ (×10^8 M^{-1} s^{-1})	Sample Name	$k_{t,t\text{-HDO},\,^3\text{DOM}^*_\text{HDO}}$ (×10^8 M^{-1} s^{-1})
Glucose Bio 0 d	0.02±0.01	ESHA Bio 0 d	8.87±1.46
Glucose Bio 8 d	2.14±0.33	ESHA Bio 8 d	8.11±1.28
Glucose Bio 16 d	2.00±0.33	ESHA Bio 16 d	8.11±1.27
Glucose Bio 32 d	1.92±0.29	ESHA Bio 32 d	8.05±1.27
"Photo" = photodegradation; "Bio" = biodegradation; "Photo-Bio" = photo-biodegradation; "L" = the limed tributary watershed W16L; "R" = the reference tributary watershed W24R. Errors represent one standard deviation from duplicate measurements.			

Table S14. $\Phi_{app,\ ^3DOM^*_{HDO}}$ for standardized leachates, whole water samples, and model DOM solutions

Sample Name	$\Phi_{app,\ ^3DOM^*_{HDO}}$ ($\times10^{-2}$ mol mol-photons^{-1})	Sample Name	$\Phi_{app,\ ^3DOM^*_{HDO}}$ ($\times10^{-2}$ mol mol-photons^{-1})
Soil Oa L Photo 0 h	0.59±0.01	Soil Oa L Bio 0 d	0.58±0.02
Soil Oa L Photo 1 h	0.58±0.01	Soil Oa L Bio 8 d	0.60±0.01
Soil Oa L Photo 2 h	0.58±0.02	Soil Oa L Bio 16 d	0.62±0.01
Soil Oa L Photo 4 h	0.58±0.03	Soil Oa L Bio 32 d	0.63±0.03
Soil Oa L Photo 8 h	0.57±0.03	Soil Oa L Photo-Bio 0 d	0.58±0.04
Soil Oa L Photo 16 h	0.58±0.09	Soil Oa L Photo-Bio 8 d	0.59±0.01
Soil Oa L Photo 32 h	0.57±0.08	Soil Oa L Photo-Bio 16 d	0.60±0.02
Soil Oa L Photo 64 h	0.57±0.09	Soil Oa L Photo-Bio 32 d	0.61±0.03
Soil Oa L Photo 96 h	0.57±0.10		
Sample Name	$\Phi_{app,\ ^3DOM^*_{HDO}}$ ($\times10^{-2}$ mol mol-photons^{-1})	Sample Name	$\Phi_{app,\ ^3DOM^*_{HDO}}$ ($\times10^{-2}$ mol mol-photons^{-1})
Soil Oa R Photo 0 h	0.59±0.04	Soil Oa R Bio 0 d	0.61±0.01
Soil Oa R Photo 1 h	0.60±0.05	Soil Oa R Bio 8 d	0.63±0.01
Soil Oa R Photo 2 h	0.60±0.01	Soil Oa R Bio 16 d	0.64±0.04
Soil Oa R Photo 4 h	0.59±0.01	Soil Oa R Bio 32 d	0.65±0.05
Soil Oa R Photo 8 h	0.59±0.02	Soil Oa R Photo-Bio 0 d	0.60±0.01
Soil Oa R Photo 16 h	0.59±0.04	Soil Oa R Photo-Bio 8 d	0.61±0.01
Soil Oa R Photo 32 h	0.59±0.02	Soil Oa R Photo-Bio 16 d	0.62±0.03
Soil Oa R Photo 64 h	0.58±0.04	Soil Oa R Photo-Bio 32 d	0.63±0.01
Soil Oa R Photo 96 h	0.59±0.01		
Sample Name	$\Phi_{app,\ ^3DOM^*_{HDO}}$ ($\times10^{-2}$ mol mol-photons^{-1})	Sample Name	$\Phi_{app,\ ^3DOM^*_{HDO}}$ ($\times10^{-2}$ mol mol-photons^{-1})
Leaf Photo 0 h	0.54±0.03	Leaf Bio 0 d	0.54±0.01
Leaf Photo 1 h	0.53±0.01	Leaf Bio 8 d	0.56±0.02
Leaf Photo 2 h	0.53±0.07	Leaf Bio 16 d	0.57±0.02
Leaf Photo 4 h	0.53±0.06	Leaf Bio 32 d	0.58±0.02
Leaf Photo 8 h	0.52±0.03	Leaf Photo-Bio 0 d	0.53±0.04
Leaf Photo 16 h	0.52±0.07	Leaf Photo-Bio 8 d	0.56±0.01
Leaf Photo 32 h	0.52±0.01	Leaf Photo-Bio 16 d	0.56±0.05
Leaf Photo 64 h	0.51±0.01	Leaf Photo-Bio 32 d	0.57±0.05
Leaf Photo 96 h	0.51±0.01		

"Photo" = photodegradation; "Bio" = biodegradation; "Photo-Bio" = photo-biodegradation; "L" = the limed tributary watershed W16L; "R" = the reference tributary watershed W24R. Errors represent one standard deviation from duplicate measurements.

**Table S14.** $\Phi_{app,\ ^3DOM^*_{HDO}}$ for standardized leachates, whole water samples, and model DOM solutions (continued)			
Sample Name	$\Phi_{app,\ ^3DOM^*_{HDO}}$ ($\times 10^{-2}$ mol mol-photons^{-1})	Sample Name	$\Phi_{app,\ ^3DOM^*_{HDO}}$ ($\times 10^{-2}$ mol mol-photons^{-1})
Soil Oa L-L	0.58±0.01	SRFA	1.71±0.05
Soil Bs L-L	1.93±0.01	SRHA	0.64±0.06
Soil Oa L-M	0.59±0.01	SRNOM	1.57±0.08
Soil Bs L-M	1.92±0.05	PPFA	1.68±0.06
Soil Oa L-H	0.58±0.03	PPHA	0.45±0.01
Soil Bs L-H	1.94±0.01	NRNOM	1.89±0.04
Soil Oa R-L	0.61±0.01	UMRNOM	1.96±0.03
Soil Bs R-L	1.98±0.01	ESHA	0.44±0.02
Soil Oa R-M	0.61±0.01		
Soil Bs R-M	1.95±0.01		
Soil Oa R-H	0.61±0.01		
Soil Bs R-H	1.93±0.06		
Stream L	0.89±0.02		
Stream R	0.89±0.02		
Honnedaga Lake	0.72±0.01		
Sample Name	$\Phi_{app,\ ^3DOM^*_{HDO}}$ ($\times 10^{-2}$ mol mol-photons^{-1})	Sample Name	$\Phi_{app,\ ^3DOM^*_{HDO}}$ ($\times 10^{-2}$ mol mol-photons^{-1})
Glucose Bio 0 d	0.19±0.01	ESHA Bio 0 d	0.44±0.02
Glucose Bio 8 d	1.96±0.20	ESHA Bio 8 d	0.46±0.02
Glucose Bio 16 d	1.82±0.16	ESHA Bio 16 d	0.46±0.02
Glucose Bio 32 d	1.93±0.21	ESHA Bio 32 d	0.46±0.02

"Photo" = photodegradation; "Bio" = biodegradation; "Photo-Bio" = photo-biodegradation; "L" = the limed tributary watershed W16L; "R" = the reference tributary watershed W24R. Errors represent one standard deviation from duplicate measurements.

Table S15. $f_{{}^1O_2\_{}^3DOM^*_{HDO}}$ for standardized leachates, whole water samples, and model DOM solutions

Sample Name	$f_{{}^1O_2\_{}^3DOM^*_{HDO}}$	Sample Name	$f_{{}^1O_2\_{}^3DOM^*_{HDO}}$
Soil Oa L Photo 0 h	0.99±0.04	Soil Oa L Bio 0 d	0.99±0.04
Soil Oa L Photo 1 h	1.00±0.03	Soil Oa L Bio 8 d	0.99±0.02
Soil Oa L Photo 2 h	1.00±0.04	Soil Oa L Bio 16 d	0.99±0.02
Soil Oa L Photo 4 h	1.00±0.07	Soil Oa L Bio 32 d	0.99±0.06
Soil Oa L Photo 8 h	0.99±0.07	Soil Oa L Photo-Bio 0 d	1.00±0.10
Soil Oa L Photo 16 h	1.00±0.21	Soil Oa L Photo-Bio 8 d	1.00±0.01
Soil Oa L Photo 32 h	1.00±0.19	Soil Oa L Photo-Bio 16 d	1.00±0.04
Soil Oa L Photo 64 h	1.00±0.22	Soil Oa L Photo-Bio 32 d	1.00±0.08
Soil Oa L Photo 96 h	1.00±0.24		
Sample Name	$f_{{}^1O_2\_{}^3DOM^*_{HDO}}$	Sample Name	$f_{{}^1O_2\_{}^3DOM^*_{HDO}}$
Soil Oa R Photo 0 h	0.98±0.10	Soil Oa R Bio 0 d	0.98±0.01
Soil Oa R Photo 1 h	0.99±0.11	Soil Oa R Bio 8 d	0.97±0.02
Soil Oa R Photo 2 h	0.99±0.01	Soil Oa R Bio 16 d	0.99±0.10
Soil Oa R Photo 4 h	0.99±0.01	Soil Oa R Bio 32 d	0.99±0.11
Soil Oa R Photo 8 h	0.99±0.05	Soil Oa R Photo-Bio 0 d	0.99±0.01
Soil Oa R Photo 16 h	0.99±0.09	Soil Oa R Photo-Bio 8 d	0.99±0.01
Soil Oa R Photo 32 h	0.99±0.05	Soil Oa R Photo-Bio 16 d	1.00±0.07
Soil Oa R Photo 64 h	0.99±0.10	Soil Oa R Photo-Bio 32 d	0.99±0.01
Soil Oa R Photo 96 h	0.98±0.01		
Sample Name	$f_{{}^1O_2\_{}^3DOM^*_{HDO}}$	Sample Name	$f_{{}^1O_2\_{}^3DOM^*_{HDO}}$
Leaf Photo 0 h	1.00±0.08	Leaf Bio 0 d	0.99±0.01
Leaf Photo 1 h	1.00±0.01	Leaf Bio 8 d	1.00±0.06
Leaf Photo 2 h	1.00±0.20	Leaf Bio 16 d	1.00±0.06
Leaf Photo 4 h	0.99±0.17	Leaf Bio 32 d	0.99±0.04
Leaf Photo 8 h	0.97±0.07	Leaf Photo-Bio 0 d	0.99±0.11
Leaf Photo 16 h	0.98±0.18	Leaf Photo-Bio 8 d	0.98±0.01
Leaf Photo 32 h	0.96±0.02	Leaf Photo-Bio 16 d	1.00±0.12
Leaf Photo 64 h	0.98±0.04	Leaf Photo-Bio 32 d	0.99±0.12
Leaf Photo 96 h	0.98±0.02		

"Photo" = photodegradation; "Bio" = biodegradation; "Photo-Bio" = photo-biodegradation; "L" = the limed tributary watershed W16L; "R" = the reference tributary watershed W24R. Errors represent one standard deviation from duplicate measurements.

Table S15. $f_{^1O_2\_^3DOM^*_{HDO}}$ for standardized leachates, whole water samples, and model DOM solutions (continued)

Sample Name	$f_{^1O_2\_^3DOM^*_{HDO}}$	Sample Name	$f_{^1O_2\_^3DOM^*_{HDO}}$
Soil Oa L-L	0.98±0.01	SRFA	0.93±0.04
Soil Bs L-L	0.98±0.01	SRHA	0.98±0.13
Soil Oa L-M	0.99±0.01	SRNOM	0.92±0.07
Soil Bs L-M	0.95±0.03	PPFA	0.95±0.05
Soil Oa L-H	0.97±0.07	PPHA	0.99±0.02
Soil Bs L-H	1.00±0.01	NRNOM	0.94±0.03
Soil Oa R-L	0.98±0.01	UMRNOM	0.93±0.02
Soil Bs R-L	0.98±0.01	ESHA	1.00±0.05
Soil Oa R-M	0.98±0.01		
Soil Bs R-M	0.98±0.01		
Soil Oa R-H	0.94±0.02		
Soil Bs R-H	0.97±0.05		
Stream L	0.99±0.04		
Stream R	1.00±0.03		
Honnedaga Lake	0.99±0.02		
**Sample Name**	$f_{^1O_2\_^3DOM^*_{HDO}}$	**Sample Name**	$f_{^1O_2\_^3DOM^*_{HDO}}$
Glucose Bio 0 d	0.93±0.04	ESHA Bio 0 d	1.00±0.05
Glucose Bio 8 d	0.94±0.13	ESHA Bio 8 d	1.00±0.07
Glucose Bio 16 d	0.94±0.19	ESHA Bio 16 d	0.99±0.05
Glucose Bio 32 d	0.96±0.24	ESHA Bio 32 d	0.96±0.05

"Photo" = photodegradation; "Bio" = biodegradation; "Photo-Bio" = photo-biodegradation; "L" = the limed tributary watershed W16L; "R" = the reference tributary watershed W24R. Errors represent one standard deviation from duplicate measurements.

10. $\Phi_{app,RI}$ profile fittings during irradiation or incubation of leaf and soil Oa DOM

$\Phi_{app,RI}$ profiles measured over the course of photodegradation and (photo-)biodegradation experiments were fitted to a reactivity continuum model assuming a gamma distribution as initial reactivity distribution (**Figure S5**):[103-106]

$$\frac{(\Phi_{app,RI})_t}{(\Phi_{app,RI})_0} = \left(\frac{\alpha}{\alpha + t}\right)^{\pm\nu} \quad \text{(S31)}$$

where $(\Phi_{app,RI})_t$ (mol mol-photons^{-1}) is the $\Phi_{app,RI}$ measured at time t, $(\Phi_{app,RI})_0$ (mol mol-photons^{-1}) is the $\Phi_{app,RI}$ measured at $t = 0$, t (h or d) is the irradiation or incubation time, α (h or d) is a rate parameter,[103] and ν is a unitless shape parameter ("+" for describing the decay curves and "–" for describing the growth curves).[103] The gamma reactivity continuum model differs from discrete (multi)exponential models[107] in that it treats DOM reactivity as a continuous variable and is parsimonious with two fit parameters.[108] Profile fittings were performed in *GraphPad Prism 8.4.* using nonlinear least squares regression with 1000 allowable iterations at a tolerance level of 1×10^{-6}.

For each $\Phi_{app,RI}$ profile, the initial apparent first-order decay or growth coefficient $k_{0,\Phi_{app,RI}}$ (h^{-1} or d^{-1}) was calculated as:

$$k_{0,\Phi_{app,RI}} = \frac{\nu}{\alpha} \quad \text{(S32)}$$

Model parameters are summarized in **Table S16**.

**Table S16.** Model parameters of $\Phi_{app,RI}$ profile fittings by the reactivity continuum model					
Photodegradation	Rate Parameter α (h)	Shape Parameter ν (unitless)	Adjusted R^2	AICc	Initial Apparent First-Order Decay Coefficient $k_{0,\Phi_{app,RI}}$ (h^{-1})
$\Phi_{app,\ ^1O_2}$ ($\times 10^{-2}$ mol mol-photons^{-1})	0.12±0.01	0.14±0.01	0.950	-386.1	1.18±0.19
$\Phi_{app,\ ^3DOM^*_{TMP}}$ ($\times 10^{-2}$ mol mol-photons^{-1})	0.26±0.05	0.27±0.01	0.972	-372.6	1.06±0.16
$\Phi_{app,\ ^{\bullet}OH}$ ($\times 10^{-5}$ mol mol-photons^{-1})	0.20±0.10	0.14±0.01	0.814	-271.4	0.81±0.39
Biodegradation	Rate Parameter α (d)	Shape Parameter ν (unitless)	Adjusted R^2	AICc	Initial Apparent First-Order Growth Coefficient $k_{0,\Phi_{app,RI}}$ (d^{-1})
$\Phi_{app,\ ^1O_2}$ ($\times 10^{-2}$ mol mol-photons^{-1})	0.54±0.05	0.19±0.01	0.928	-55.9	0.36±0.03
$\Phi_{app,\ ^3DOM^*_{TMP}}$ ($\times 10^{-2}$ mol mol-photons^{-1})	0.29±0.03	0.20±0.02	0.946	-45.5	0.68±0.01
Photo-Biodegradation	Rate Parameter α (d)	Shape Parameter ν (unitless)	Adjusted R^2	AICc	Initial Apparent First-Order Growth Coefficient $k_{0,\Phi_{app,RI}}$ (d^{-1})
$\Phi_{app,\ ^1O_2}$ ($\times 10^{-2}$ mol mol-photons^{-1})	3.88±0.03	0.29±0.01	0.938	-59.3	0.07±0.01
$\Phi_{app,\ ^3DOM^*_{TMP}}$ ($\times 10^{-2}$ mol mol-photons^{-1})	2.59±0.18	0.34±0.01	0.946	-50.7	0.13±0.01
"AICc" = Akaike's corrected information criterion. Errors represent the 95% confidence intervals of model parameters.					

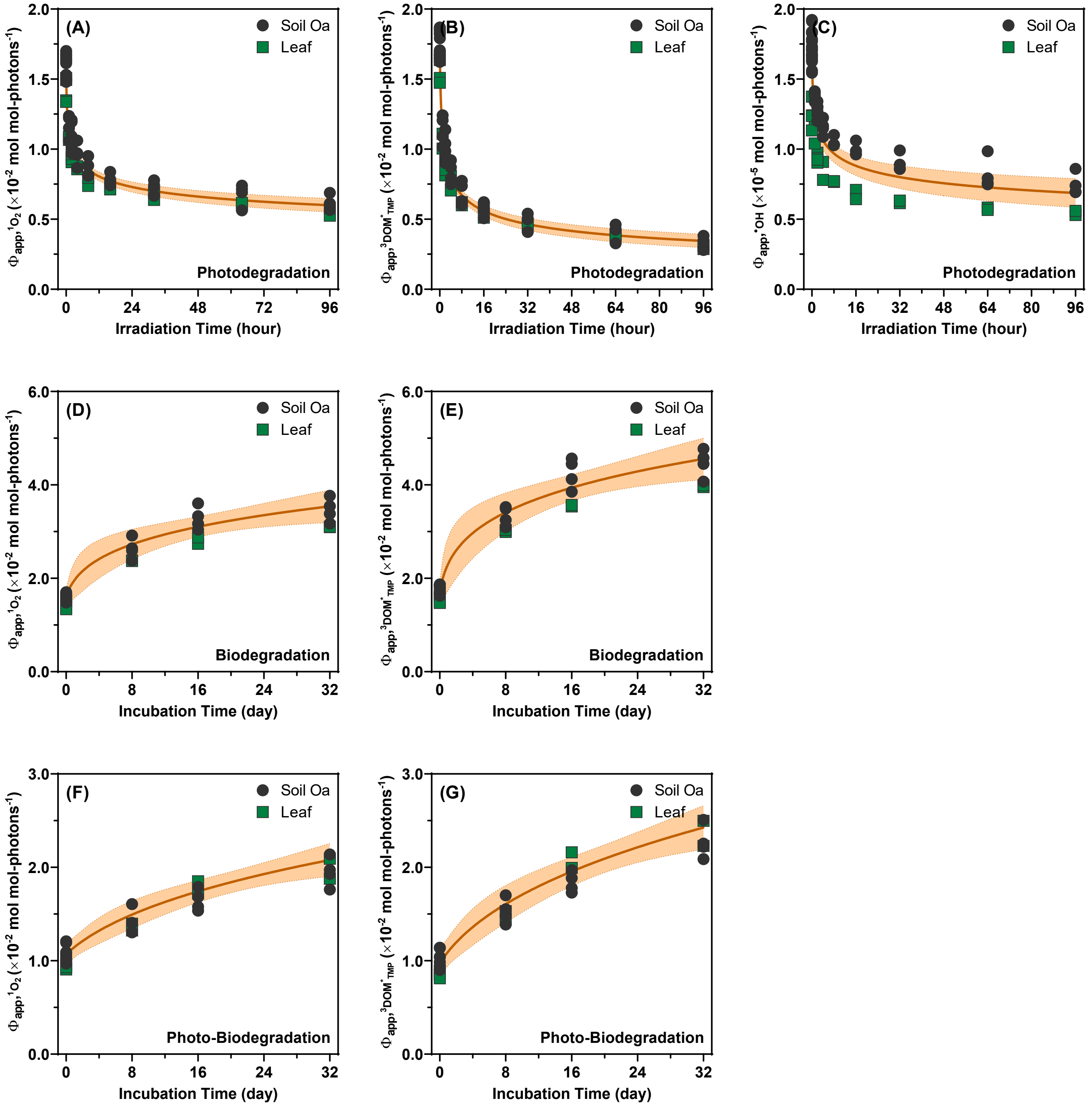

Figure S5. $\Phi_{app,RI}$ profile fittings by the gamma reactivity continuum model: **(A)** Model fitting for $\Phi_{app,\,^1O_2}$ for leaf and soil Oa leachates over 96 h of simulated sunlight irradiation. **(B)** Model fitting for $\Phi_{app,\,^3DOM^*_{TMP}}$ for leaf and soil Oa leachates over 96 h of irradiation. **(C)** Model fitting for $\Phi_{app,\,\cdot OH}$ for leaf and soil Oa leachates over 96 h of irradiation. **(D)** Model fitting for $\Phi_{app,\,^1O_2}$ for leaf and soil Oa leachates over 32 d of dark incubation. **(E)** Model fitting for $\Phi_{app,\,^3DOM^*_{TMP}}$ for leaf and soil Oa leachates over 32 d of incubation. **(F)** Model fitting for $\Phi_{app,\,^1O_2}$ for leaf and soil Oa leachates over 32 d of incubation with 2 h of prior irradiation. **(G)** Model fitting for $\Phi_{app,\,^3DOM^*_{TMP}}$ for leaf and soil Oa leachates over 32 d of incubation with 2 h of prior irradiation. For each plot, the solid line represents the nonlinear least squares regression line, and the shaded area brackets the 95% confidence interval of the regression line. $\Phi_{app,\,^1O_2}$ and $\Phi_{app,\,^3DOM^*_{TMP}}$ for leaf and soil Oa leachates from both tributary watersheds were pooled for the nonlinear least squares regression analysis.

11. Changes in $\Phi_{app,\ {}^1O_2}$, $\Phi_{app,\ {}^3DOM^*_{TMP}}$, and $\Phi_{app,\ \cdot OH}$ during photodegradation of SRFA and ESHA

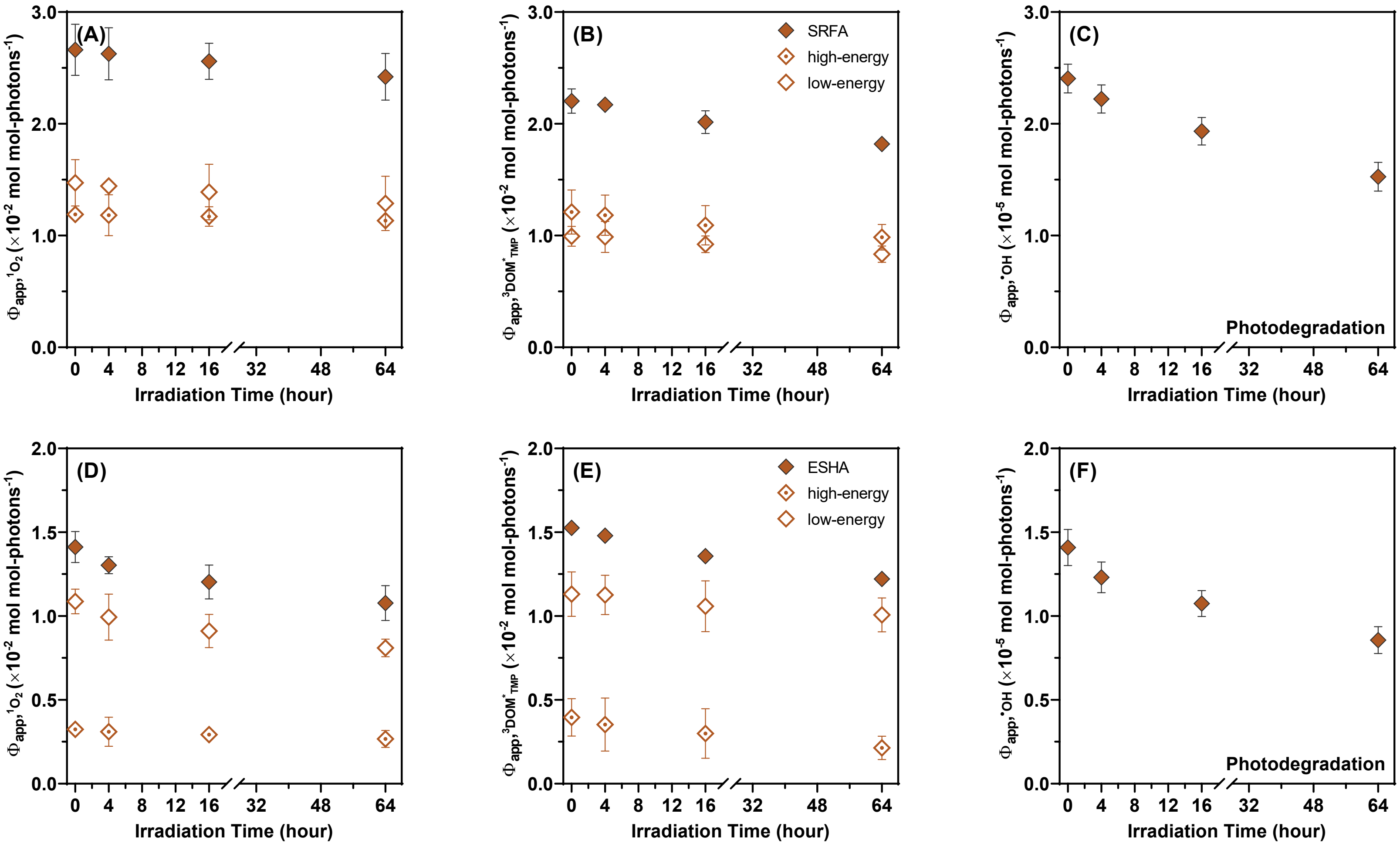

Figure S6. Changes in $\Phi_{app,\ {}^1O_2}$, $\Phi_{app,\ {}^3DOM^*_{TMP}}$, and $\Phi_{app,\ \cdot OH}$ during photodegradation of Suwannee River fulvic acid (SRFA; 3S101F) and Elliott Soil humic acid (ESHA; 5S102H): **(A)** Changes in $\Phi_{app,\ {}^1O_2}$ (i.e., the sum of $\Phi_{app,\ {}^1O_2,\ high\text{-}energy}$ and $\Phi_{app,\ {}^1O_2,\ low\text{-}energy}$) for SRFA over 64 h of simulated sunlight irradiation. **(B)** Changes in $\Phi_{app,\ {}^3DOM^*_{TMP}}$ (i.e., the sum of $\Phi_{app,\ {}^3DOM^*_{TMP},\ high\text{-}energy}$ and $\Phi_{app,\ {}^3DOM^*_{TMP},\ low\text{-}energy}$) for SRFA over 64 h of irradiation. **(C)** Changes in $\Phi_{app,\ \cdot OH}$ for SRFA over 64 h of irradiation. **(D)** Changes in $\Phi_{app,\ {}^1O_2}$ for ESHA over 64 h of irradiation. **(E)** Changes in $\Phi_{app,\ {}^3DOM^*_{TMP}}$ for ESHA over 64 h of irradiation. **(F)** Changes in $\Phi_{app,\ \cdot OH}$ for ESHA over 64 h of irradiation. $\Phi_{app,RI}$ for raw and photodegraded samples were measured under standardized irradiation and solution conditions ([DOC] = 4 mg C/L; pH 6.5±0.1). Error bars represent the standard deviations from duplicate or triplicate measurements; where absent, bars fall within symbols.

12. Changes in $\Phi_{app,\ ^1O_2}$, $\Phi_{app,\ ^3DOM^*_{TMP}}$, and $\Phi_{app,\ ^3DOM^*_{HDO}}$ during biodegradation of glucose and ESHA

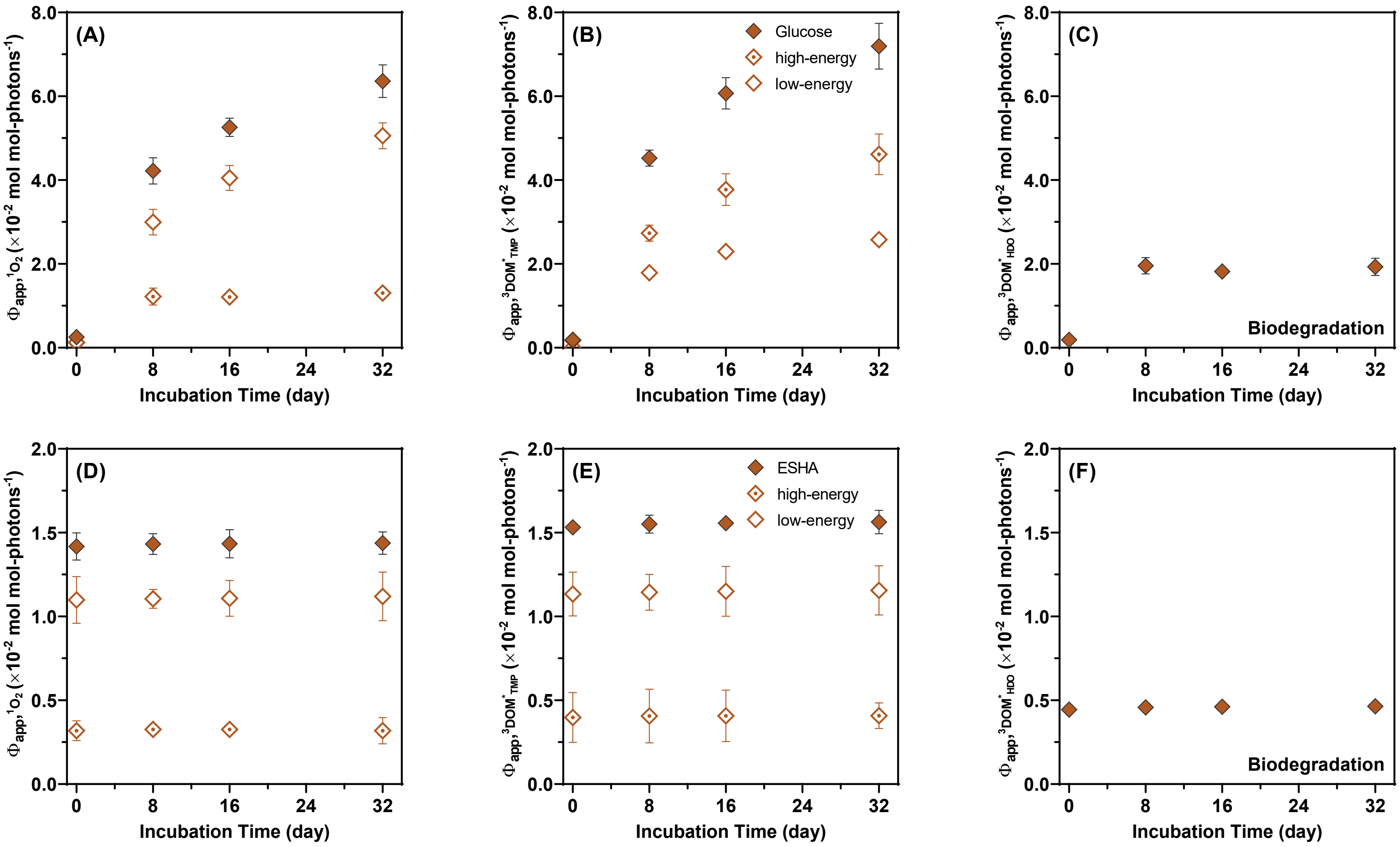

Figure S7. Changes in $\Phi_{app,\ ^1O_2}$, $\Phi_{app,\ ^3DOM^*_{TMP}}$, and $\Phi_{app,\ ^3DOM^*_{HDO}}$ during biodegradation of glucose and Elliott Soil humic acid (ESHA; 5S102H): **(A)** Changes in $\Phi_{app,\ ^1O_2}$ (i.e., the sum of $\Phi_{app,\ ^1O_2,\ high\text{-}energy}$ and $\Phi_{app,\ ^1O_2,\ low\text{-}energy}$) for glucose over 32 d of dark incubation. **(B)** Changes in $\Phi_{app,\ ^3DOM^*_{TMP}}$ (i.e., the sum of $\Phi_{app,\ ^3DOM^*_{TMP},\ high\text{-}energy}$ and $\Phi_{app,\ ^3DOM^*_{TMP},\ low\text{-}energy}$) for glucose over 32 d of incubation. **(C)** Changes in $\Phi_{app,\ ^3DOM^*_{HDO}}$ for glucose over glucose over 32 d of incubation. **(D)** Changes in $\Phi_{app,\ ^1O_2}$ for ESHA over 32 d of incubation. **(E)** Changes in $\Phi_{app,\ ^3DOM^*_{TMP}}$ for ESHA over 32 d of incubation. **(F)** Changes in $\Phi_{app,\ ^3DOM^*_{HDO}}$ for ESHA over 32 d of incubation. $\Phi_{app,RI}$ for raw and biodegraded samples were measured under standardized irradiation and solution conditions ([DOC] = 4 mg C/L; pH 6.5±0.1). Error bars represent the standard deviations from duplicate or triplicate measurements; where absent, bars fall within symbols.

13. Changes in $\Phi_{app, \cdot OH}$ during biodegradation and photo-biodegradation of leaf and soil Oa DOM

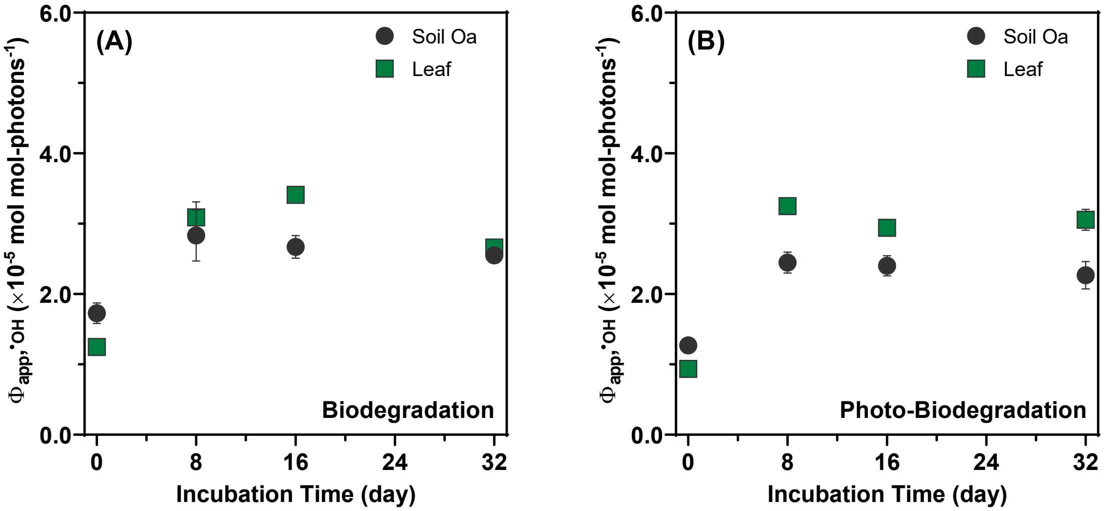

Figure S8. Changes in $\Phi_{app, \cdot OH}$ (after accounting for the contribution to $^{\bullet}OH$ production from NO_3^- photolysis) during biodegradation and photo-biodegradation of leaf and soil Oa leachate samples: **(A)** Changes in $\Phi_{app, \cdot OH}$ for leaf and soil Oa leachates over 32 d of dark incubation. **(B)** Changes in $\Phi_{app, \cdot OH}$ for leaf and soil Oa leachates over 32 d of incubation with 2 h of prior irradiation. $\Phi_{app, \cdot OH}$ for native and biodegraded/photo-biodegraded samples were measured under standardized irradiation and solution conditions ([DOC] = 4 mg C/L; pH 6.5±0.1). $\Phi_{app, \cdot OH}$ for soil Oa leachates from W16L (limed) and W24R (reference) tributary watersheds were pooled for the clarity of presentation as there was no significant difference between these two sample groups. Error bars represent the standard deviations from duplicate or triplicate measurements; where absent, bars fall within symbols.

14. Changes in the energy distribution of ^{3}DOM* contributing to $\Phi_{app,\,^1O_2}$

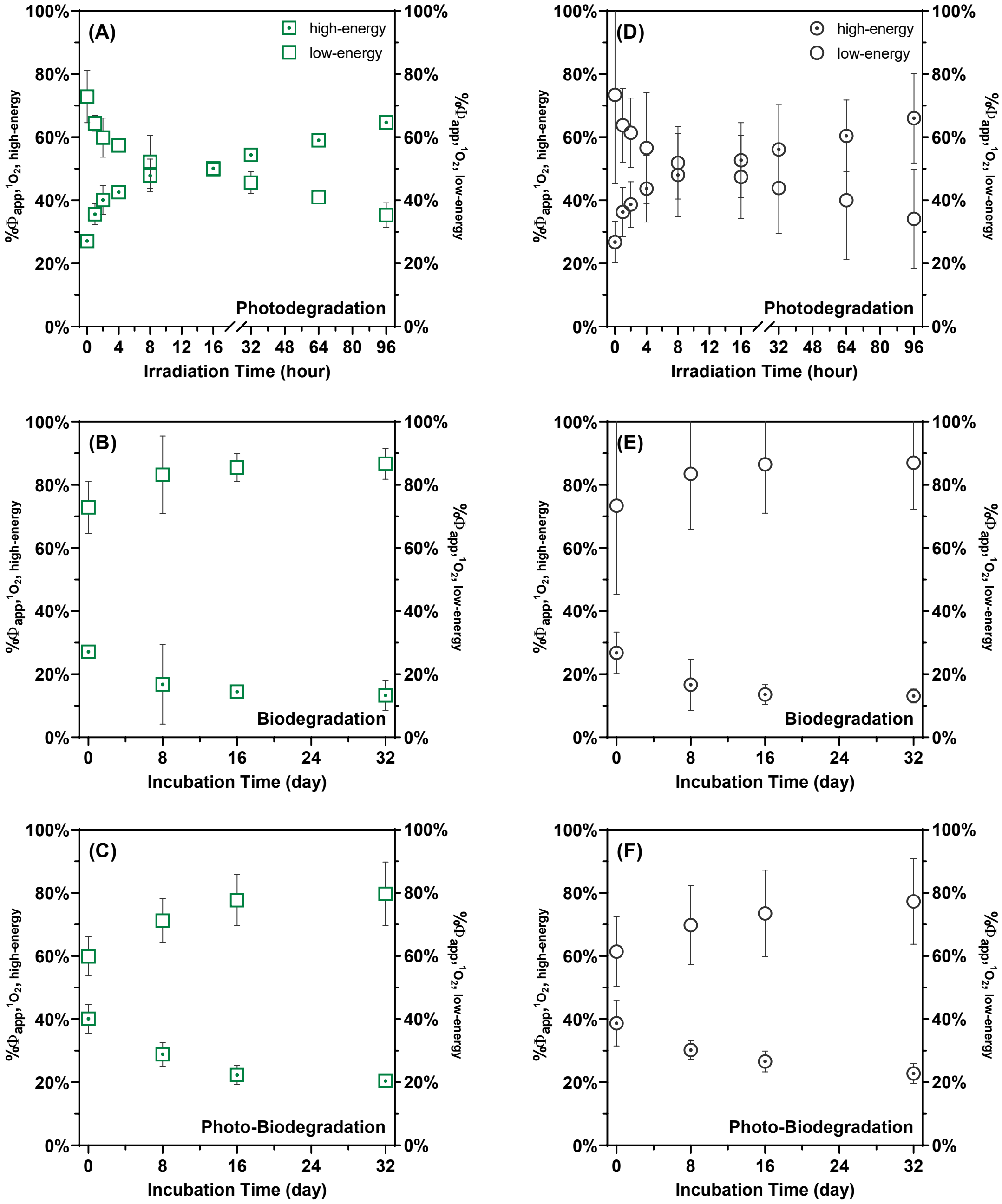

Figure S9. Changes in the percent contributions of high-energy ^{3}DOM* and low-energy ^{3}DOM* to $\Phi_{app,\,^1O_2}$ for photodegraded, biodegraded, or photo-biodegraded leaf and soil Oa leachates: **(A)** Changes in $\%\Phi_{app,\,^1O_2,\,high\text{-}energy}$ and $\%\Phi_{app,\,^1O_2,\,low\text{-}energy}$ for leaf leachates over 96 h of simulated sunlight irradiation. **(B)** Changes in $\%\Phi_{app,\,^1O_2,\,high\text{-}energy}$ and $\%\Phi_{app,\,^1O_2,\,low\text{-}energy}$ for leaf leachates over 32 d of dark incubation. **(C)** Changes in $\%\Phi_{app,\,^1O_2,\,high\text{-}energy}$ and $\%\Phi_{app,\,^1O_2,\,low\text{-}energy}$ for leaf leachates over 32 d of incubation with 2 h of prior irradiation. **(D)** Changes in $\%\Phi_{app,\,^1O_2,\,high\text{-}energy}$ and $\%\Phi_{app,\,^1O_2,\,low\text{-}energy}$ for soil Oa leachates over 96 h of irradiation. **(E)** Changes in $\%\Phi_{app,\,^1O_2,\,high\text{-}energy}$ and $\%\Phi_{app,\,^1O_2,\,low\text{-}energy}$ for soil Oa leachates over 32 d of incubation. **(F)** Changes in $\%\Phi_{app,\,^1O_2,\,high\text{-}energy}$ and $\%\Phi_{app,\,^1O_2,\,low\text{-}energy}$ for soil Oa leachates over 32 d of incubation with 2 h of prior irradiation. Error bars represent the standard deviations from duplicate or triplicate measurements; where absent, bars fall within symbols.

15. Changes in the energy distribution of $^{3}DOM^{*}$ contributing to $\Phi_{app,\ ^{3}DOM^{*}_{TMP}}$

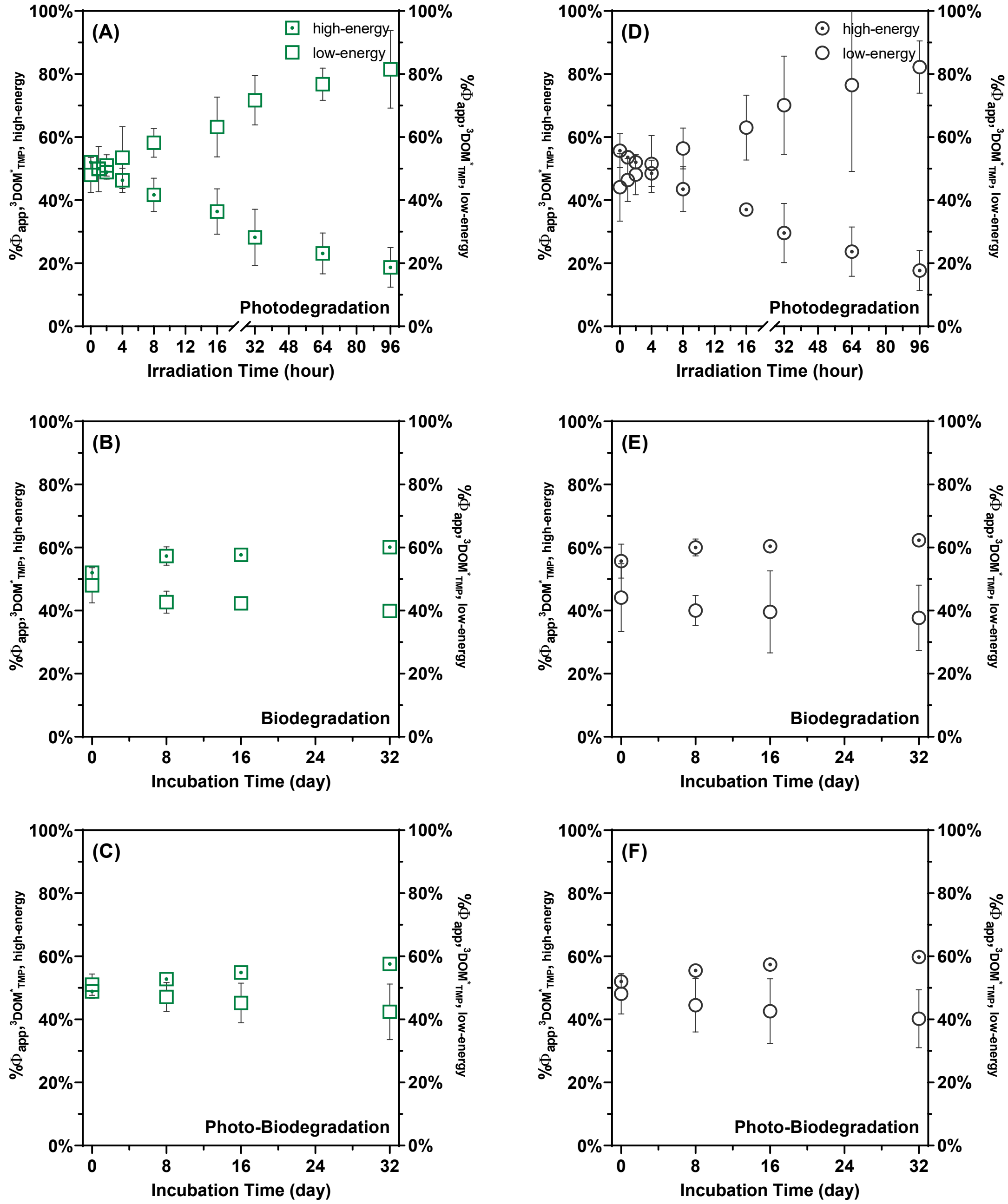

Figure S10. Changes in the percent contributions of high-energy $^{3}DOM^{*}$ and low-energy $^{3}DOM^{*}$ to $\Phi_{app,\ ^{3}DOM^{*}_{TMP}}$ for photodegraded, biodegraded, or photo-biodegraded leaf and soil Oa leachates: **(A)** Changes in $\%\Phi_{app,\ ^{3}DOM^{*}_{TMP},\ high\text{-}energy}$ and $\%\Phi_{app,\ ^{3}DOM^{*}_{TMP},\ low\text{-}energy}$ for leaf leachates over 96 h of simulated sunlight irradiation. **(B)** Changes in $\%\Phi_{app,\ ^{3}DOM^{*}_{TMP},\ high\text{-}energy}$ and $\%\Phi_{app,\ ^{3}DOM^{*}_{TMP},\ low\text{-}energy}$ for leaf leachates over 32 d of dark incubation. **(C)** Changes in $\%\Phi_{app,\ ^{3}DOM^{*}_{TMP},\ high\text{-}energy}$ and $\%\Phi_{app,\ ^{3}DOM^{*}_{TMP},\ low\text{-}energy}$ for leaf leachates over 32 d of incubation with 2 h of prior irradiation. **(D)** Changes in $\%\Phi_{app,\ ^{3}DOM^{*}_{TMP},\ high\text{-}energy}$ and $\%\Phi_{app,\ ^{3}DOM^{*}_{TMP},\ low\text{-}energy}$ for soil Oa leachates over 96 h of irradiation. **(E)** Changes in $\%\Phi_{app,\ ^{3}DOM^{*}_{TMP},\ high\text{-}energy}$ and $\%\Phi_{app,\ ^{3}DOM^{*}_{TMP},\ low\text{-}energy}$ for soil Oa leachates over 32 d of incubation. **(F)** Changes in $\%\Phi_{app,\ ^{3}DOM^{*}_{TMP},\ high\text{-}energy}$ and $\%\Phi_{app,\ ^{3}DOM^{*}_{TMP},\ low\text{-}energy}$ for soil Oa leachates over 32 d of incubation with 2 h of prior irradiation. Error bars represent the standard deviations from duplicate or triplicate measurements; where absent, bars fall within symbols.

16. Performance statistics of the multiple linear regression models

Table S17. Performance statistics of the multiple linear regression models for $\Phi_{app,RI}$

$\Phi_{app,\ ^1O_2}$ Model Criterion		Coefficient	Estimate	Standard Error	VIF	Normality of Residuals	*p* Value
Adjusted R^2	0.868	β0 (intercept)	-0.1937	0.01674	-	Anderson-Darling (A2*)	0.3047
RMSE	0.005	β1 ($S_{290–400}$ (μm^{-1}))	3.796×10^{-3}	6.771×10^{-4}	2.245	D'Agostino-Pearson omnibus (K2)	0.5303
AIC	-683.189	β2 (SUVA$_{254}$ (L mg C^{-1}•m^{-1}))	1.211×10^{-2}	1.004×10^{-3}	2.443	Shapiro-Wilk (W)	0.6566
SBC	-670.051	β3 (FI)	6.543×10^{-2}	6.005×10^{-3}	1.246	Kolmogorov-Smirnov (distance)	>0.1000
		β4 (*β:α*)	2.767×10^{-2}	1.362×10^{-2}	1.164		
		β5 (AOC (mmol e$^-$/g C))	-3.490×10^{-4}	1.491×10^{-4}	1.082		
$\Phi_{app,\ ^3DOM^*_{TMP}}$ Model Criterion		Coefficient	Estimate	Standard Error	VIF	Normality of Residuals	*p* Value
Adjusted R^2	0.848	β0 (intercept)	-0.2372	0.02218	-	Anderson-Darling (A2*)	0.6194
RMSE	0.007	β1 ($S_{290–400}$ (μm^{-1}))	4.748×10^{-3}	9.233×10^{-4}	2.241	D'Agostino-Pearson omnibus (K2)	0.6675
AIC	-643.967	β2 (SUVA$_{254}$ (L mg C^{-1}•m^{-1}))	1.447×10^{-2}	1.341×10^{-3}	2.338	Shapiro-Wilk (W)	0.8140
SBC	-635.209	β3 (FI)	8.525×10^{-2}	8.015×10^{-3}	1.191	Kolmogorov-Smirnov (distance)	>0.1000
$\Phi_{app,\ ^3DOM^*_{HDO}}$ Model Criterion		Coefficient	Estimate	Standard Error	VIF	Normality of Residuals	*p* Value
Adjusted R^2	0.856	β0 (intercept)	-0.04708	0.004643	-	Anderson-Darling (A2*)	0.1095
RMSE	0.002	β1 ($S_{290–400}$ (μm^{-1}))	1.021×10^{-3}	1.878×10^{-4}	2.245	D'Agostino-Pearson omnibus (K2)	0.0936
AIC	-852.467	β2 (SUVA$_{254}$ (L mg C^{-1}•m^{-1}))	3.262×10^{-3}	2.785×10^{-4}	2.443	Shapiro-Wilk (W)	0.1240
SBC	-839.329	β3 (FI)	1.473×10^{-2}	1.666×10^{-3}	1.246	Kolmogorov-Smirnov (distance)	>0.1000
		β4 (*β:α*)	1.522×10^{-2}	3.779×10^{-3}	1.164		
		β5 (AOC (mmol e$^-$/g C))	-3.403×10^{-4}	4.135×10^{-5}	1.082		

Adjusted R^2 = the adjusted determination coefficient for the model; RMSE = root mean square of the errors; AIC = Akaike information criterion; SBC = Bayesian information criterion; VIF = variance inflation factor.

17. Summary of literature data on the apparent quantum yields of RIs

Table S18. Summary of literature $\Phi_{app,RI}$ data

Source	Sample ID	Sample Classification	Wavelength Range (nm)	$\Phi_{app, ^3DOM^*_{TMP}}$ ($\times 10^{-2}$ mol mol-photons^{-1})	$\Phi_{app, ^1O_2}$ ($\times 10^{-2}$ mol mol-photons^{-1})	$\Phi_{app, ^3DOM^*_{Sorbate}}$ ($\times 10^{-2}$ mol mol-photons^{-1})	$\Phi_{app, ^{\bullet}OH}$ ($\times 10^{-6}$ mol mol-photons^{-1})
Ref 109	Ranker fulvic acid (FA)	XAD Fraction (Soil)	365	2.40	0.55	NA	NA
	Ranker humic acid (HA)	XAD Fraction (Soil)	365	1.30	0.36	NA	NA
	Commercial Aldrich humic acid (HA)	XAD Fraction (Soil)	365	0.69	0.20	NA	NA
	Rendzina humic acid (HA)	XAD Fraction (Soil)	365	0.36	0.11	NA	NA
	Podzol A 1 or Bb humic acid (HA)	XAD Fraction (Soil)	365	0.20	0.04	NA	NA
Ref 110	Aldrich humic acid (AHA)	XAD Fraction (Soil)	400 or 300-550	NA	0.10	NA	530.0
	Leonardite humic acid (LHA)	IHSS Isolate (LHA)	400 or 300-550	NA	0.40	NA	160.0
Ref 111	DOM-S1	RO/ED Isolate (Seawater)	280-500	2.36	1.24	NA	13.6
	DOM-S2	RO/ED Isolate (Seawater)	280-500	1.71	0.69	NA	10.3
	DOM-S3	RO/ED Isolate (Seawater)	280-500	1.75	0.82	NA	14.3
	SRNOM	IHSS Isolate (SRNOM)	280-500	1.25	0.76	NA	12.0
Ref 112	SDOM-1	RO/ED Isolate (Seawater)	290-400	0.86	0.92	0.72	45.9
	SDOM-2	RO/ED Isolate (Seawater)	290-400	1.29	0.75	0.54	36.3
	NRNOM	IHSS Isolate (NRNOM)	290-400	0.56	0.55	0.42	25.8
Ref 113	SRFA pH 7 ozone 0 mmol O_3 mmol C	IHSS Isolate (PLFA)	340-410	NA	1.00	NA	10.6
	SRFA pH 7 ozone 0.025 mmol O_3 mmol C	IHSS Isolate (PLFA, O_3)	340-410	NA	1.05	NA	10.6
	SRFA pH 7 ozone 0.055 mmol O_3 mmol C	IHSS Isolate (PLFA, O_3)	340-410	NA	1.15	NA	11.5
	SRFA pH 7 ozone 0.1 mmol O_3 mmol C	IHSS Isolate (PLFA, O_3)	340-410	NA	1.53	NA	14.2
	SRFA pH 7 ozone 0.15 mmol O_3 mmol C	IHSS Isolate (PLFA, O_3)	340-410	NA	1.69	NA	18.7
	SRFA pH 7 ozone 0.2 mmol O_3 mmol C	IHSS Isolate (PLFA, O_3)	340-410	NA	2.35	NA	43.4
	SRFA pH 7 ozone 0.25 mmol O_3 mmol C	IHSS Isolate (PLFA, O_3)	340-410	NA	3.02	NA	43.4
	SRFA pH 7 ozone 0.35 mmol O_3 mmol C	IHSS Isolate (PLFA, O_3)	340-410	NA	4.94	NA	59.4
	SRFA pH 7 ozone 0.5 mmol O_3 mmol C	IHSS Isolate (PLFA, O_3)	340-410	NA	7.47	NA	86.0
	PLFA pH 7 ozone 0 mmol O_3 mmol C	IHSS Isolate (SRFA)	340-410	NA	1.92	NA	14.9
	PLFA pH 7 ozone 0.025 mmol O_3 mmol C	IHSS Isolate (SRFA, O_3)	340-410	NA	2.67	NA	27.3
	PLFA pH 7 ozone 0.055 mmol O_3 mmol C	IHSS Isolate (SRFA, O_3)	340-410	NA	3.77	NA	30.8
	PLFA pH 7 ozone 0.1 mmol O_3 mmol C	IHSS Isolate (SRFA, O_3)	340-410	NA	4.31	NA	57.2
	PLFA pH 7 ozone 0.15 mmol O_3 mmol C	IHSS Isolate (SRFA, O_3)	340-410	NA	5.38	NA	82.7
	PLFA pH 7 ozone 0.2 mmol O_3 mmol C	IHSS Isolate (SRFA, O_3)	340-410	NA	6.51	NA	174.9
	PLFA pH 7 ozone 0.25 mmol O_3 mmol C	IHSS Isolate (SRFA, O_3)	340-410	NA	7.59	NA	188.2
	PLFA pH 7 ozone 0.35 mmol O_3 mmol C	IHSS Isolate (SRFA, O_3)	340-410	NA	9.48	NA	263.8
	PLFA pH 7 ozone 0.5 mmol O_3 mmol C	IHSS Isolate (SRFA, O_3)	340-410	NA	13.17	NA	428.9
Ref 114	DBC-W200	Dissolved Black Carbon (DBC)	NA	2.73	2.37	NA	NA
	DBC-W300	DBC	NA	3.22	2.66	NA	NA
	DBC-W400	DBC	NA	4.22	3.19	NA	NA
	DBC-W500	DBC	NA	2.18	1.19	NA	NA
	DBC-W600	DBC	NA	0.80	0.56	NA	NA
	DBC-R200	DBC	NA	0.83	0.87	NA	NA
	DBC-R300	DBC	NA	1.56	1.25	NA	NA

**Table S18.** Summary of literature $\Phi_{app,RI}$ data (continued)							
Source	Sample ID	Sample Classification	Wavelength Range (nm)	$\Phi_{app,\ ^3DOM^*_{TMP}}$ ($\times10^{-2}$ mol mol-photons^{-1})	$\Phi_{app,\ ^1O_2}$ ($\times10^{-2}$ mol mol-photons^{-1})	$\Phi_{app,\ ^3DOM^*_{Sorbate}}$ ($\times10^{-2}$ mol mol-photons^{-1})	$\Phi_{app,\ ^{\bullet}OH}$ ($\times10^{-6}$ mol mol-photons^{-1})
Ref 114	DBC-R400	DBC	NA	2.02	1.45	NA	NA
	DBC-R500	DBC	NA	0.74	0.80	NA	NA
	DBC-R600	DBC	NA	0.57	0.29	NA	NA
	DBC-B200	DBC	NA	1.45	1.17	NA	NA
	DBC-B300	DBC	NA	1.61	1.55	NA	NA
	DBC-B400	DBC	NA	3.43	2.31	NA	NA
	DBC-B500	DBC	NA	1.25	1.00	NA	NA
	DBC-B600	DBC	NA	0.83	0.85	NA	NA
	DBC-C200	DBC	NA	1.33	1.12	NA	NA
	DBC-C300	DBC	NA	2.60	2.23	NA	NA
	DBC-C400	DBC	NA	5.83	3.09	NA	NA
	DBC-C500	DBC	NA	0.88	0.37	NA	NA
	DBC-C600	DBC	NA	0.31	0.23	NA	NA
	DBC-P200	DBC	NA	1.40	0.79	NA	NA
	DBC-P300	DBC	NA	1.99	1.34	NA	NA
	DBC-P400	DBC	NA	2.08	1.47	NA	NA
	DBC-P500	DBC	NA	0.87	0.34	NA	NA
	DBC-P600	DBC	NA	0.57	0.29	NA	NA
Ref 115	Wet Season S1	Whole Water (WWTP-River)	290-400	1.23	2.39	NA	287.0
	Wet Season S2	Whole Water (WWTP-River)	290-400	1.81	4.11	NA	335.0
	Wet Season S3	Whole Water (WWTP-River)	290-400	1.56	3.85	NA	310.0
	Wet Season S4	Whole Water (WWTP-River)	290-400	2.19	4.56	NA	341.0
	Wet Season S5	Whole Water (WWTP-River)	290-400	1.54	4.00	NA	255.0
	Wet Season S6	Whole Water (WWTP-River)	290-400	1.41	3.56	NA	253.0
	Wet Season S7	Whole Water (WWTP-River)	290-400	1.33	3.31	NA	192.0
	Wet Season S8	Whole Water (WWTP-River)	290-400	1.23	2.14	NA	176.0
	Wet Season S9	Whole Water (WWTP-River)	290-400	1.36	3.53	NA	252.0
	Wet Season S10	Whole Water (WWTP-River)	290-400	0.97	1.38	NA	253.0
	Wet Season S11	Whole Water (WWTP-River)	290-400	0.75	0.95	NA	205.0
	Dry Season S1	Whole Water (WWTP-River)	290-400	1.35	3.15	NA	192.0
	Dry Season S2	Whole Water (WWTP-River)	290-400	1.25	2.71	NA	247.0
	Dry Season S3	Whole Water (WWTP-River)	290-400	1.25	3.09	NA	178.0
	Dry Season S4	Whole Water (WWTP-River)	290-400	1.04	1.89	NA	178.0
	Dry Season S5	Whole Water (WWTP-River)	290-400	0.90	1.17	NA	147.0
	Dry Season S6	Whole Water (WWTP-River)	290-400	0.97	1.49	NA	233.0
	Dry Season S7	Whole Water (WWTP-River)	290-400	0.81	1.05	NA	149.0
	Dry Season S8	Whole Water (WWTP-River)	290-400	0.55	0.94	NA	118.0
	Dry Season S9	Whole Water (WWTP-River)	290-400	1.46	3.32	NA	193.0
	Dry Season S10	Whole Water (WWTP-River)	290-400	0.90	1.70	NA	120.0
	Dry Season S11	Whole Water (WWTP-River)	290-400	0.53	0.88	NA	169.0
Ref 116	SRFA	IHSS Isolate (SRFA)	NA	NA	1.80	NA	32.8
	SRFA Fe/C 3.75	IHSS Isolate (SRFA, Ferrihydrite)	NA	NA	2.30	NA	45.8

**Table S18.** Summary of literature $\Phi_{app,RI}$ data (continued)							
Source	Sample ID	Sample Classification	Wavelength Range (nm)	$\Phi_{app,\ ^3DOM^*_{TMP}}$ (×10^{-2} mol mol-photons^{-1})	$\Phi_{app,\ ^1O_2}$ (×10^{-2} mol mol-photons^{-1})	$\Phi_{app,\ ^3DOM^*_{Sorbate}}$ (×10^{-2} mol mol-photons^{-1})	$\Phi_{app,\ ^{\bullet}OH}$ (×10^{-6} mol mol-photons^{-1})
Ref 116	SRFA Fe/C 11.25	IHSS Isolate (SRFA, Ferrihydrite)	NA	NA	3.28	NA	132.8
	NAFA	IHSS Isolate (NFA)	NA	NA	1.53	NA	31.0
	NAFA Fe/C 3.75	IHSS Isolate (NFA, Ferrihydrite)	NA	NA	1.72	NA	38.8
	NAFA Fe/C 11.25	IHSS Isolate (NFA, Ferrihydrite)	NA	NA	2.33	NA	108.2
	MNOM	IHSS Isolate (MRNOM)	NA	NA	2.28	NA	78.0
	MNOM Fe/C 3.75	IHSS Isolate (MRNOM, Ferrihydrite)	NA	NA	3.29	NA	149.2
	MNOM Fe/C 11.25	IHSS Isolate (MRNOM, Ferrihydrite)	NA	NA	6.16	NA	254.4
	SRNOM	IHSS Isolate (SRNOM)	NA	NA	1.81	NA	40.6
	SRNOM Fe/C 3.75	IHSS Isolate (SRNOM, Ferrihydrite)	NA	NA	3.32	NA	93.1
	SRNOM Fe/C 11.25	IHSS Isolate (SRNOM, Ferrihydrite)	NA	NA	3.64	NA	188.7
Ref 117	L-WEOM20	WEOM (Soil)	290-450	3.16	2.71	NA	61.0
	L-WEOM60	WEOM (Soil)	290-450	3.95	4.17	NA	69.0
	P-WEOM20	WEOM (Soil)	290-450	1.37	1.00	NA	15.0
	P-WEOM60	WEOM (Soil)	290-450	1.13	1.21	NA	25.0
	E-WEOM20	WEOM (Soil)	290-450	1.42	1.42	NA	12.0
	E-WEOM60	WEOM (Soil)	290-450	1.53	1.00	NA	30.0
	E-FA	IHSS Isolate (ESFA)	290-450	2.00	5.42	NA	NA
	E-HA	IHSS Isolate (ESHA)	290-450	1.47	2.08	NA	NA
Ref 118	Allier March	Whole Water (River)	300-450	NA	1.00	NA	NA
	Allier July	Whole Water (River)	300-450	NA	1.30	NA	NA
	Allier October	Whole Water (River)	300-450	NA	1.20	NA	NA
	B4 March	Whole Water (River)	300-450	NA	1.50	NA	NA
	B4 July	Whole Water (River)	300-450	NA	2.10	NA	NA
	B4 October	Whole Water (River)	300-450	NA	1.40	NA	NA
	B7 March	Whole Water (River)	300-450	NA	1.10	NA	NA
	B7 July	Whole Water (River)	300-450	NA	1.90	NA	NA
	B7 October	Whole Water (River)	300-450	NA	1.70	NA	NA
	Vendage March	Whole Water (River)	300-450	NA	0.78	NA	NA
	Vendage July	Whole Water (River)	300-450	NA	1.90	NA	NA
	Vendage October	Whole Water (River)	300-450	NA	1.20	NA	NA
	PZ1 March	Whole Water (Groundwater)	300-450	NA	3.00	NA	NA
	PZ1 July	Whole Water (Groundwater)	300-450	NA	3.10	NA	NA
	PZ1 October	Whole Water (Groundwater)	300-450	NA	4.00	NA	NA
	PZ5 March	Whole Water (Groundwater)	300-450	NA	5.00	NA	NA
	PZ5 July	Whole Water (Groundwater)	300-450	NA	2.90	NA	NA
	PZ5 October	Whole Water (Groundwater)	300-450	NA	6.30	NA	NA
Ref 119	CS (Henan Changsheng Industrial) pH 3	XAD Fraction (Soil)	532	NA	0.44	NA	NA
	AL (Aladdin Industrial) pH 3	XAD Fraction (Soil)	532	NA	0.06	NA	NA
	NA (Nordic Acid) pH 3	IHSS Isolate (NFA)	532	NA	0.19	NA	NA
	WP (Waskish Peat) pH 3	IHSS Isolate (WPFA)	532	NA	0.10	NA	NA
	CS (Henan Changsheng Industrial) pH 10	XAD Fraction (Soil)	532	NA	1.70	NA	NA
	AL (Aladdin Industrial) pH 10	XAD Fraction (Soil)	532	NA	0.29	NA	NA

**Table S18.** Summary of literature $\Phi_{app,RI}$ data (continued)							
Source	Sample ID	Sample Classification	Wavelength Range (nm)	$\Phi_{app,\ ^3DOM^*_{TMP}}$ ($\times10^{-2}$ mol mol-photons^{-1})	$\Phi_{app,\ ^1O_2}$ ($\times10^{-2}$ mol mol-photons^{-1})	$\Phi_{app,\ ^3DOM^*_{Sorbate}}$ ($\times10^{-2}$ mol mol-photons^{-1})	$\Phi_{app,\ ^{\bullet}OH}$ ($\times10^{-6}$ mol mol-photons^{-1})
Ref 119	NA (Nordic Acid) pH 10	IHSS Isolate (NFA)	532	NA	0.58	NA	NA
	WP (Waskish Peat) pH 10	IHSS Isolate (WPFA)	532	NA	0.36	NA	NA
Ref 120	SRNOM	IHSS Isolate (SRNOM)	290-400	2.07	0.93	NA	9.3
	CVT230	WEOM (Urban Biowaste-derived)	290-400	4.56	0.88	NA	4.3
	CVDF110	WEOM (Urban Biowaste-derived)	290-400	4.52	0.80	NA	4.1
Ref 121	EfOM1	C18 Extract (WWTP Effluent)	300-600	1.59	4.26	NA	45.6
	EfOM1 + UV 15 min	C18 Extract (WWTP Effluent, UV)	300-600	1.59	4.33	NA	50.1
	EfOM1 + UV 30 min	C18 Extract (WWTP Effluent, UV)	300-600	1.59	4.39	NA	58.9
	EfOM1 + UV 60 min	C18 Extract (WWTP Effluent, UV)	300-600	1.63	4.40	NA	68.8
	EfOM1 + UV 120 min	C18 Extract (WWTP Effluent, UV)	300-600	1.71	4.41	NA	92.3
	EfOM1 + UV 240 min	C18 Extract (WWTP Effluent, UV)	300-600	1.76	4.40	NA	125.9
	EfOM2	C18 Extract (WWTP Effluent)	300-600	1.51	4.12	NA	43.0
	EfOM2 + UV 15 min	C18 Extract (WWTP Effluent, UV)	300-600	1.53	4.27	NA	50.7
	EfOM2 + UV 30 min	C18 Extract (WWTP Effluent, UV)	300-600	1.55	4.33	NA	64.0
	EfOM2 + UV 60 min	C18 Extract (WWTP Effluent, UV)	300-600	1.61	4.36	NA	78.7
	EfOM2 + UV 120 min	C18 Extract (WWTP Effluent, UV)	300-600	1.80	4.45	NA	102.4
	EfOM2 + UV 240 min	C18 Extract (WWTP Effluent, UV)	300-600	1.82	4.67	NA	148.1
	EfOM3	C18 Extract (WWTP Effluent)	300-600	1.97	5.08	NA	55.7
	EfOM3 + UV 15 min	C18 Extract (WWTP Effluent, UV)	300-600	1.97	5.18	NA	59.3
	EfOM3 + UV 30 min	C18 Extract (WWTP Effluent, UV)	300-600	1.98	5.20	NA	68.6
	EfOM3 + UV 60 min	C18 Extract (WWTP Effluent, UV)	300-600	2.03	5.20	NA	81.8
	EfOM3 + UV 120 min	C18 Extract (WWTP Effluent, UV)	300-600	2.21	5.21	NA	103.6
	EfOM3 + UV 240 min	C18 Extract (WWTP Effluent, UV)	300-600	2.31	5.22	NA	134.4
	Henan Changsheng Industrial fulvic acid (FA)	XAD Fraction (Soil)	300-600	1.29	3.10	NA	3.0
	FA + UV 15 min	XAD Fraction (Soil, UV)	300-600	1.23	2.95	NA	3.4
	FA + UV 30 min	XAD Fraction (Soil, UV)	300-600	1.23	2.73	NA	3.7
	FA + UV 60 min	XAD Fraction (Soil, UV)	300-600	1.04	2.43	NA	4.7
	FA + UV 120 min	XAD Fraction (Soil, UV)	300-600	0.82	1.96	NA	5.1
	FA + UV 240 min	XAD Fraction (Soil, UV)	300-600	0.54	1.37	NA	5.2
	Aldrich humic acid (HA)	XAD Fraction (Soil)	300-600	1.02	2.47	NA	10.7
	HA + UV 15 min	XAD Fraction (Soil, UV)	300-600	0.97	2.34	NA	11.1
	HA + UV 30 min	XAD Fraction (Soil, UV)	300-600	0.93	2.30	NA	12.5
	HA + UV 60 min	XAD Fraction (Soil, UV)	300-600	0.82	2.10	NA	14.2
	HA + UV 120 min	XAD Fraction (Soil, UV)	300-600	0.68	1.82	NA	16.2
	HA + UV 240 min	XAD Fraction (Soil, UV)	300-600	0.54	1.41	NA	18.0
Ref 122	rice	DBC	300-400	4.12	3.75	NA	60.8
	corn	DBC	300-400	4.68	3.96	NA	9.9
	wheat	DBC	300-400	5.62	4.25	NA	14.3
Ref 123	EfOM	C18 Extract (WWTP Effluent)	300-400	4.10	3.85	NA	77.3
	EfOM + 1.0 mg/L Cl_2	C18 Extract (WWTP Effluent, Cl_2)	300-400	4.60	4.28	NA	90.9
	EfOM + 2.0 mg/L Cl_2	C18 Extract (WWTP Effluent, Cl_2)	300-400	5.29	4.96	NA	112.3
	EfOM + 5.0 mg/L Cl_2	C18 Extract (WWTP Effluent, Cl_2)	300-400	6.44	6.23	NA	132.3

Table S18. Summary of literature $\Phi_{app,RI}$ data (continued)

Source	Sample ID	Sample Classification	Wavelength Range (nm)	$\Phi_{app,\ ^3DOM^*_{TMP}}$ ($\times 10^{-2}$ mol mol-photons^{-1})	$\Phi_{app,\ ^1O_2}$ ($\times 10^{-2}$ mol mol-photons^{-1})	$\Phi_{app,\ ^3DOM^*_{Sorbate}}$ ($\times 10^{-2}$ mol mol-photons^{-1})	$\Phi_{app,\ ^{\bullet}OH}$ ($\times 10^{-6}$ mol mol-photons^{-1})
Ref 123	EfOM + 10.0 mg/L Cl_2	C18 Extract (WWTP Effluent, Cl_2)	300-400	8.30	8.36	NA	175.1
	Henan Changsheng Industrial fulvic acid (FA)	XAD Fraction (Soil)	300-400	2.27	2.31	NA	3.6
	FA + 1.0 mg/L Cl_2	XAD Fraction (Soil, Cl_2)	300-400	2.56	2.58	NA	6.6
	FA + 2.0 mg/L Cl_2	XAD Fraction (Soil, Cl_2)	300-400	3.09	3.08	NA	12.0
	FA + 5.0 mg/L Cl_2	XAD Fraction (Soil, Cl_2)	300-400	3.61	3.78	NA	22.7
	FA + 10.0 mg/L Cl_2	XAD Fraction (Soil, Cl_2)	300-400	4.61	4.94	NA	31.5
	Aldrich humic acid (HA)	XAD Fraction (Soil)	300-400	2.81	2.24	NA	9.7
	HA + 1.0 mg/L Cl_2	XAD Fraction (Soil, Cl_2)	300-400	2.94	2.38	NA	10.8
	HA + 2.0 mg/L Cl_2	XAD Fraction (Soil, Cl_2)	300-400	3.18	2.60	NA	12.0
	HA + 5.0 mg/L Cl_2	XAD Fraction (Soil, Cl_2)	300-400	4.07	3.49	NA	15.6
	HA + 10.0 mg/L Cl_2	XAD Fraction (Soil, Cl_2)	300-400	5.05	4.38	NA	23.3
Ref 124	EfOM-1	C18 Extract (WWTP Effluent)	290-400	2.44	6.33	NA	48.0
	EfOM-1 + $KMnO_4$ 0.5 mg L^{-1}	C18 Extract (WWTP Effluent, $KMnO_4$)	290-400	2.69	6.97	NA	48.1
	EfOM-1 + $KMnO_4$ 1.0 mg L^{-1}	C18 Extract (WWTP Effluent, $KMnO_4$)	290-400	2.76	7.06	NA	43.7
	EfOM-1 + $KMnO_4$ 2.0 mg L^{-1}	C18 Extract (WWTP Effluent, $KMnO_4$)	290-400	2.74	7.32	NA	42.9
	EfOM-1 + $KMnO_4$ 4.0 mg L^{-1}	C18 Extract (WWTP Effluent, $KMnO_4$)	290-400	2.75	7.22	NA	41.7
	EfOM-2	C18 Extract (WWTP Effluent)	290-400	1.31	4.52	NA	43.9
	EfOM-2 + $KMnO_4$ 0.5 mg L^{-1}	C18 Extract (WWTP Effluent, $KMnO_4$)	290-400	1.40	4.73	NA	42.7
	EfOM-2 + $KMnO_4$ 1.0 mg L^{-1}	C18 Extract (WWTP Effluent, $KMnO_4$)	290-400	1.42	4.88	NA	40.6
	EfOM-2 + $KMnO_4$ 2.0 mg L^{-1}	C18 Extract (WWTP Effluent, $KMnO_4$)	290-400	1.41	4.91	NA	39.6
	EfOM-2 + $KMnO_4$ 4.0 mg L^{-1}	C18 Extract (WWTP Effluent, $KMnO_4$)	290-400	1.43	5.00	NA	39.4
	Henan Changsheng Industrial Fulvic acid (FA)	XAD Fraction (Soil)	290-400	0.73	2.59	NA	2.3
	FA + $KMnO_4$ 0.5 mg L^{-1}	XAD Fraction (Soil, $KMnO_4$)	290-400	0.77	2.64	NA	2.2
	FA + $KMnO_4$ 1.0 mg L^{-1}	XAD Fraction (Soil, $KMnO_4$)	290-400	0.80	2.73	NA	2.0
	FA + $KMnO_4$ 2.0 mg L^{-1}	XAD Fraction (Soil, $KMnO_4$)	290-400	0.83	2.90	NA	2.0
	FA + $KMnO_4$ 4.0 mg L^{-1}	XAD Fraction (Soil, $KMnO_4$)	290-400	0.83	2.95	NA	1.8
	Aldrich humic acid (HA)	XAD Fraction (Soil)	290-400	0.81	2.22	NA	9.0
	HA + $KMnO_4$ 0.5 mg L^{-1}	XAD Fraction (Soil, $KMnO_4$)	290-400	0.87	2.39	NA	8.5
	HA + $KMnO_4$ 1.0 mg L^{-1}	XAD Fraction (Soil, $KMnO_4$)	290-400	0.89	2.44	NA	8.0
	HA + $KMnO_4$ 2.0 mg L^{-1}	XAD Fraction (Soil, $KMnO_4$)	290-400	0.90	2.44	NA	7.5
	HA + $KMnO_4$ 4.0 mg L^{-1}	XAD Fraction (Soil, $KMnO_4$)	290-400	0.94	2.42	NA	6.8
	NOM-1	Whole Water (Lake)	290-400	1.83	6.43	NA	47.0
	NOM-1 + $KMnO_4$ 0.5 mg L^{-1}	Whole Water (Lake, $KMnO_4$)	290-400	1.97	6.62	NA	42.9
	NOM-1 + $KMnO_4$ 1.0 mg L^{-1}	Whole Water (Lake, $KMnO_4$)	290-400	1.99	6.54	NA	41.7
	NOM-1 + $KMnO_4$ 2.0 mg L^{-1}	Whole Water (Lake, $KMnO_4$)	290-400	2.16	6.70	NA	40.9
	NOM-1 + $KMnO_4$ 4.0 mg L^{-1}	Whole Water (Lake, $KMnO_4$)	290-400	2.28	6.71	NA	41.0
	NOM-2	Whole Water (Lake)	290-400	1.53	5.13	NA	87.0
	NOM-2 + $KMnO_4$ 0.5 mg L^{-1}	Whole Water (Lake, $KMnO_4$)	290-400	1.66	5.28	NA	79.7
	NOM-2 + $KMnO_4$ 1.0 mg L^{-1}	Whole Water (Lake, $KMnO_4$)	290-400	1.80	5.30	NA	79.6
	NOM-2 + $KMnO_4$ 2.0 mg L^{-1}	Whole Water (Lake, $KMnO_4$)	290-400	2.02	5.30	NA	77.8
	NOM-2 + $KMnO_4$ 4.0 mg L^{-1}	Whole Water (Lake, $KMnO_4$)	290-400	2.13	5.31	NA	70.7
	NOM-3	Whole Water (Lake)	290-400	1.37	3.90	NA	32.2

Table S18. Summary of literature $\Phi_{app,RI}$ data (continued)							
Source	Sample ID	Sample Classification	Wavelength Range (nm)	$\Phi_{app,\ ^3DOM^*_{TMP}}$ ($\times10^{-2}$ mol mol-photons^{-1})	$\Phi_{app,\ ^1O_2}$ ($\times10^{-2}$ mol mol-photons^{-1})	$\Phi_{app,\ ^3DOM^*_{Sorbate}}$ ($\times10^{-2}$ mol mol-photons^{-1})	$\Phi_{app,\ ^{\bullet}OH}$ ($\times10^{-6}$ mol mol-photons^{-1})
Ref 124	NOM-3 + $KMnO_4$ 0.5 mg L^{-1}	Whole Water (Lake, $KMnO_4$)	290-400	1.56	4.13	NA	27.9
	NOM-3 + $KMnO_4$ 1.0 mg L^{-1}	Whole Water (Lake, $KMnO_4$)	290-400	1.58	4.30	NA	27.0
	NOM-3 + $KMnO_4$ 2.0 mg L^{-1}	Whole Water (Lake, $KMnO_4$)	290-400	1.68	4.27	NA	27.4
	NOM-3 + $KMnO_4$ 4.0 mg L^{-1}	Whole Water (Lake, $KMnO_4$)	290-400	1.79	4.54	NA	27.4
	NOM-4	Whole Water (Lake)	290-400	2.63	7.67	NA	44.1
	NOM-4 + $KMnO_4$ 0.5 mg L^{-1}	Whole Water (Lake, $KMnO_4$)	290-400	2.89	7.96	NA	42.2
	NOM-4 + $KMnO_4$ 1.0 mg L^{-1}	Whole Water (Lake, $KMnO_4$)	290-400	2.94	7.89	NA	42.0
	NOM-4 + $KMnO_4$ 2.0 mg L^{-1}	Whole Water (Lake, $KMnO_4$)	290-400	2.94	8.07	NA	40.0
	NOM-4 + $KMnO_4$ 4.0 mg L^{-1}	Whole Water (Lake, $KMnO_4$)	290-400	3.07	8.28	NA	40.3
Ref 125	DOM-4#	RO/ED Isolate (Seawater)	290-400	8.68	NA	NA	NA
	DOM-6#	RO/ED Isolate (Seawater)	290-400	5.47	NA	NA	NA
	DOM-8#	RO/ED Isolate (Seawater)	290-400	4.73	NA	NA	NA
	DOM-9#	RO/ED Isolate (Seawater)	290-400	5.71	NA	NA	NA
Ref 126	CS-DOM-1#	RO/ED Isolate (Seawater)	290-400	1.34	3.36	NA	19.3
	CS-DOM-2#	RO/ED Isolate (Seawater)	290-400	1.36	2.75	NA	10.5
	SRFA	IHSS Isolate (SRFA)	290-400	0.64	2.20	NA	18.4
	SRNOM	IHSS Isolate (SRNOM)	290-400	0.86	2.79	NA	22.0
Ref 46	SRNOM	IHSS Isolate (SRNOM)	375	2.48	0.84	NA	6.5
	SRNOM	IHSS Isolate (SRNOM)	387	1.37	0.55	NA	3.7
	SRNOM	IHSS Isolate (SRNOM)	425	1.27	0.55	NA	3.0
	SRNOM	IHSS Isolate (SRNOM)	461	1.06	0.35	NA	1.8
	SRNOM	IHSS Isolate (SRNOM)	490	1.07	0.16	NA	1.2
Ref 127	Irrigation	Whole Water (Paddy)	290-400	1.76	11.01	NA	17.8
	Seedling1	Whole Water (Paddy)	290-400	2.15	12.72	NA	74.9
	Seedling2	Whole Water (Paddy)	290-400	1.70	7.65	NA	10.8
	Seedling3	Whole Water (Paddy)	290-400	1.65	9.29	NA	11.9
	Seedling4	Whole Water (Paddy)	290-400	2.24	15.59	NA	13.6
	Tillering1	Whole Water (Paddy)	290-400	1.22	3.95	NA	12.1
	Tillering2	Whole Water (Paddy)	290-400	1.42	5.00	NA	24.8
	Tillering3	Whole Water (Paddy)	290-400	1.17	3.14	NA	12.2
	Tillering4	Whole Water (Paddy)	290-400	1.25	2.00	NA	12.2
	Jointing1	Whole Water (Paddy)	290-400	2.91	8.99	NA	54.2
	Jointing2	Whole Water (Paddy)	290-400	3.40	13.39	NA	57.8
	Heading1	Whole Water (Paddy)	290-400	1.13	4.25	NA	10.4
	Heading2	Whole Water (Paddy)	290-400	1.69	3.92	NA	10.4
	Irrigation	Whole Water (Paddy)	290-400	1.76	11.01	NA	17.8
Ref 128	SRFA	IHSS Isolate (SRFA)	290-500	NA	1.61	NA	NA
Ref 129	SRFA	IHSS Isolate (SRFA)	290-400	1.29	1.23	NA	22.1
	SRHA	IHSS Isolate (SRHA)	290-400	0.54	0.74	NA	14.1
	SRNOM	IHSS Isolate (SRNOM)	290-400	1.71	1.44	NA	25.5

**Table S18.** Summary of literature $\Phi_{app,RI}$ data (continued)							
Source	Sample ID	Sample Classification	Wavelength Range (nm)	$\Phi_{app,\ ^3DOM^*_{TMP}}$ ($\times10^{-2}$ mol mol-photons^{-1})	$\Phi_{app,\ ^1O_2}$ ($\times10^{-2}$ mol mol-photons^{-1})	$\Phi_{app,\ ^3DOM^*_{Sorbate}}$ ($\times10^{-2}$ mol mol-photons^{-1})	$\Phi_{app,\ ^{\cdot}OH}$ ($\times10^{-6}$ mol mol-photons^{-1})
Ref 129	PLFA	IHSS Isolate (PLFA)	290-400	4.26	2.90	NA	52.0
	NAFA	IHSS Isolate (NFA)	290-400	0.94	0.92	NA	19.6
	MRNOM	IHSS Isolate (MRNOM)	290-400	2.49	2.11	NA	26.3
	SDOM	RO/ED Isolate (Seawater)	290-400	3.34	3.31	NA	86.2
Ref 130	Fresh water (L1)	Whole Water (River)	280-400	8.25	7.67	NA	79.0
	Estuarine water (L2)	Whole Water (Estuary)	280-400	7.25	6.70	NA	324.0
	Seawater (L3)	Whole Water (Ocean)	280-400	3.07	1.94	NA	68.7
	SRFA	IHSS Isolate (SRFA)	280-400	2.09	2.83	NA	48.1
	SRHA	IHSS Isolate (SRHA)	280-400	1.35	1.83	NA	32.2
	PLFA	IHSS Isolate (PLFA)	280-400	5.60	4.96	NA	107.0
Ref 131	SRFA initial	IHSS Isolate (SRFA)	290-400	4.66	1.25	NA	56.0
	SRFA UV/Cl_2 0 µM	IHSS Isolate (SRFA, UV/Cl_2)	290-400	3.86	0.81	NA	49.0
	SRFA UV/Cl_2 25 µM	IHSS Isolate (SRFA, UV/Cl_2)	290-400	4.45	1.15	NA	51.8
	SRFA UV/Cl_2 50 µM	IHSS Isolate (SRFA, UV/Cl_2)	290-400	4.66	1.24	NA	58.0
	SRFA UV/Cl_2 75 µM	IHSS Isolate (SRFA, UV/Cl_2)	290-400	4.86	1.36	NA	59.6
	SRFA UV/Cl_2 100 µM	IHSS Isolate (SRFA, UV/Cl_2)	290-400	4.96	1.32	NA	60.6
	SRFA UV/Cl_2 125 µM	IHSS Isolate (SRFA, UV/Cl_2)	290-400	5.16	1.44	NA	61.6
	SRFA UV/Cl_2 150 µM	IHSS Isolate (SRFA, UV/Cl_2)	290-400	5.25	1.50	NA	64.9
	SRFA UV/Cl_2 200 µM	IHSS Isolate (SRFA, UV/Cl_2)	290-400	5.56	1.70	NA	71.1
	SRNOM initial	IHSS Isolate (SRNOM)	290-400	4.07	1.00	NA	42.6
	SRNOM UV/Cl_2 0 µM	IHSS Isolate (SRNOM, UV/Cl_2)	290-400	3.79	0.40	NA	41.7
	SRNOM UV/Cl_2 25 µM	IHSS Isolate (SRNOM, UV/Cl_2)	290-400	3.55	0.82	NA	46.9
	SRNOM UV/Cl_2 50 µM	IHSS Isolate (SRNOM, UV/Cl_2)	290-400	3.91	0.96	NA	48.2
	SRNOM UV/Cl_2 75 µM	IHSS Isolate (SRNOM, UV/Cl_2)	290-400	3.90	1.13	NA	48.7
	SRNOM UV/Cl_2 100 µM	IHSS Isolate (SRNOM, UV/Cl_2)	290-400	4.40	1.19	NA	49.5
	SRNOM UV/Cl_2 125 µM	IHSS Isolate (SRNOM, UV/Cl_2)	290-400	4.49	1.42	NA	53.3
	SRNOM UV/Cl_2 150 µM	IHSS Isolate (SRNOM, UV/Cl_2)	290-400	5.89	1.78	NA	70.6
	SRNOM UV/Cl_2 200 µM	IHSS Isolate (SRNOM, UV/Cl_2)	290-400	7.00	2.33	NA	80.6
Ref 132	M-EPS (*Shewanella oneidensis* MR-1)	Extracellular Polymeric Substances	280-700	NA	4.01	NA	4.3
	E-EPS (*Escherichia coli*)	Extracellular Polymeric Substances	280-700	NA	4.08	NA	22.0
	S-EPS (Mixed culture sewage sludge flocs)	Extracellular Polymeric Substances	280-700	NA	7.40	NA	19.4
This work	W16L Soil Oa Photo 0 h	WEOM (Soil, photo)	290-550	1.73±0.13	1.56±0.10	0.59±0.01	17.47±0.45
	W16L Soil Oa Photo 1 h	WEOM (Soil, photo)	290-550	1.05±0.05	1.11±0.06	0.58±0.01	14.06±0.10
	W16L Soil Oa Photo 2 h	WEOM (Soil, photo)	290-550	0.94±0.06	1.03±0.09	0.58±0.02	13.22±0.26
	W16L Soil Oa Photo 4 h	WEOM (Soil, photo)	290-550	0.77±0.03	0.92±0.07	0.58±0.03	11.87±0.53
	W16L Soil Oa Photo 8 h	WEOM (Soil, photo)	290-550	0.62±0.02	0.81±0.01	0.57±0.03	10.67±0.49
	W16L Soil Oa Photo 16 h	WEOM (Soil, photo)	290-550	0.53±0.04	0.75±0.02	0.58±0.09	10.12±0.70
	W16L Soil Oa Photo 32 h	WEOM (Soil, photo)	290-550	0.45±0.06	0.70±0.04	0.57±0.08	9.25±0.94
	W16L Soil Oa Photo 64 h	WEOM (Soil, photo)	290-550	0.38±0.07	0.65±0.12	0.57±0.09	8.67±1.67
	W16L Soil Oa Photo 96 h	WEOM (Soil, photo)	290-550	0.29±0.01	0.58±0.03	0.57±0.10	7.99±0.85
	W24R Soil Oa Photo 0 h	WEOM (Soil, photo)	290-550	1.74±0.08	1.56±0.08	0.59±0.04	16.71±0.08

Table S18. Summary of literature $\Phi_{app,RI}$ data (continued)							
Source	Sample ID	Sample Classification	Wavelength Range (nm)	$\Phi_{app, ^3DOM^*_{TMP}}$ (×10^{-2} mol mol-photons^{-1})	$\Phi_{app, ^1O_2}$ (×10^{-2} mol mol-photons^{-1})	$\Phi_{app, ^3DOM^*_{Sorbate}}$ (×10^{-2} mol mol-photons^{-1})	$\Phi_{app, ^{\bullet}OH}$ (×10^{-6} mol mol-photons^{-1})
This work	W24R Soil Oa Photo 1 h	WEOM (Soil, photo)	290-550	1.22±0.03	1.23±0.01	0.60±0.05	13.60±0.31
	W24R Soil Oa Photo 2 h	WEOM (Soil, photo)	290-550	1.09±0.07	1.14±0.10	0.60±0.01	12.51±0.65
	W24R Soil Oa Photo 4 h	WEOM (Soil, photo)	290-550	0.90±0.04	1.01±0.07	0.59±0.01	11.27±0.55
	W24R Soil Oa Photo 8 h	WEOM (Soil, photo)	290-550	0.76±0.03	0.92±0.05	0.59±0.02	10.30±0.03
	W24R Soil Oa Photo 16 h	WEOM (Soil, photo)	290-550	0.60±0.03	0.81±0.04	0.59±0.04	9.77±0.15
	W24R Soil Oa Photo 32 h	WEOM (Soil, photo)	290-550	0.52±0.02	0.76±0.02	0.59±0.02	8.75±0.20
	W24R Soil Oa Photo 64 h	WEOM (Soil, photo)	290-550	0.44±0.02	0.70±0.02	0.58±0.04	7.76±0.23
	W24R Soil Oa Photo 96 h	WEOM (Soil, photo)	290-550	0.37±0.02	0.65±0.05	0.59±0.01	6.94±0.02
	Leaf Photo 0 h	WEOM (Leaf, photo)	290-550	1.57±0.13	1.42±0.12	0.54±0.03	12.54±1.70
	Leaf Photo 1 h	WEOM (Leaf, photo)	290-550	1.06±0.07	1.08±0.02	0.53±0.01	11.10±0.99
	Leaf Photo 2 h	WEOM (Leaf, photo)	290-550	0.85±0.05	0.93±0.01	0.53±0.07	9.39±0.51
	Leaf Photo 4 h	WEOM (Leaf, photo)	290-550	0.76±0.07	0.87±0.01	0.53±0.06	8.45±0.91
	Leaf Photo 8 h	WEOM (Leaf, photo)	290-550	0.61±0.02	0.77±0.04	0.52±0.03	7.69±0.04
	Leaf Photo 16 h	WEOM (Leaf, photo)	290-550	0.55±0.05	0.72±0.02	0.52±0.07	6.76±0.46
	Leaf Photo 32 h	WEOM (Leaf, photo)	290-550	0.46±0.01	0.66±0.04	0.52±0.01	6.24±0.13
	Leaf Photo 64 h	WEOM (Leaf, photo)	290-550	0.38±0.01	0.61±0.01	0.51±0.01	5.75±0.12
	Leaf Photo 96 h	WEOM (Leaf, photo)	290-550	0.30±0.02	0.55±0.04	0.51±0.01	5.45±0.21
	W16L Soil Oa Bio 0 d	WEOM (Soil, bio)	290-550	1.74±0.13	1.56±0.11	0.58±0.02	17.50±0.48
	W16L Soil Oa Bio 8 d	WEOM (Soil, bio)	290-550	3.17±0.11	2.49±0.15	0.60±0.01	24.70±3.40
	W16L Soil Oa Bio 16 d	WEOM (Soil, bio)	290-550	4.15±0.42	3.19±0.20	0.62±0.01	22.85±1.06
	W16L Soil Oa Bio 32 d	WEOM (Soil, bio)	290-550	4.32±0.36	3.36±0.26	0.63±0.03	21.53±1.10
	W16L Soil Oa Photo-Bio 0 d	WEOM (Soil, photo-bio)	290-550	0.94±0.01	1.03±0.02	0.58±0.04	12.86±0.24
	W16L Soil Oa Photo-Bio 8 d	WEOM (Soil, photo-bio)	290-550	1.43±0.06	1.35±0.07	0.59±0.01	23.24±1.24
	W16L Soil Oa Photo-Bio 16 d	WEOM (Soil, photo-bio)	290-550	1.81±0.11	1.61±0.10	0.60±0.02	22.42±0.22
	W16L Soil Oa Photo-Bio 32 d	WEOM (Soil, photo-bio)	290-550	2.15±0.09	1.87±0.15	0.61±0.03	21.61±0.15
	W24R Soil Oa Bio 0 d	WEOM (Soil, bio)	290-550	1.77±0.14	1.60±0.14	0.61±0.01	17.36±0.48
	W24R Soil Oa Bio 8 d	WEOM (Soil, bio)	290-550	3.51±0.02	2.78±0.19	0.63±0.01	31.98±1.33
	W24R Soil Oa Bio 16 d	WEOM (Soil, bio)	290-550	4.34±0.31	3.39±0.31	0.64±0.04	30.54±1.19
	W24R Soil Oa Bio 32 d	WEOM (Soil, bio)	290-550	4.61±0.23	3.57±0.27	0.65±0.05	29.49±0.22
	W24R Soil Oa Photo-Bio 0 d	WEOM (Soil, photo-bio)	290-550	1.09±0.07	1.14±0.08	0.60±0.01	12.22±0.30
	W24R Soil Oa Photo-Bio 8 d	WEOM (Soil, photo-bio)	290-550	1.63±0.11	1.51±0.14	0.61±0.01	25.70±0.84
	W24R Soil Oa Photo-Bio 16 d	WEOM (Soil, photo-bio)	290-550	1.87±0.14	1.68±0.15	0.62±0.03	25.62±1.41
	W24R Soil Oa Photo-Bio 32 d	WEOM (Soil, photo-bio)	290-550	2.38±0.18	2.03±0.15	0.63±0.01	23.74±1.94
	Leaf Bio 0 d	WEOM (Leaf, bio)	290-550	1.57±0.09	1.42±0.10	0.54±0.01	12.39±0.04
	Leaf Bio 8 d	WEOM (Leaf, bio)	290-550	3.02±0.03	2.38±0.01	0.56±0.02	30.90±2.21
	Leaf Bio 16 d	WEOM (Leaf, bio)	290-550	3.55±0.02	2.81±0.10	0.57±0.02	34.11±0.45
	Leaf Bio 32 d	WEOM (Leaf, bio)	290-550	3.97±0.03	3.10±0.01	0.58±0.02	26.61±0.57
	Leaf Photo-Bio 0 d	WEOM (Leaf, photo-bio)	290-550	0.85±0.01	0.94±0.04	0.53±0.04	9.32±0.08
	Leaf Photo-Bio 8 d	WEOM (Leaf, photo-bio)	290-550	1.49±0.06	1.36±0.05	0.56±0.01	32.51±0.28
	Leaf Photo-Bio 16 d	WEOM (Leaf, photo-bio)	290-550	2.08±0.12	1.79±0.09	0.56±0.05	29.42±1.13
	Leaf Photo-Bio 32 d	WEOM (Leaf, photo-bio)	290-550	2.36±0.19	1.98±0.15	0.57±0.05	30.57±1.51
	W16L Soil Oa Low Elevation	WEOM (Soil)	290-550	1.74±0.16	1.56±0.12	0.58±0.01	17.54±1.16

**Table S18.** Summary of literature $\Phi_{app,RI}$ data (continued)							
Source	Sample ID	Sample Classification	Wavelength Range (nm)	$\Phi_{app,\ ^3DOM^*_{TMP}}$ ($\times10^{-2}$ mol mol-photons^{-1})	$\Phi_{app,\ ^1O_2}$ ($\times10^{-2}$ mol mol-photons^{-1})	$\Phi_{app,\ ^3DOM^*_{Sorbate}}$ ($\times10^{-2}$ mol mol-photons^{-1})	$\Phi_{app,\ ^{\bullet}OH}$ ($\times10^{-6}$ mol mol-photons^{-1})
This work	W16L Soil Bs Low Elevation	WEOM (Soil)	290-550	7.06±0.73	5.97±0.44	1.93±0.01	67.63±4.11
	W16L Soil Oa Medium Elevation	WEOM (Soil)	290-550	1.76±0.11	1.57±0.09	0.59±0.01	17.78±2.04
	W16L Soil Bs Medium Elevation	WEOM (Soil)	290-550	6.86±0.85	5.78±0.87	1.92±0.05	68.32±2.72
	W16L Soil Oa High Elevation	WEOM (Soil)	290-550	1.74±0.13	1.56±0.10	0.58±0.03	17.69±2.03
	W16L Soil Bs High Elevation	WEOM (Soil)	290-550	7.20±0.68	6.12±0.69	1.94±0.01	68.59±2.59
	W24R Soil Oa Low Elevation	WEOM (Soil)	290-550	1.78±0.11	1.59±0.13	0.61±0.01	16.89±0.58
	W24R Soil Bs Low Elevation	WEOM (Soil)	290-550	7.13±0.49	6.03±0.52	1.98±0.01	65.22±2.93
	W24R Soil Oa Medium Elevation	WEOM (Soil)	290-550	1.78±0.11	1.60±0.10	0.61±0.01	16.90±2.04
	W24R Soil Bs Medium Elevation	WEOM (Soil)	290-550	7.02±0.49	5.94±0.60	1.95±0.01	65.63±3.20
	W24R Soil Oa High Elevation	WEOM (Soil)	290-550	1.77±0.14	1.60±0.09	0.61±0.01	16.94±1.90
	W24R Soil Bs High Elevation	WEOM (Soil)	290-550	6.84±0.52	5.84±0.75	1.93±0.06	65.38±2.20
	Stream ST16	Whole Water (Stream)	290-550	2.60±0.20	2.33±0.17	0.89±0.02	24.22±1.99
	Stream ST24	Whole Water (Stream)	290-550	2.70±0.25	2.39±0.16	0.89±0.02	25.00±2.11
	Honnedaga Lake	Whole Water (Lake)	290-550	2.24±0.16	1.97±0.15	0.72±0.01	23.02±2.16
	SRFA	IHSS Isolate (SRFA)	290-550	2.12±0.12	2.58±0.18	1.71±0.05	22.42±1.34
	SRHA	IHSS Isolate (SRHA)	290-550	1.55±0.07	1.47±0.01	0.64±0.06	12.87±0.91
	SRNOM	IHSS Isolate (SRNOM)	290-550	2.04±0.08	2.39±0.15	1.57±0.08	21.07±1.15
	PPFA	IHSS Isolate (PPFA)	290-550	2.10±0.05	2.57±0.08	1.68±0.06	16.94±0.99
	PPHA	IHSS Isolate (PPHA)	290-550	1.92±0.05	1.57±0.09	0.45±0.01	8.53±0.79
	NRNOM	IHSS Isolate (NRNOM)	290-550	2.26±0.18	2.78±0.10	1.89±0.04	22.78±0.72
	UMRNOM	IHSS Isolate (MRNOM)	290-550	2.18±0.03	2.75±0.22	1.96±0.03	23.21±1.27
	ESHA Photo 0 h	IHSS Isolate (ESHA, photo)	290-550	1.53±0.02	1.41±0.09	NA	14.09±1.08
	ESHA Photo 4 h	IHSS Isolate (ESHA, photo)	290-550	1.48±0.04	1.30±0.05	NA	12.31±0.92
	ESHA Photo 16 h	IHSS Isolate (ESHA, photo)	290-550	1.36±0.01	1.20±0.10	NA	10.75±0.78
	ESHA Photo 64 h	IHSS Isolate (ESHA, photo)	290-550	1.22±0.03	1.08±0.10	NA	8.56±0.81
	SRFA Photo 0 h	IHSS Isolate (SRFA, photo)	290-550	2.20±0.11	2.66±0.23	NA	24.06±1.29
	SRFA Photo 4 h	IHSS Isolate (SRFA, photo)	290-550	2.17±0.04	2.63±0.23	NA	22.23±1.26
	SRFA Photo 16 h	IHSS Isolate (SRFA, photo)	290-550	2.01±0.10	2.56±0.16	NA	19.33±1.24
	SRFA Photo 64 h	IHSS Isolate (SRFA, photo)	290-550	1.82±0.04	2.42±0.21	NA	15.26±1.29
	Glucose Bio 0 d	Glucose (bio)	290-550	0.19±0.01	0.25±0.03	0.19±0.01	8.82±0.57
	Glucose Bio 8 d	Glucose (bio)	290-550	4.52±0.19	4.22±0.31	1.96±0.20	110.91±2.26
	Glucose Bio 16 d	Glucose (bio)	290-550	6.07±0.37	5.26±0.22	1.82±0.16	203.35±12.98
	Glucose Bio 32 d	Glucose (bio)	290-550	7.19±0.55	6.36±0.39	1.93±0.21	201.69±21.10
	ESHA Bio 0 d	IHSS Isolate (ESHA, bio)	290-550	1.53±0.02	1.42±0.08	0.44±0.02	14.09±1.08
	ESHA Bio 8 d	IHSS Isolate (ESHA, bio)	290-550	1.55±0.05	1.43±0.06	0.46±0.02	25.94±0.65
	ESHA Bio 16 d	IHSS Isolate (ESHA, bio)	290-550	1.56±0.01	1.43±0.08	0.46±0.02	25.52±0.47
	ESHA Bio 32 d	IHSS Isolate (ESHA, bio)	290-550	1.56±0.07	1.44±0.07	0.46±0.02	26.07±0.78
This work	W16L Soil Oa Photo 0 h	WEOM (Soil, photo)	290-600	1.68±0.13	1.51±0.10	0.57±0.01	16.97±0.47
	W16L Soil Oa Photo 1 h	WEOM (Soil, photo)	290-600	1.01±0.02	1.06±0.03	0.56±0.03	13.53±0.31
	W16L Soil Oa Photo 2 h	WEOM (Soil, photo)	290-600	0.91±0.06	1.00±0.09	0.56±0.01	12.78±0.33
	W16L Soil Oa Photo 4 h	WEOM (Soil, photo)	290-600	0.75±0.03	0.90±0.07	0.56±0.03	11.59±0.48
	W16L Soil Oa Photo 8 h	WEOM (Soil, photo)	290-600	0.60±0.01	0.80±0.01	0.56±0.02	10.47±0.39

**Table S18.** Summary of literature $\Phi_{app,RI}$ data (continued)							
Source	Sample ID	Sample Classification	Wavelength Range (nm)	$\Phi_{app,\ ^3DOM^*_{TMP}}$ ($\times10^{-2}$ mol mol-photons^{-1})	$\Phi_{app,\ ^1O_2}$ ($\times10^{-2}$ mol mol-photons^{-1})	$\Phi_{app,\ ^3DOM^*_{Sorbate}}$ ($\times10^{-2}$ mol mol-photons^{-1})	$\Phi_{app,\ ^{\cdot}OH}$ ($\times10^{-6}$ mol mol-photons^{-1})
This work	W16L Soil Oa Photo 16 h	WEOM (Soil, photo)	290-600	0.53±0.05	0.74±0.03	0.57±0.09	10.01±0.84
	W16L Soil Oa Photo 32 h	WEOM (Soil, photo)	290-600	0.45±0.07	0.69±0.06	0.57±0.09	9.12±1.11
	W16L Soil Oa Photo 64 h	WEOM (Soil, photo)	290-600	0.37±0.02	0.64±0.03	0.56±0.04	8.47±0.35
	W16L Soil Oa Photo 96 h	WEOM (Soil, photo)	290-600	0.29±0.01	0.58±0.03	0.57±0.10	7.98±0.84
	W24R Soil Oa Photo 0 h	WEOM (Soil, photo)	290-600	1.66±0.07	1.49±0.07	0.57±0.04	16.01±0.03
	W24R Soil Oa Photo 1 h	WEOM (Soil, photo)	290-600	1.19±0.03	1.19±0.01	0.58±0.05	13.21±0.36
	W24R Soil Oa Photo 2 h	WEOM (Soil, photo)	290-600	1.07±0.06	1.12±0.09	0.59±0.01	12.26±0.55
	W24R Soil Oa Photo 4 h	WEOM (Soil, photo)	290-600	0.89±0.04	1.01±0.07	0.59±0.01	11.23±0.56
	W24R Soil Oa Photo 8 h	WEOM (Soil, photo)	290-600	0.75±0.03	0.91±0.04	0.59±0.02	10.24±0.09
	W24R Soil Oa Photo 16 h	WEOM (Soil, photo)	290-600	0.60±0.03	0.81±0.03	0.59±0.04	9.72±0.09
	W24R Soil Oa Photo 32 h	WEOM (Soil, photo)	290-600	0.52±0.02	0.76±0.03	0.58±0.02	8.72±0.22
	W24R Soil Oa Photo 64 h	WEOM (Soil, photo)	290-600	0.44±0.02	0.69±0.03	0.57±0.03	7.65±0.09
	W24R Soil Oa Photo 96 h	WEOM (Soil, photo)	290-600	0.36±0.02	0.65±0.05	0.59±0.01	6.91±0.04
	Leaf Photo 0 h	WEOM (Leaf, photo)	290-600	1.55±0.16	1.41±0.14	0.53±0.04	12.41±1.86
	Leaf Photo 1 h	WEOM (Leaf, photo)	290-600	1.03±0.11	1.05±0.06	0.52±0.02	10.82±1.39
	Leaf Photo 2 h	WEOM (Leaf, photo)	290-600	0.85±0.05	0.93±0.01	0.53±0.07	9.38±0.51
	Leaf Photo 4 h	WEOM (Leaf, photo)	290-600	0.75±0.08	0.86±0.02	0.53±0.07	8.43±0.94
	Leaf Photo 8 h	WEOM (Leaf, photo)	290-600	0.61±0.02	0.77±0.04	0.52±0.03	7.69±0.04
	Leaf Photo 16 h	WEOM (Leaf, photo)	290-600	0.55±0.05	0.72±0.02	0.52±0.07	6.76±0.46
	Leaf Photo 32 h	WEOM (Leaf, photo)	290-600	0.46±0.01	0.66±0.04	0.52±0.01	6.23±0.13
	Leaf Photo 64 h	WEOM (Leaf, photo)	290-600	0.38±0.01	0.61±0.01	0.51±0.01	5.74±0.12
	Leaf Photo 96 h	WEOM (Leaf, photo)	290-600	0.30±0.02	0.55±0.04	0.51±0.01	5.43±0.20
	W16L Soil Oa Bio 0 d	WEOM (Soil, bio)	290-600	1.68±0.12	1.51±0.11	0.57±0.02	16.96±0.46
	W16L Soil Oa Bio 8 d	WEOM (Soil, bio)	290-600	3.07±0.11	2.41±0.15	0.58±0.01	23.95±3.33
	W16L Soil Oa Bio 16 d	WEOM (Soil, bio)	290-600	4.00±0.37	3.07±0.17	0.59±0.01	22.00±0.80
	W16L Soil Oa Bio 32 d	WEOM (Soil, bio)	290-600	4.13±0.34	3.21±0.25	0.60±0.03	20.55±1.03
	W16L Soil Oa Photo-Bio 0 d	WEOM (Soil, photo-bio)	290-600	0.91±0.01	0.99±0.02	0.56±0.04	12.39±0.27
	W16L Soil Oa Photo-Bio 8 d	WEOM (Soil, photo-bio)	290-600	1.38±0.06	1.31±0.07	0.57±0.01	22.42±1.19
	W16L Soil Oa Photo-Bio 16 d	WEOM (Soil, photo-bio)	290-600	1.73±0.10	1.54±0.10	0.57±0.02	21.47±0.19
	W16L Soil Oa Photo-Bio 32 d	WEOM (Soil, photo-bio)	290-600	2.07±0.10	1.79±0.15	0.58±0.03	20.75±0.27
	W24R Soil Oa Bio 0 d	WEOM (Soil, bio)	290-600	1.73±0.14	1.56±0.14	0.60±0.01	16.99±0.45
	W24R Soil Oa Bio 8 d	WEOM (Soil, bio)	290-600	3.44±0.03	2.72±0.18	0.61±0.01	31.31±1.24
	W24R Soil Oa Bio 16 d	WEOM (Soil, bio)	290-600	4.24±0.29	3.30±0.29	0.62±0.04	29.79±1.06
	W24R Soil Oa Bio 32 d	WEOM (Soil, bio)	290-600	4.47±0.20	3.47±0.25	0.63±0.05	28.62±0.06
	W24R Soil Oa Photo-Bio 0 d	WEOM (Soil, photo-bio)	290-600	1.06±0.07	1.11±0.08	0.58±0.01	11.92±0.29
	W24R Soil Oa Photo-Bio 8 d	WEOM (Soil, photo-bio)	290-600	1.58±0.20	1.46±0.23	0.59±0.04	24.96±2.37
	W24R Soil Oa Photo-Bio 16 d	WEOM (Soil, photo-bio)	290-600	1.81±0.20	1.63±0.21	0.60±0.05	24.80±2.33
	W24R Soil Oa Photo-Bio 32 d	WEOM (Soil, photo-bio)	290-600	2.31±0.21	1.97±0.17	0.61±0.01	23.02±2.21
	Leaf Bio 0 d	WEOM (Leaf, bio)	290-600	1.53±0.08	1.38±0.10	0.52±0.01	12.03±0.08
	Leaf Bio 8 d	WEOM (Leaf, bio)	290-600	2.98±0.01	2.35±0.04	0.55±0.02	30.50±1.80
	Leaf Bio 16 d	WEOM (Leaf, bio)	290-600	3.45±0.02	2.72±0.09	0.55±0.02	33.08±0.42
	Leaf Bio 32 d	WEOM (Leaf, bio)	290-600	3.79±0.02	2.96±0.03	0.55±0.01	25.38±0.22

Table S18. Summary of literature $\Phi_{app,RI}$ data (continued)							
Source	Sample ID	Sample Classification	Wavelength Range (nm)	$\Phi_{app,\ ^3DOM^*_{TMP}}$ ($\times10^{-2}$ mol mol-photons^{-1})	$\Phi_{app,\ ^1O_2}$ ($\times10^{-2}$ mol mol-photons^{-1})	$\Phi_{app,\ ^3DOM^*_{Sorbate}}$ ($\times10^{-2}$ mol mol-photons^{-1})	$\Phi_{app,\ ^{\bullet}OH}$ ($\times10^{-6}$ mol mol-photons^{-1})
This work	Leaf Photo-Bio 0 d	WEOM (Leaf, photo-bio)	290-600	0.82±0.01	0.91±0.04	0.51±0.04	9.02±0.11
	Leaf Photo-Bio 8 d	WEOM (Leaf, photo-bio)	290-600	1.44±0.06	1.32±0.05	0.54±0.01	31.59±0.24
	Leaf Photo-Bio 16 d	WEOM (Leaf, photo-bio)	290-600	2.01±0.11	1.73±0.08	0.54±0.04	28.50±0.98
	Leaf Photo-Bio 32 d	WEOM (Leaf, photo-bio)	290-600	2.29±0.18	1.93±0.14	0.55±0.05	29.67±1.41
	W16L Soil Oa Low Elevation	WEOM (Soil)	290-600	1.63±0.15	1.46±0.11	0.55±0.01	16.44±1.09
	W16L Soil Bs Low Elevation	WEOM (Soil)	290-600	6.94±0.73	5.87±0.43	1.89±0.01	66.50±4.02
	W16L Soil Oa Medium Elevation	WEOM (Soil)	290-600	1.72±0.11	1.53±0.09	0.57±0.01	17.34±1.99
	W16L Soil Bs Medium Elevation	WEOM (Soil)	290-600	6.68±0.83	5.64±0.85	1.87±0.05	66.59±2.63
	W16L Soil Oa High Elevation	WEOM (Soil)	290-600	1.67±0.12	1.50±0.09	0.56±0.03	17.02±1.95
	W16L Soil Bs High Elevation	WEOM (Soil)	290-600	7.18±0.68	6.11±0.69	1.94±0.01	68.46±2.55
	W24R Soil Oa Low Elevation	WEOM (Soil)	290-600	1.78±0.11	1.59±0.13	0.60±0.01	16.86±0.58
	W24R Soil Bs Low Elevation	WEOM (Soil)	290-600	7.02±0.49	5.94±0.51	1.95±0.01	64.23±2.88
	W24R Soil Oa Medium Elevation	WEOM (Soil)	290-600	1.74±0.11	1.56±0.10	0.60±0.01	16.53±2.00
	W24R Soil Bs Medium Elevation	WEOM (Soil)	290-600	7.01±0.48	5.94±0.60	1.95±0.01	65.55±3.18
	W24R Soil Oa High Elevation	WEOM (Soil)	290-600	1.69±0.14	1.53±0.09	0.58±0.01	16.18±1.82
	W24R Soil Bs High Elevation	WEOM (Soil)	290-600	6.57±0.50	5.60±0.72	1.86±0.06	62.77±2.10
	Stream ST16	Whole Water (Stream)	290-600	2.57±0.20	2.30±0.16	0.88±0.02	23.93±1.97
	Stream ST24	Whole Water (Stream)	290-600	2.70±0.25	2.39±0.16	0.89±0.02	24.99±2.10
	Honnedaga Lake	Whole Water (Lake)	290-600	2.22±0.16	1.95±0.15	0.72±0.01	22.86±2.15
	SRFA	IHSS Isolate (SRFA)	290-600	2.06±0.14	2.51±0.19	1.64±0.05	21.52±1.29
	SRHA	IHSS Isolate (SRHA)	290-600	1.49±0.06	1.41±0.01	0.61±0.06	12.36±0.88
	SRNOM	IHSS Isolate (SRNOM)	290-600	1.98±0.08	2.32±0.14	1.53±0.08	20.42±1.11
	PPFA	IHSS Isolate (PPFA)	290-600	2.01±0.05	2.46±0.08	1.61±0.06	16.24±0.95
	PPHA	IHSS Isolate (PPHA)	290-600	1.80±0.05	1.46±0.09	0.42±0.01	7.96±0.74
	NRNOM	IHSS Isolate (NRNOM)	290-600	2.12±0.16	2.61±0.09	1.78±0.04	21.39±0.69
	UMRNOM	IHSS Isolate (MRNOM)	290-600	2.10±0.03	2.64±0.21	1.88±0.02	22.30±1.22
	ESHA Photo 0 h	IHSS Isolate (ESHA, photo)	290-600	1.39±0.02	1.29±0.08	NA	12.87±0.99
	ESHA Photo 4 h	IHSS Isolate (ESHA, photo)	290-600	1.36±0.04	1.19±0.04	NA	11.28±0.78
	ESHA Photo 16 h	IHSS Isolate (ESHA, photo)	290-600	1.26±0.01	1.12±0.09	NA	9.97±0.71
	ESHA Photo 64 h	IHSS Isolate (ESHA, photo)	290-600	1.13±0.03	1.00±0.09	NA	7.93±0.71
	SRFA Photo 0 h	IHSS Isolate (SRFA, photo)	290-600	2.17±0.11	2.62±0.23	NA	23.67±1.28
	SRFA Photo 4 h	IHSS Isolate (SRFA, photo)	290-600	2.13±0.04	2.58±0.23	NA	21.83±1.23
	SRFA Photo 16 h	IHSS Isolate (SRFA, photo)	290-600	1.98±0.10	2.51±0.15	NA	18.99±1.18
	SRFA Photo 64 h	IHSS Isolate (SRFA, photo)	290-600	1.79±0.05	2.38±0.19	NA	14.99±1.16
	Glucose Bio 0 d	Glucose (bio)	290-600	0.17±0.01	0.23±0.02	0.18±0.01	8.17±0.21
	Glucose Bio 8 d	Glucose (bio)	290-600	4.02±0.17	3.75±0.27	1.74±0.19	98.56±3.03
	Glucose Bio 16 d	Glucose (bio)	290-600	5.64±0.45	4.88±0.17	1.69±0.19	188.98±17.56
	Glucose Bio 32 d	Glucose (bio)	290-600	7.00±0.51	6.19±0.38	1.88±0.19	196.28±19.53
	ESHA Bio 0 d	IHSS Isolate (ESHA, bio)	290-600	1.40±0.02	1.29±0.07	0.41±0.02	12.87±0.98
	ESHA Bio 8 d	IHSS Isolate (ESHA, bio)	290-600	1.44±0.05	1.33±0.06	0.42±0.02	24.03±0.57
	ESHA Bio 16 d	IHSS Isolate (ESHA, bio)	290-600	1.44±0.01	1.33±0.08	0.43±0.01	23.62±0.43
	ESHA Bio 32 d	IHSS Isolate (ESHA, bio)	290-600	1.45±0.06	1.33±0.06	0.43±0.02	24.17±0.69

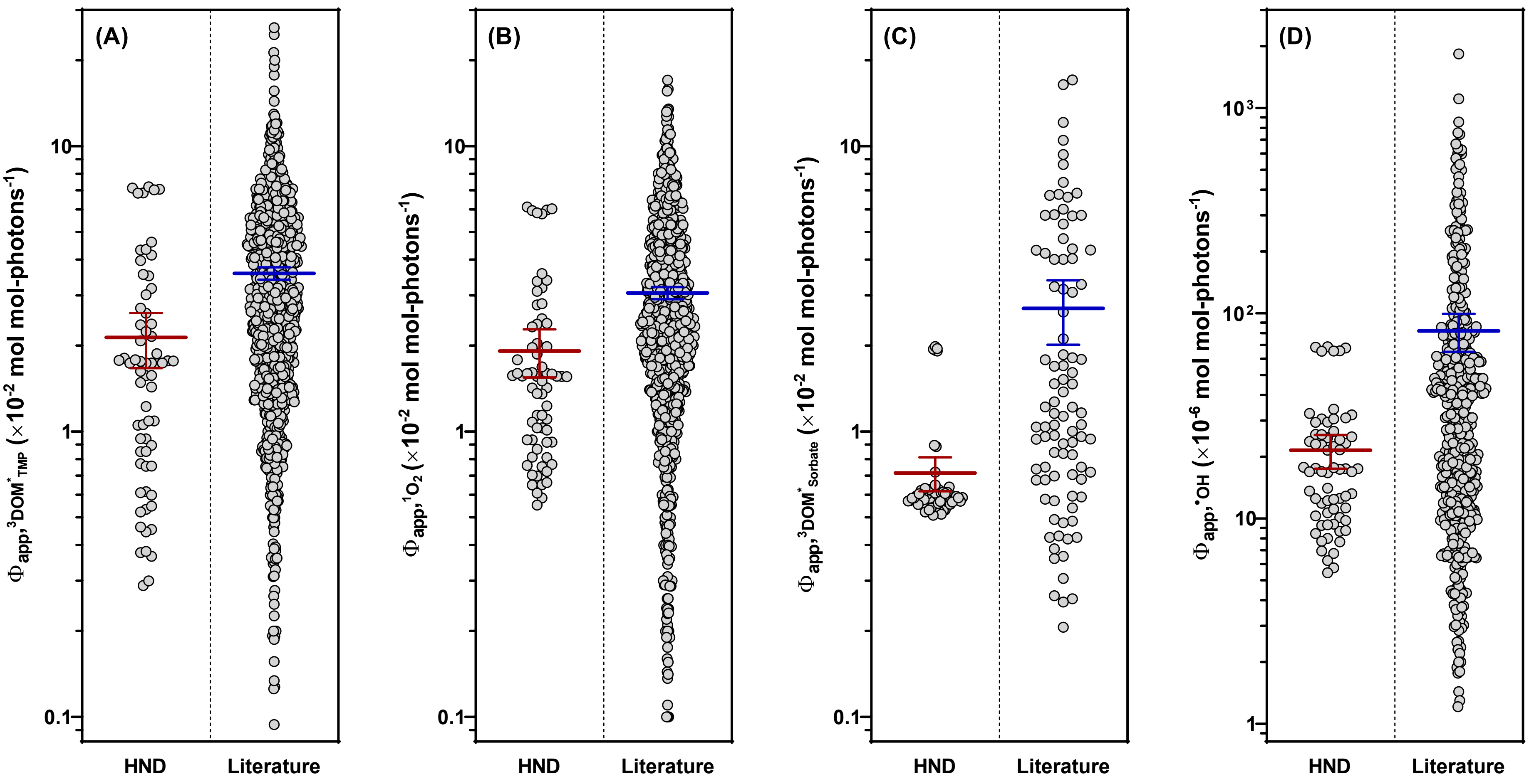

Figure S11. Comparison of $\Phi_{app,RI}$ for native, photodegraded, and (photo-)biodegraded Honnedaga leachates and whole water samples (HND; *n*=66) with those for whole waters and DOM isolates reported in the literature (i.e., $\Phi_{app,RI}$ summarized in Table S18 plus $\Phi_{app,RI}$ summarized in Wasswa *et al.*[4] Table S16): **(A)** Scatter dot plot of $\Phi_{app,\ {}^3DOM^*_{TMP}}$ for Honnedaga samples (red centerline at mean with 95% confidence interval) and $\Phi_{app,\ {}^3DOM^*_{TMP}}$ reported in the literature (blue centerline at mean with 95% confidence interval; *n*=1063). **(B)** Scatter dot plot of $\Phi_{app,\ {}^1O_2}$ for Honnedaga samples and $\Phi_{app,\ {}^1O_2}$ reported in the literature (*n*=997). **(C)** Scatter dot plot of $\Phi_{app,\ {}^3DOM^*_{HDO}}$ for Honnedaga samples and $\Phi_{app,\ {}^3DOM^*_{Sorbate}}$ (i.e., the Φ_{app} of high-energy $^3DOM^*$ measured by sorbate probes such as sorbic acid, sorbic alcohol, and sorbic amine) reported in the literature (*n*=92). **(D)** Scatter dot plot of $\Phi_{app,\ \cdot OH}$ for Honnedaga samples and $\Phi_{app,\ \cdot OH}$ reported in the literature (*n*=548). Note that Table S18 complements Table S16 in Wasswa *et al.*[4] $\Phi_{app,RI}$ values not directly reported in the references or associated supplementary documents were digitized from raw figures using *Plot Digitizer 2.6.8*. f_{TMP} were converted to $\Phi_{app,\ {}^3DOM^*_{TMP}}$ using $k_{TMP,\ {}^3DOM^*_{TMP}}$ values reported in Erickson *et al.*[90] when applicable.

References

1. Armarego, W. L. F., *Purification of Laboratory Chemicals*. 8th ed.; Butterworth-Heinemann: Cambridge, MA, 2017.
2. Dulin, D.; Mill, T., Development and evaluation of sunlight actinometers. *Environmental Science & Technology* **1982,** *16*, (11), 815-820.
3. Laszakovits, J. R.; Berg, S. M.; Anderson, B. G.; O'Brien, J. E.; Wammer, K. H.; Sharpless, C. M., *p*-Nitroanisole/pyridine and *p*-nitroacetophenone/pyridine actinometers revisited: Quantum yield in comparison to ferrioxalate. *Environmental Science & Technology Letters* **2017,** *4*, (1), 11-14.
4. Wasswa, J.; Driscoll, C. T.; Zeng, T., Photochemical characterization of surface waters from lakes in the Adirondack Region of New York. *Environmental Science & Technology* **2020,** *54*, (17), 10654-10667.
5. Cory, R. M.; Miller, M. P.; McKnight, D. M.; Guerard, J. J.; Miller, P. L., Effect of instrument-specific response on the analysis of fulvic acid fluorescence spectra. *Limnology and Oceanography: Methods* **2010,** *8*, (2), 67-78.
6. Ohno, T., Fluorescence inner-filtering correction for determining the humification index of dissolved organic matter. *Environmental Science & Technology* **2002,** *36*, (4), 742-746.
7. Kothawala, D. N.; Murphy, K. R.; Stedmon, C. A.; Weyhenmeyer, G. A.; Tranvik, L. J., Inner filter correction of dissolved organic matter fluorescence. *Limnology and Oceanography: Methods* **2013,** *11*, (12), 616-630.
8. Lawaetz, A. J.; Stedmon, C. A., Fluorescence intensity calibration using the Raman scatter peak of water. *Applied Spectroscopy* **2009,** *63*, (8), 936-940.
9. Murphy, K. R.; Stedmon, C. A.; Graeber, D.; Bro, R., Fluorescence spectroscopy and multi-way techniques. PARAFAC. *Analytical Methods* **2013,** *5*, (23), 6557-6566.
10. Green, S. A.; Blough, N. V., Optical absorption and fluorescence properties of chromophoric dissolved organic matter in natural waters. *Limnology and Oceanography* **1994,** *39*, (8), 1903-1916.
11. Hudson, N.; Baker, A.; Reynolds, D., Fluorescence analysis of dissolved organic matter in natural, waste and polluted waters—a review. *River Research and Applications* **2007,** *23*, (6), 631-649.
12. Fellman, J. B.; Hood, E.; Spencer, R. G. M., Fluorescence spectroscopy opens new windows into dissolved organic matter dynamics in freshwater ecosystems: A review. *Limnology and Oceanography* **2010,** *55*, (6), 2452-2462.
13. Hansen, A. M.; Kraus, T. E. C.; Pellerin, B. A.; Fleck, J. A.; Downing, B. D.; Bergamaschi, B. A., Optical properties of dissolved organic matter (DOM): Effects of biological and photolytic degradation. *Limnology and Oceanography* **2016,** *61*, (3), 1015-1032.
14. Weishaar, J. L.; Aiken, G. R.; Bergamaschi, B. A.; Fram, M. S.; Fujii, R.; Mopper, K., Evaluation of specific ultraviolet absorbance as an indicator of the chemical composition and reactivity of dissolved organic carbon. *Environmental Science & Technology* **2003,** *37*, (20), 4702-4708.
15. De Haan, H.; De Boer, T., Applicability of light absorbance and fluorescence as measures of concentration and molecular size of dissolved organic carbon in humic Lake Tjeukemeer. *Water Research* **1987,** *21*, (6), 731-734.
16. Moran, M. A.; Sheldon, W. M., Jr.; Zepp, R. G., Carbon loss and optical property changes during long-term photochemical and biological degradation of estuarine dissolved organic matter. *Limnology and Oceanography* **2000,** *45*, (6), 1254-1264.
17. Twardowski, M. S.; Boss, E.; Sullivan, J. M.; Donaghay, P. L., Modeling the spectral shape of absorption by chromophoric dissolved organic matter. *Marine Chemistry* **2004,** *89*, (1), 69-88.
18. Helms, J. R.; Stubbins, A.; Ritchie, J. D.; Minor, E. C.; Kieber, D. J.; Mopper, K., Absorption spectral slopes and slope ratios as indicators of molecular weight, source, and photobleaching of chromophoric dissolved organic matter. *Limnology and Oceanography* **2008,** *53*, (3), 955-969.
19. McKnight, D. M.; Boyer, E. W.; Westerhoff, P. K.; Doran, P. T.; Kulbe, T.; Andersen, D. T., Spectrofluorometric characterization of dissolved organic matter for indication of precursor organic material and aromaticity. *Limnology and Oceanography* **2001,** *46*, (1), 38-48.

20. Cory, R. M.; McKnight, D. M., Fluorescence spectroscopy reveals ubiquitous presence of oxidized and reduced quinones in dissolved organic matter. *Environmental Science & Technology* **2005,** *39*, (21), 8142-8149.
21. Zsolnay, A.; Baigar, E.; Jimenez, M.; Steinweg, B.; Saccomandi, F., Differentiating with fluorescence spectroscopy the sources of dissolved organic matter in soils subjected to drying. *Chemosphere* **1999,** *38*, (1), 45-50.
22. Halbedel, S.; Herzsprung, P., Short communication on "Differentiating with fluorescence spectroscopy the sources of dissolved organic matter in soils subjected to drying" [Zsolnay, A.; Baigar, E.; Jimenez, M.; Steinweg, B.; Saccomandi, F.; Chemosphere 38, 45–50, 1999]. *Chemosphere* **2020,** *239*, Article Number: 124818.
23. Parlanti, E.; Wörz, K.; Geoffroy, L.; Lamotte, M., Dissolved organic matter fluorescence spectroscopy as a tool to estimate biological activity in a coastal zone submitted to anthropogenic inputs. *Organic Geochemistry* **2000,** *31*, (12), 1765-1781.
24. Wilson, H. F.; Xenopoulos, M. A., Effects of agricultural land use on the composition of fluvial dissolved organic matter. *Nature Geoscience* **2008,** *2*, 37-41.
25. Huguet, A.; Vacher, L.; Relexans, S.; Saubusse, S.; Froidefond, J. M.; Parlanti, E., Properties of fluorescent dissolved organic matter in the Gironde Estuary. *Organic Geochemistry* **2009,** *40*, (6), 706-719.
26. Miller, M. P.; McKnight, D. M.; Cory, R. M.; Williams, M. W.; Runkel, R. L., Hyporheic exchange and fulvic acid redox reactions in an alpine stream/wetland ecosystem, Colorado Front Range. *Environmental Science & Technology* **2006,** *40*, (19), 5943-5949.
27. Murphy, K. R.; Stedmon, C. A.; Wenig, P.; Bro, R., OpenFluor- an online spectral library of auto-fluorescence by organic compounds in the environment. *Analytical Methods* **2014,** *6*, (3), 658-661.
28. Lambert, T.; Bouillon, S.; Darchambeau, F.; Massicotte, P.; Borges, A. V., Shift in the chemical composition of dissolved organic matter in the Congo River network. *Biogeosciences* **2016,** *13*, (18), 5405-5420.
29. Hambly, A. C.; Arvin, E.; Pedersen, L. F.; Pedersen, P. B.; Seredyńska-Sobecka, B.; Stedmon, C. A., Characterising organic matter in recirculating aquaculture systems with fluorescence EEM spectroscopy. *Water Research* **2015,** *83*, 112-120.
30. Osburn, C. L.; Handsel, L. T.; Peierls, B. L.; Paerl, H. W., Predicting sources of dissolved organic nitrogen to an estuary from an agro-urban coastal watershed. *Environmental Science & Technology* **2016,** *50*, (16), 8473-8484.
31. Murphy, K. R.; Hambly, A.; Singh, S.; Henderson, R. K.; Baker, A.; Stuetz, R.; Khan, S. J., Organic matter fluorescence in municipal water recycling schemes: Toward a unified PARAFAC model. *Environmental Science & Technology* **2011,** *45*, (7), 2909-2916.
32. Re, R.; Pellegrini, N.; Proteggente, A.; Pannala, A.; Yang, M.; Rice-Evans, C., Antioxidant activity applying an improved ABTS radical cation decolorization assay. *Free Radical Biology and Medicine* **1999,** *26*, (9), 1231-1237.
33. Rimmer, D. L.; Abbott, G. D., Phenolic compounds in NaOH extracts of UK soils and their contribution to antioxidant capacity. *European Journal of Soil Science* **2011,** *62*, (2), 285-294.
34. Walpen, N.; Schroth, M. H.; Sander, M., Quantification of phenolic antioxidant moieties in dissolved organic matter by flow-injection analysis with electrochemical detection. *Environmental Science & Technology* **2016,** *50*, (12), 6423-6432.
35. Singleton, V. L.; Orthofer, R.; Lamuela-Raventós, R. M., Analysis of total phenols and other oxidation substrates and antioxidants by means of Folin-Ciocalteu reagent. In *Methods in Enzymology*, Academic Press: 1999; Vol. 299, pp 152-178.
36. Ainsworth, E. A.; Gillespie, K. M., Estimation of total phenolic content and other oxidation substrates in plant tissues using Folin–Ciocalteu reagent. *Nature Protocols* **2007,** *2*, (4), 875-877.
37. Kaiser, K.; Benner, R., Characterization of lignin by gas chromatography and mass spectrometry using a simplified CuO oxidation method. *Analytical Chemistry* **2012,** *84*, (1), 459-464.
38. Haag, W. R.; Hoigné, J.; Gassman, E.; Braun, A. M., Singlet oxygen in surface waters - Part I: Furfuryl alcohol as a trapping agent. *Chemosphere* **1984,** *13*, (5-6), 631-640.

39. Appiani, E.; Ossola, R.; Latch, D. E.; Erickson, P. R.; McNeill, K., Aqueous singlet oxygen reaction kinetics of furfuryl alcohol: Effect of temperature, pH, and salt content. *Environmental Science: Processes & Impacts* **2017,** *19*, (4), 507-516.
40. Canonica, S.; Freiburghaus, M., Electron-rich phenols for probing the photochemical reactivity of freshwaters. *Environmental Science & Technology* **2001,** *35*, (4), 690-695.
41. McCabe, A. J.; Arnold, W. A., Multiple linear regression models to predict the formation efficiency of triplet excited states of dissolved organic matter in temperate wetlands. *Limnology and Oceanography* **2018,** *63*, (5), 1992-2014.
42. Zhou, H.; Yan, S.; Ma, J.; Lian, L.; Song, W., Development of novel chemical probes for examining triplet natural organic matter under solar illumination. *Environmental Science & Technology* **2017,** *51*, (19), 11066-11074.
43. Leifer, A., *The Kinetics of Environmental Aquatic Photochemistry: Theory and Practice*. American Chemical Society: Washington, DC, 1988.
44. Schwarzenbach, R. P.; Gschwend, P. M.; Imboden, D. M., *Environmental Organic Chemistry*. 3rd Edition ed.; John Wiley & Sons, Inc.: Hoboken, NJ, 2016.
45. Partanen, S. B.; Erickson, P. R.; Latch, D. E.; Moor, K. J.; McNeill, K., Dissolved organic matter singlet oxygen quantum yields: Evaluation using time-resolved singlet oxygen phosphorescence. *Environmental Science & Technology* **2020,** *54*, (6), 3316-3324.
46. Wu, B.; Liu, T.; Wang, Y.; Zhao, G.; Chen, B.; Chu, C., High sample throughput LED reactor for facile characterization of the quantum yield spectrum of photochemically produced reactive intermediates. *Environmental Science & Technology* **2021**, DOI: 10.1021/acs.est.1c04608.
47. O'Connor, M.; Helal, S. R.; Latch, D. E.; Arnold, W. A., Quantifying photo-production of triplet excited states and singlet oxygen from effluent organic matter. *Water Research* **2019,** *156*, 23-33.
48. Sharpless, C. M.; Aeschbacher, M.; Page, S. E.; Wenk, J.; Sander, M.; McNeill, K., Photooxidation-induced changes in optical, electrochemical, and photochemical properties of humic substances. *Environmental Science & Technology* **2014,** *48*, (5), 2688-2696.
49. McCabe, A. J.; Arnold, W. A., Seasonal and spatial variabilities in the water chemistry of prairie pothole wetlands influence the photoproduction of reactive intermediates. *Chemosphere* **2016,** *155*, 640-647.
50. Pochon, A.; Vaughan, P. P.; Gan, D.; Vath, P.; Blough, N. V.; Falvey, D. E., Photochemical oxidation of water by 2-methyl-1, 4-benzoquinone: Evidence against the formation of free hydroxyl radical. *The Journal of Physical Chemistry A* **2002,** *106*, (12), 2889-2894.
51. Gan, D.; Jia, M.; Vaughan, P. P.; Falvey, D. E.; Blough, N. V., Aqueous photochemistry of methyl-benzoquinone. *The Journal of Physical Chemistry A* **2008,** *112*, (13), 2803-2812.
52. Page, S. E.; Arnold, W. A.; McNeill, K., Assessing the contribution of free hydroxyl radical in organic matter-sensitized photohydroxylation reactions. *Environmental Science & Technology* **2011,** *45*, (7), 2818-2825.
53. Liu, Y.; Yan, S.; Lian, L.; Ma, J.; Zhou, H.; Song, W., Assessing the contribution of hydroxylation species in the photochemical transformation of primidone (pharmaceutical). *Science of the Total Environment* **2019,** *696*, 133826.
54. Page, S. E.; Arnold, W. A.; McNeill, K., Terephthalate as a probe for photochemically generated hydroxyl radical. *Journal of Environmental Monitoring* **2010,** *12*, 1658-1665.
55. Yuan, C.; Chin, Y.-P.; Weavers, L. K., Photochemical acetochlor degradation induced by hydroxyl radical in Fe-amended wetland waters: Impact of pH and dissolved organic matter. *Water Research* **2018,** *132*, 52-60.
56. Charbouillot, T.; Brigante, M.; Mailhot, G.; Maddigapu, P. R.; Minero, C.; Vione, D., Performance and selectivity of the terephthalic acid probe for $^{\bullet}OH$ as a function of temperature, pH and composition of atmospherically relevant aqueous media. *Journal of Photochemistry and Photobiology A: Chemistry* **2011,** *222*, (1), 70-76.
57. Miller, P. L.; Chin, Y.-P., Photoinduced degradation of carbaryl in a wetland surface water. *Journal of Agricultural and Food Chemistry* **2002,** *50*, (23), 6758-6765.

58. Yuan, C.; Sleighter, R. L.; Weavers, L. K.; Hatcher, P. G.; Chin, Y.-P., Fast photomineralization of dissolved organic matter in acid mine drainage impacted waters. *Environmental Science & Technology* **2019,** *53*, (11), 6273-6281.
59. Matthews, R. W., The radiation-chemistry of the terephthalate dosimeter. *Radiation Research* **1980,** *83*, (1), 27-41.
60. Mark, G.; Tauber, A.; Laupert, R.; Schuchmann, H.-P.; Schulz, D.; Mues, A.; von Sonntag, C., OH-radical formation by ultrasound in aqueous solution – Part II: Terephthalate and Fricke dosimetry and the influence of various conditions on the sonolytic yield. *Ultrasonics Sonochemistry* **1998,** *5*, (2), 41-52.
61. Gonzalez, D. H.; Kuang, X. M.; Scott, J. A.; Rocha, G. O.; Paulson, S. E., Terephthalate probe for hydroxyl radicals: Yield of 2-hydroxyterephthalic acid and transition metal interference. *Analytical Letters* **2018,** *51*, (15), 2488-2497.
62. Buxton, G. V.; Greenstock, C. L.; Helman, W. P.; Ross, A. B., Critical review of rate constants for reactions of hydrated electrons and hydroxyl radicals ($^{\bullet}OH/^{\bullet}O^{-}$) in aqueous solution. *Journal of Physical and Chemical Reference Data* **1988,** *17*, (2), 513-886.
63. Zepp, R. G.; Faust, B. C.; Hoigné, J., Hydroxyl radical formation in aqueous reactions (pH 3-8) of iron(II) with hydrogen peroxide: The photo-Fenton reaction. *Environmental Science & Technology* **1992,** *26*, (2), 313-319.
64. Westerhoff, P.; Song, R.; Amy, G.; Minear, R., NOM's role in bromine and bromate formation during ozonation. *Journal - American Water Works Association* **1998,** *90*, (2), 82-94.
65. Brezonik, P. L.; Fulkerson-Brekken, J., Nitrate-induced photolysis in natural waters: Controls on concentrations of hydroxyl radical photo-intermediates by natural scavenging agents. *Environmental Science & Technology* **1998,** *32*, (19), 3004-3010.
66. Westerhoff, P.; Aiken, G.; Amy, G.; Debroux, J., Relationships between the structure of natural organic matter and its reactivity towards molecular ozone and hydroxyl radicals. *Water Research* **1999,** *33*, (10), 2265-2276.
67. Goldstone, J. V.; Pullin, M. J.; Bertilsson, S.; Voelker, B. M., Reactions of hydroxyl radical with humic substances: Bleaching, mineralization, and production of bioavailable carbon substrates. *Environmental Science & Technology* **2002,** *36*, (3), 364-372.
68. Southworth, B. A.; Voelker, B. M., Hydroxyl radical production via the photo-Fenton reaction in the presence of fulvic acid. *Environmental Science & Technology* **2003,** *37*, (6), 1130-1136.
69. Westerhoff, P.; Mezyk, S. P.; Cooper, W. J.; Minakata, D., Electron pulse radiolysis determination of hydroxyl radical rate constants with Suwannee River fulvic acid and other dissolved organic matter isolates. *Environmental Science & Technology* **2007,** *41*, (13), 4640-4646.
70. McKay, G.; Kleinman, J.; Johnston, K.; Dong, M.; Rosario-Ortiz, F.; Mezyk, S., Kinetics of the reaction between the hydroxyl radical and organic matter standards from the International Humic Substance Society. *Journal of Soils and Sediments* **2014,** *14*, (2), 298-304.
71. Appiani, E.; Page, S. E.; McNeill, K., On the use of hydroxyl radical kinetics to assess the number-average molecular weight of dissolved organic matter. *Environmental Science & Technology* **2014,** *48*, (20), 11794-11802.
72. Page, S. E.; Logan, J. R.; Cory, R. M.; McNeill, K., Evidence for dissolved organic matter as the primary source and sink of photochemically produced hydroxyl radical in arctic surface waters. *Environmental Science: Processes & Impacts* **2014,** *16*, (4), 807-822.
73. Vermilyea, A. W.; Voelker, B. M., Photo-Fenton reaction at near neutral pH. *Environmental Science & Technology* **2009,** *43*, (18), 6927-6933.
74. Ossola, R.; Jönsson, O. M.; Moor, K.; McNeill, K., Singlet oxygen quantum yields in environmental waters. *Chemical Reviews* **2021,** *121*, (7), 4100-4146.
75. Manfrin, A.; Nizkorodov, S. A.; Malecha, K. T.; Getzinger, G. J.; McNeill, K.; Borduas-Dedekind, N., Reactive oxygen species production from secondary organic aerosols: The importance of singlet oxygen. *Environmental Science & Technology* **2019,** *53*, (15), 8553-8562.

76. Mostafa, S.; Rosario-Ortiz, F. L., Singlet oxygen formation from wastewater organic matter. *Environmental Science & Technology* **2013,** *47*, (15), 8179-8186.
77. Dalrymple, R. e. M.; Carfagno, A. K.; Sharpless, C. M., Correlations between dissolved organic matter optical properties and quantum yields of singlet oxygen and hydrogen peroxide. *Environmental Science & Technology* **2010,** *44*, (15), 5824-5829.
78. Zepp, R. G.; Schlotzhauer, P. F.; Sink, R. M., Photosensitized transformations involving electronic energy transfer in natural waters: Role of humic substances. *Environmental Science & Technology* **1985,** *19*, (1), 74-81.
79. Zhou, H.; Yan, S.; Lian, L.; Song, W., Triplet-state photochemistry of dissolved organic matter: Triplet-state energy distribution and surface electric charge conditions. *Environmental Science & Technology* **2019,** *53*, (5), 2482-2490.
80. Aguer, J.-P.; Mailhot, G.; Bolte, M., Unexpected 2,4,6-trimethylphenol oxidation in the presence of Fe(III) aquacomplexes. *New Journal of Chemistry* **2006,** *30*, (2), 191-196.
81. Canonica, S.; Jans, U.; Stemmler, K.; Hoigné, J., Transformation kinetics of phenols in water: Photosensitization by dissolved natural organic material and aromatic ketones. *Environmental Science & Technology* **1995,** *29*, (7), 1822-1831.
82. McCabe, A. J.; Arnold, W. A., Reactivity of triplet excited states of dissolved natural organic matter in stormflow from mixed-use watersheds. *Environmental Science & Technology* **2017,** *51*, (17), 9718-9728.
83. Aguer, J.-P.; Tetegan, D.; Richard, C., Humic substances mediated phototransformation of 2,4,6-trimethylphenol: A catalytic reaction. *Photochemical & Photobiological Sciences* **2005,** *4*, (6), 451-453.
84. Faust, B. C.; Hoigné, J., Sensitized photooxidation of phenols by fulvic acid and in natural waters. *Environmental Science & Technology* **1987,** *21*, (10), 957-964.
85. Halladja, S.; ter Halle, A.; Aguer, J.-P.; Boulkamh, A.; Richard, C., Inhibition of humic substances mediated photooxygenation of furfuryl alcohol by 2,4,6-trimethylphenol. Evidence for reactivity of the phenol with humic triplet excited states. *Environmental Science & Technology* **2007,** *41*, (17), 6066-6073.
86. Chin, Y.-P.; Miller, P. L.; Zeng, L.; Cawley, K.; Weavers, L. K., Photosensitized degradation of bisphenol A by dissolved organic matter. *Environmental Science & Technology* **2004,** *38*, (22), 5888-5894.
87. Bodhipaksha, L. C.; Sharpless, C. M.; Chin, Y.-P.; Sander, M.; Langston, W. K.; MacKay, A. A., Triplet photochemistry of effluent and natural organic matter in whole water and isolates from effluent-receiving rivers. *Environmental Science & Technology* **2015,** *49*, (6), 3453-3463.
88. Stirchak, L. T.; Moor, K. J.; McNeill, K.; Donaldson, D. J., Differences in photochemistry between seawater and freshwater for two natural organic matter samples. *Environmental Science: Processes & Impacts* **2019,** *21*, (1), 28-39.
89. Tenorio, R.; Fedders, A. C.; Strathmann, T. J.; Guest, J. S., Impact of growth phases on photochemically produced reactive species in the extracellular matrix of algal cultivation systems. *Environmental Science: Water Research & Technology* **2017,** *3*, (6), 1095-1108.
90. Erickson, P. R.; Moor, K. J.; Werner, J. J.; Latch, D. E.; Arnold, W. A.; McNeill, K., Singlet oxygen phosphorescence as a probe for triplet-state dissolved organic matter reactivity. *Environmental Science & Technology* **2018,** *52*, (16), 9170-9178.
91. Kroflič, A.; Schaefer, T.; Huš, M.; Phuoc Le, H.; Otto, T.; Herrmann, H., OH radicals reactivity towards phenol-related pollutants in water: Temperature dependence of the rate constants and novel insights into the [OH–phenol]$^{\cdot}$ adduct formation. *Physical Chemistry Chemical Physics* **2020,** *22*, (3), 1324-1332.
92. Canonica, S.; Hellrung, B.; Wirz, J., Oxidation of phenols by triplet aromatic ketones in aqueous solution. *The Journal of Physical Chemistry A* **2000,** *104*, (6), 1226-1232.
93. Canonica, S.; Laubscher, H. U., Inhibitory effect of dissolved organic matter on triplet-induced oxidation of aquatic contaminants. *Photochemical and Photobiological Sciences* **2008,** *7*, (5), 547-551.
94. Wenk, J.; von Gunten, U.; Canonica, S., Effect of dissolved organic matter on the transformation of contaminants induced by excited triplet states and the hydroxyl radical. *Environmental Science & Technology* **2011,** *45*, (4), 1334-1340.

95. Wenk, J.; Canonica, S., Phenolic antioxidants inhibit the triplet-induced transformation of anilines and sulfonamide antibiotics in aqueous solution. *Environmental Science & Technology* **2012,** *46*, (10), 5455-5462.
96. Wenk, J.; Eustis, S. N.; McNeill, K.; Canonica, S., Quenching of excited triplet states by dissolved natural organic matter. *Environmental Science & Technology* **2013,** *47*, (22), 12802-12810.
97. Maizel, A. C.; Remucal, C. K., The effect of probe choice and solution conditions on the apparent photoreactivity of dissolved organic matter. *Environmental Science: Processes & Impacts* **2017,** *19*, (8), 1040-1050.
98. Baird, R. B.; Eaton, A. D.; Rice, E. W., *Standard Methods For the Examination of Water and Wastewater*. 23rd ed.; American Public Health Association (APHA), American Water Works Association (AWWA), Water Environment Federation (WEF): Washington, D.C., 2017.
99. Al Housari, F.; Vione, D.; Chiron, S.; Barbati, S., Reactive photoinduced species in estuarine waters. Characterization of hydroxyl radical, singlet oxygen and dissolved organic matter triplet state in natural oxidation processes. *Photochemical and Photobiological Sciences* **2010,** *9*, (1), 78-86.
100. Golanoski, K. S.; Fang, S.; Del Vecchio, R.; Blough, N. V., Investigating the mechanism of phenol photooxidation by humic substances. *Environmental Science & Technology* **2012,** *46*, (7), 3912-3920.
101. Wenk, J.; Nguyen, M. T.; Nelson, K. L., Natural photosensitizers in constructed unit process wetlands: Photochemical characterization and inactivation of pathogen indicator organisms. *Environmental Science & Technology* **2019,** *53*, (13), 7724-7735.
102. Grebel, J. E.; Pignatello, J. J.; Mitch, W. A., Sorbic acid as a quantitative probe for the formation, scavenging and steady-state concentrations of the triplet-excited state of organic compounds. *Water Research* **2011,** *45*, (19), 6535-6544.
103. Boudreau, B. P.; Ruddick, B. R., On a reactive continuum representation of organic matter diagenesis. *American Journal of Science* **1991,** *291*, (5), 507-538.
104. Mostovaya, A.; Koehler, B.; Guillemette, F.; Brunberg, A.-K.; Tranvik, L. J., Effects of compositional changes on reactivity continuum and decomposition kinetics of lake dissolved organic matter. *Journal of Geophysical Research: Biogeosciences* **2016,** *121*, (7), 1733-1746.
105. Koehler, B.; von Wachenfeldt, E.; Kothawala, D. N.; Tranvik, L. J., Reactivity continuum of dissolved organic carbon decomposition in lake water. *Journal of Geophysical Research: Biogeosciences* **2012,** *117*, (G1), G01024.
106. Mostovaya, A.; Hawkes, J. A.; Koehler, B.; Dittmar, T.; Tranvik, L. J., Emergence of the reactivity continuum of organic matter from kinetics of a multitude of individual molecular constituents. *Environmental Science & Technology* **2017,** *51*, (20), 11571-11579.
107. Westrich, J. T.; Berner, R. A., The role of sedimentary organic matter in bacterial sulfate reduction: The G model tested. *Limnology and Oceanography* **1984,** *29*, (2), 236-249.
108. Koehler, B.; Tranvik, L. J., Reactivity continuum modeling of leaf, root, and wood decomposition across biomes. *Journal of Geophysical Research: Biogeosciences* **2015,** *120*, (7), 1196-1214.
109. Aguer, J. P.; Richard, C.; Andreux, F., Comparison of the photoinductive properties of commercial, synthetic and soil-extracted humic substances. *Journal of Photochemistry and Photobiology A: Chemistry* **1997,** *103*, (1), 163-168.
110. Carlos, L.; Cipollone, M.; Soria, D. B.; Sergio Moreno, M.; Ogilby, P. R.; García Einschlag, F. S.; Mártire, D. O., The effect of humic acid binding to magnetite nanoparticles on the photogeneration of reactive oxygen species. *Separation and Purification Technology* **2012,** *91*, 23-29.
111. Chen, X.; Wang, J.; Chen, J.; Zhou, C.; Cui, F.; Sun, G., Photodegradation of 2-(2-hydroxy-5-methylphenyl)benzotriazole (UV-P) in coastal seawaters: Important role of DOM. *Journal of Environmental Sciences* **2019,** *85*, 129-137.
112. Guo, Z.; Wang, J.; Chen, X.; Cui, F.; Wang, T.; Zhou, C.; Song, G.; Zhang, S.; Chen, J., Photochemistry of dissolved organic matter extracted from coastal seawater: Excited triplet-states and contents of phenolic moieties. *Water Research* **2021,** *188*, 116568.

113. Leresche, F.; Torres-Ruiz, J. A.; Kurtz, T.; von Gunten, U.; Rosario-Ortiz, F. L., Optical properties and photochemical production of hydroxyl radical and singlet oxygen after ozonation of dissolved organic matter. *Environmental Science: Water Research & Technology* **2021,** *7*, (2), 346-356.
114. Tu, Y.; Liu, H.; Li, Y.; Zhang, Z.; Lei, Y.; Zhao, Q.; Tian, S., Radical chemistry of dissolved black carbon under sunlight irradiation: Quantum yield prediction and effects on sulfadiazine photodegradation. *Environmental Science and Pollution Research* **2021**, DOI: 10.1007/s11356-021-17379-5.
115. Liao, Z.; Wang, Y.; Xie, K.; Xie, N.; Cai, X.; Zhou, L.; Yuan, Y., Photochemistry of dissolved organic matter in water from the Pearl river (China): Seasonal patterns and predictive modelling. *Water Research* **2022,** *208*, 117875.
116. Wang, Z.; Lv, J.; Zhang, S.; Christie, P.; Zhang, S., Interfacial molecular fractionation on ferrihydrite reduces the photochemical reactivity of dissolved organic matter. *Environmental Science & Technology* **2021,** *55*, (3), 1769-1778.
117. Nkhili, E.; Boguta, P.; Bejger, R.; Guyot, G.; Sokołowska, Z.; Richard, C., Photosensitizing properties of water-extractable organic matter from soils. *Chemosphere* **2014,** *95*, 317-323.
118. Palma, D.; Sleiman, M.; Voldoire, O.; Beauger, A.; Parlanti, E.; Richard, C., Study of the dissolved organic matter (DOM) of the Auzon cut-off meander (Allier River, France) by spectral and photoreactivity approaches. *Environmental Science and Pollution Research* **2020,** *27*, (21), 26385-26394.
119. Pozdnyakov, I. P.; Salomatova, V. A.; Parkhats, M. V.; Dzhagarov, B. M.; Bazhin, N. M., Efficiency of singlet oxygen generation by fulvic acids and its influence on UV photodegradation of herbicide Amitrole in aqueous solutions. *Mendeleev Communications* **2017,** *27*, (4), 399-401.
120. Silva, M. P.; Lastre-Acosta, A. M.; Mostafa, S.; McKay, G.; Linden, K. G.; Rosario-Ortiz, F. L.; Teixeira, A. C. S. C., Photochemical generation of reactive intermediates from urban-waste bio-organic substances under UV and solar irradiation. *Environmental Science and Pollution Research* **2017,** *24*, (22), 18470-18478.
121. Wan, D.; Kong, Y.; Selvinsimpson, S.; Luo, F.; Chen, Y., Effect of UV254 disinfection on the photoformation of reactive species from effluent organic matter of wastewater treatment plant. *Water Research* **2020,** *185*, 116301.
122. Wan, D.; Wang, J.; Dionysiou, D. D.; Kong, Y.; Yao, W.; Selvinsimpson, S.; Chen, Y., Photogeneration of reactive species from biochar-derived dissolved black carbon for the degradation of amine and phenolic pollutants. *Environmental Science & Technology* **2021,** *55*, (13), 8866-8876.
123. Wan, D.; Wang, H.; Sharma, V. K.; Selvinsimpson, S.; Dai, H.; Luo, F.; Wang, C.; Chen, Y., Mechanistic investigation of enhanced photoreactivity of dissolved organic matter after chlorination. *Environmental Science & Technology* **2021,** *55*, (13), 8937-8946.
124. Wan, D.; Kong, Y.; Wang, X.; Selvinsimpson, S.; Sharma, V. K.; Zuo, Y.; Chen, Y., Effect of permanganate oxidation on the photoreactivity of dissolved organic matter for photodegradation of typical pharmaceuticals. *Science of the Total Environment* **2022,** *813*, 152647.
125. Wang, J.; Chen, J.; Qiao, X.; Wang, Y.; Cai, X.; Zhou, C.; Zhang, Y.; Ding, G., DOM from mariculture ponds exhibits higher reactivity on photodegradation of sulfonamide antibiotics than from offshore seawaters. *Water Research* **2018,** *144*, 365-372.
126. Wang, J.; Chen, J.; Qiao, X.; Zhang, Y.-n.; Uddin, M.; Guo, Z., Disparate effects of DOM extracted from coastal seawaters and freshwaters on photodegradation of 2,4-Dihydroxybenzophenone. *Water Research* **2019,** *151*, 280-287.
127. Zeng, Y.; Fang, G.; Fu, Q.; Dionysiou, D. D.; Wang, X.; Gao, J.; Zhou, D.; Wang, Y., Photochemical characterization of paddy water during rice cultivation: Formation of reactive intermediates for As(III) oxidation. *Water Research* **2021,** *206*, 117721.
128. Zhao, J.; Zhou, Y.; Li, C.; Xie, Q.; Chen, J.; Chen, G.; Peijnenburg, W. J. G. M.; Zhang, Y.-n.; Qu, J., Development of a quantitative structure-activity relationship model for mechanistic interpretation and quantum yield prediction of singlet oxygen generation from dissolved organic matter. *Science of the Total Environment* **2020,** *712*, 136450.

129. Zhou, C.; Xie, Q.; Wang, J.; Chen, X.; Niu, J.; Chen, J., Effects of dissolved organic matter derived from freshwater and seawater on photodegradation of three antiviral drugs. *Environmental Pollution* **2020,** *258*, 113700.
130. Zhou, C.; Chen, J.; Xie, H.; Zhang, Y.-n.; Li, Y.; Wang, Y.; Xie, Q.; Zhang, S., Modeling photodegradation kinetics of organic micropollutants in water bodies: A case of the Yellow River estuary. *Journal of Hazardous Materials* **2018,** *349*, 60-67.
131. Zhou, Y.; Cheng, F.; He, D.; Zhang, Y.-n.; Qu, J.; Yang, X.; Chen, J.; Peijnenburg, W. J. G. M., Effect of UV/chlorine treatment on photophysical and photochemical properties of dissolved organic matter. *Water Research* **2021,** *192*, 116857.
132. Zhou, S.; Liao, Z.; Zhang, B.; Hou, R.; Wang, Y.; Zhou, S.; Zhang, Y.; Ren, Z. J.; Yuan, Y., Photochemical behavior of microbial extracellular polymeric substances in the aquatic environment. *Environmental Science & Technology* **2021,** *55*, (22), 15090-15099.
